# Late-stage functionalisation of alkyne-modified phospha-xanthene dyes: lysosomal imaging using an off–on–off type of pH probe†

**DOI:** 10.1039/d1sc01705e

**Published:** 2021-04-30

**Authors:** Hiroaki Ogasawara, Yoshiki Tanaka, Masayasu Taki, Shigehiro Yamaguchi

**Affiliations:** Department of Chemistry, Graduate School of Science, Integrated Research Consortium on Chemical Sciences (IRCCS), Nagoya University Furo, Chikusa Nagoya 464-8602 Japan yamaguchi@chem.nagoya-u.ac.jp; Institute of Transformative Bio-Molecules (WPI-ITbM), Nagoya University Furo, Chikusa Nagoya 464-8601 Japan taki@itbm.nagoya-u.ac.jp

## Abstract

Near-infrared (NIR) fluorescent molecules are of great importance for the visualisation of biological processes. Among the most promising dye scaffolds for this purpose are P=O-substituted phospha-xanthene (POX) dyes, which show NIR emission with high photostability. Their practical utility for *in vitro* and *in vivo* imaging has recently been demonstrated. Although classical modification methods have been used to produce POX-based fluorescent probes, it is still a challenge to introduce additional functional groups to control the localisation of the probe in cells. Herein, we report on the development of POXs that bear a 4-ethynylphenyl group on the phosphorus atom. These dyes can subsequently be functionalised with azide-tagged biomolecules *via* a late-stage Cu-catalysed azide/alkyne cycloaddition (CuAAC) reaction, thus achieving target-selective labelling. To demonstrate the practical utility of the functionalised POXs, we designed a sophisticated NIR probe that exhibits a bell-shaped off–on–off pH-response and is able to assess the degree of endosomal maturation.

## Abstract 2

A series of NIR-emissive phospha-xanthene dyes bearing an ethynyl group are reported. The late-stage functionalisation of the NIR dyes enables creation of multi-functionalised fluorescent probes that can be designed to target organelles of interest.

## Graphical abstract

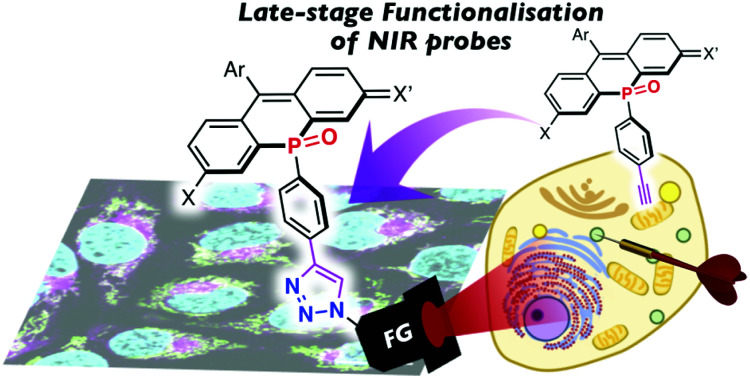

## Introduction

**Fig. 1:**
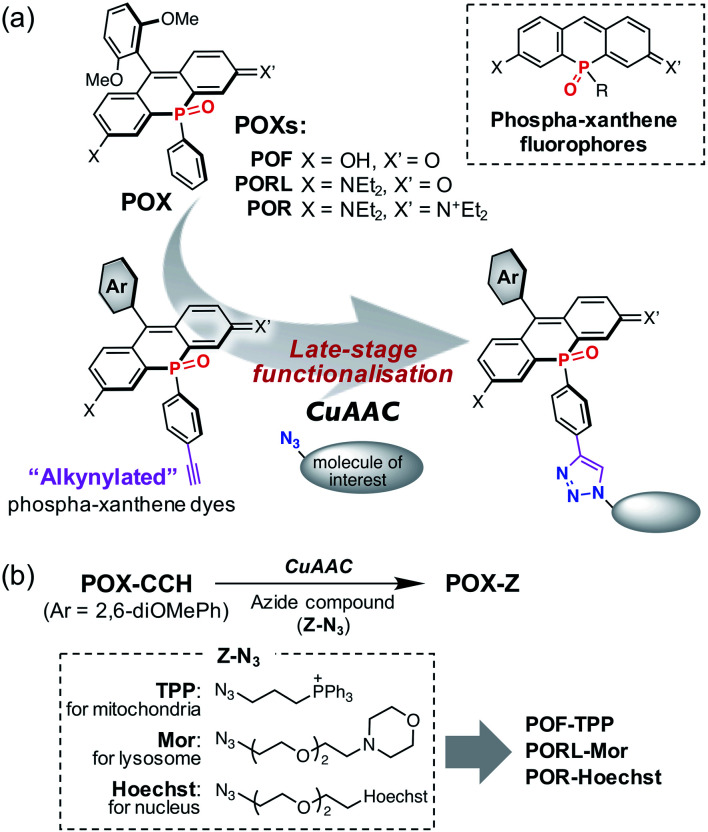
(a) Functionalisation of phospha-xanthene fluorophores shown in a dashed box. A phenyl ring on the phosphorus atom of the parent POX dyes is modified with an ethynyl group. The alkynylated phospha-xanthene dyes thus obtained can be converted into the corresponding triazole compounds *via* a copper-catalysed azide–alkyne cycloaddition (CuAAC) at the late stage of synthesis. (b) POX-based organelle markers obtained from alkynylated-POXs (POX-CCHs) and azide compounds used in this work.

The visualisation of specific cellular events at an organelle level can provide valuable information that aids in the understanding of complicated biological systems. Small-molecule-based fluorescent probes, which exhibit stimuli-induced fluorescence switching, are important chemical tools in the field of fluorescence imaging. They enable the acquisition of multi-dimensional images of biological functions occurring in living cells with a high spatiotemporal resolution.^1–6^ Fluorescent probes that have absorption and emission bands in the far-red to near-infrared (NIR) region are particularly useful because they exhibit negligible light-induced photodamage, show low interference with autofluorescence, and engage in minimal cross-talk with other dyes and fluorescent proteins.^7,8^ Accordingly, various NIR fluorescent probes based on fluorophores such as cyanine, aza-BODIPY, and xanthene have been developed.^9–14^ In most cases, these probes can be localised, depending on the intrinsic properties of the fluorophore, to one or more organelles in a cell. For instance, anionic dyes such as fluorescein diffuse into the cytosol, while cationic fluorophores such as rhodamine and cyanine tend to accumulate in the mitochondria.^15–17^ However, due to a low synthetic diversity in NIR fluorophores, it is still a challenge to develop biocompatible NIR probes with specific target localisation. This is a particular problem for those that use multiple fluorescent switching units for analysing complex and cooperative biological events.^18,19^ The ideal probe-design is one that allows the introduction of various organelle-targeting groups into a fluorophore skeleton during the later stages of synthesis without any perturbation of its properties, especially its sensing capabilities. To meet the aforementioned requirements, phospha-xanthene fluorophores shown in Fig. 1a, which have been developed as a new class of NIR dyes, are potentially suitable candidates.^20–29^ These dyes replace the endocyclic oxygen atom of the classical xanthene scaffold with an electron-withdrawing phosphine oxide (>P(=O)R) moiety. Phospha-xanthene dyes have been used as NIR-labelling reagents in both *in vitro* and *in vivo* optical imaging.^22,27,29^ Moreover, following molecular design strategies that have been established for classical xanthene dyes, several phospha-xanthene-based NIR fluorescent probes for the detection of H_2_O_2_,^30^ Cu^+^,^31^ Ca^2+^,^32,33^ pH changes,^34^ and membrane potentials^35^ have been reported. In this context, we have successfully developed a series of >P(=O)Ph-substituted dyes, POXs (Fig. 1a), including phospha-fluoresceins (POFs),^20^ phospha-rhodols (PORLs),^21^ and phospha-rhodamines (PORs).^22,23^ These POXs exhibit high brightness with absorption and emission maxima (*λ*_abs_/*λ*_em_) at 633/660, 670/700, and 712/740 nm in aqueous solution at pH 7.4, respectively. We have also shown that they exhibit exceptionally high stability to photo-irradiation, arising from the electron-withdrawing character of the P=O group, and demonstrated their practical utility for the long-term imaging of biological events.^22^ However, even in these POXs, as with the classical xanthene dyes, the modifiable sites are limited to the 9-aryl group and/or the O- and N-termini.^36,37^ Once these positions have been functionalised to become the sensing sites, it is difficult to subsequently introduce an additional functional group to enable control over the intracellular localisation.

To solve this problem, we propose a new series of NIR phospha-xanthene dyes, in which a 4-ethynylphenyl group is installed on the phosphorus atom (Fig. 1a). The terminal alkyne can be readily modified with a variety of azide-containing molecules of interest at the late stage of synthesis *via* a copper-catalysed azide–alkyne cycloaddition (CuAAC) without perturbation of the photophysical properties.^38–40^ We herein report the development of alkynylated POX dyes, POX-CCHs, and their functionalisation by CuAAC with various azide compounds (Fig. 1b). Regardless of the type of xanthene dye used, the subcellular localisation of the POX dyes can be precisely controlled using organelle-targeting labelling groups. Moreover, we have designed a NIR emissive pH probe with two p*K*_a_ values which exhibits an “off–on–off” fluorescence response when exposed to a change from neutral pH in the medium to the highly acidic luminal pH in the lysosomes. As a showcase of the practical utility of this probe, we have successfully visualised the luminal pH heterogeneity during endosome maturation in living cells.

## Results and discussion

### Synthesis of alkyne modified phospha-xanthene (POX) dyes

**Scheme 1:**
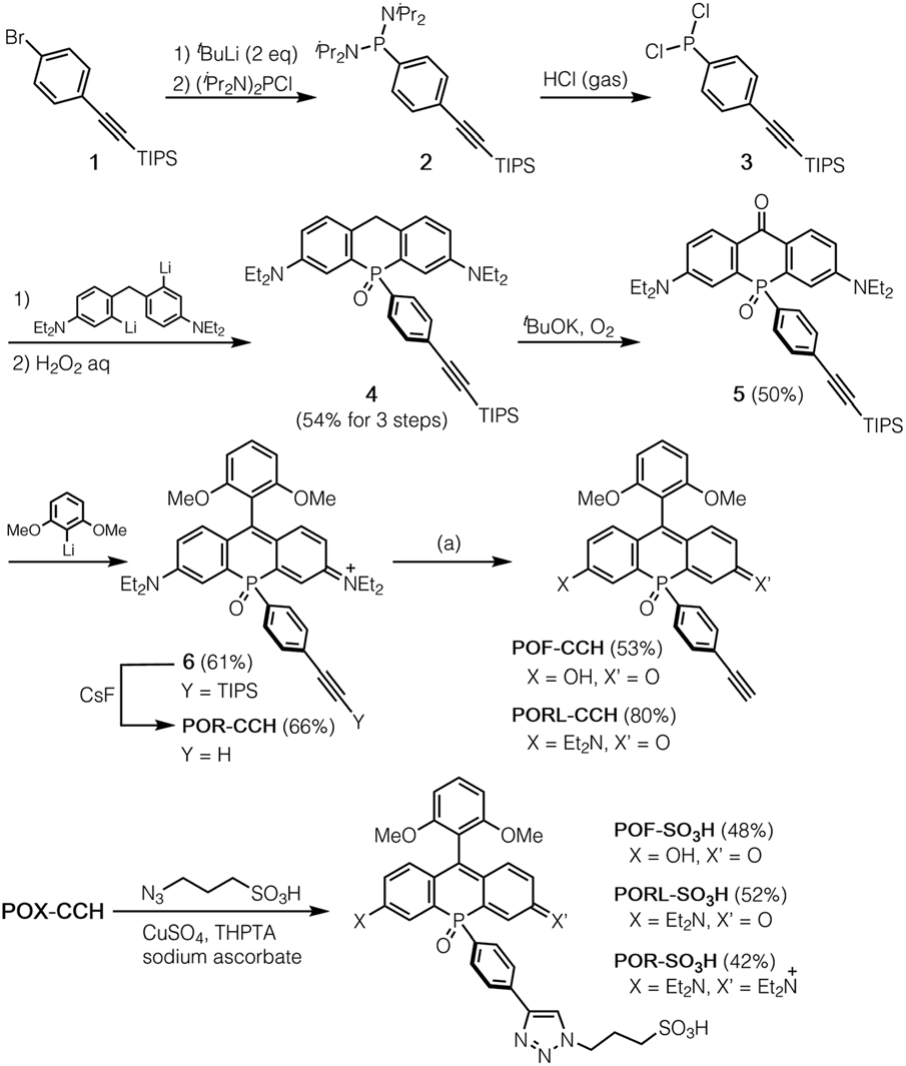
Synthesis of POX-CCHs and POX-SO_3_Hs. Reagents and conditions: (a) for POF-CCH: 1 M NaOH, H_2_O/CH_3_OH (1 : 1, v/v), 72 h; for PORL-CCH: 0.1 M NaOH, H_2_O/CH_3_OH (9 : 1, v/v), 3 h. TIPS = triisopropylsilyl. THPTA = tris[(hydroxypropyl-1*H*-1,2,3-triazol-4-yl)methyl]amine.

A method for the synthesis of the POX-CCH dyes is shown in Scheme 1, where *P*-ethynylphenyl-substituted phospha-xanthone **5** is a common key intermediate. This compound was readily synthesised from (4-bromophenyl)triisopropylsilylacetylene (**1**) in four steps. The lithiation of **1** with *t*BuLi, followed by treatment with bis(diisopropylamino)chlorophosphine produced phosphanylphenylacetylene **2**. Subsequent treatment of the crude mixture with dry HCl gas yielded moisture-sensitive dichloroarylphosphine **3**, which was used without purification in the next reaction with dilithiated bis(4-diethylamino-2-bromophenyl)methane. Further oxidation with an aqueous solution of H_2_O_2_ afforded P=O-bridged diarylmethane **4** in 54% overall yield over three steps. The benzylic position was then oxidised under an oxygen atmosphere in a basic solution to give phospha-xanthone **5**. A nucleophilic addition of 2,6-dimethoxyphenyllithium, followed by treatment with an aqueous solution of HCl afforded the corresponding silyl-protected phospha-rhodamine, which was then deprotected with CsF to give POR-CCH. Importantly, POR-CCH could be transformed into the corresponding rhodol PORL-CCH and fluorescein POF-CCH derivatives *via* a selective hydrolysis, the course of which depends on precise control over the basic conditions, as we have reported previously.^21^

### Functionalisation of alkyne modified POX dyes by CuAAC

As an example of the functionalisation of the POX-CCH dyes, we conjugated them with 3-azide-1-propanesulfonic acid by CuAAC in the presence of CuSO_4_, tris[(hydroxypropyl-1*H*-1,2,3-triazol-4-yl)methyl]amine (THPTA), and sodium ascorbate. The photophysical properties of the obtained POX-SO_3_H molecules are summarised in Table S1,† together with those of the parent POXs. Regardless of whether the triazole ring is present at the *para* position of the phenyl group or not, the absorption and emission spectra are almost identical under physiological conditions (50 mM HEPES, pH 7.4). This result indicates that the substituents on the *P*-phenyl group do not perturb the electronic structures of the phospha-xanthene skeletons. This finding was also supported by theoretical calculations on a triazole-derivative of POR-CCH, where the triazole ring has no direct participation in either the HOMO or the LUMO of this compound (Fig. S4†). It should be noted that the excellent photostabilities of the parent POXs are retained even after conversion into the triazole derivatives (Fig. S5†).^21,22^

**Fig. 2:**
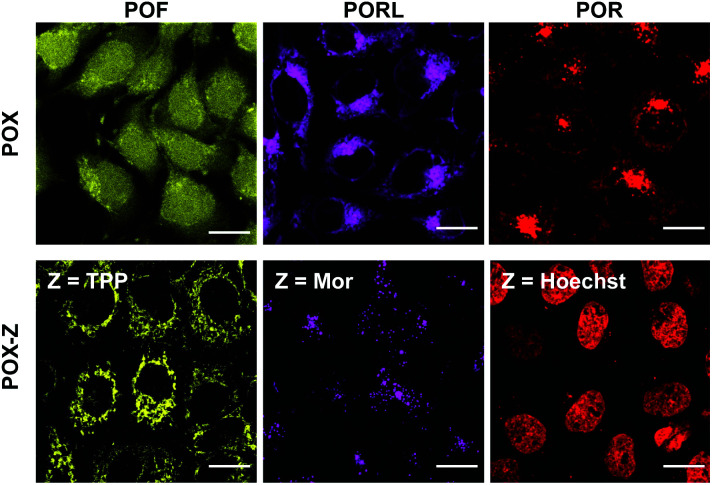
Fluorescence images of A431 cells stained with the original POXs (top) and with the POX-Zs functionalised with TPP, Mor, or Hoechst (bottom). POF, PORL, and POR are shown in yellow, magenta, and red, respectively; scale bar = 20 μm.

With the intention of labelling lysosomes, mitochondria, and cell nuclei, we synthesised a series of POX-Z dyes functionalised with the organelle-targeting groups such as morpholine (Mor), triphenylphosphonium (TPP), or bis-benzimide (Hoechst) (Fig. 1b). The imaging results obtained by using POF-TPP, PORL-Mor, and POR-Hoechst are shown in Fig. 2, together with those observed with the corresponding parent POXs. The staining patterns with functionalised POX-Z dyes show selective labelling of the target organelles (Fig. S17 and S18†), while the parent POX dyes diffuse or non-specifically accumulate in the cells. This reveals that, regardless of the inherent properties of the fluorophores, the subcellular localization of the dyes can be finely controlled by the functional groups introduced on the *P*-phenyl moiety. These results highlight the efficacy of late-stage functionalisation of the *P*-phenyl group for the development of a variety of POX-based fluorescent probes to monitor biological events at the subcellular level.

### Design and photophysical properties of the off–on–off type of pH probe

**Fig. 3:**
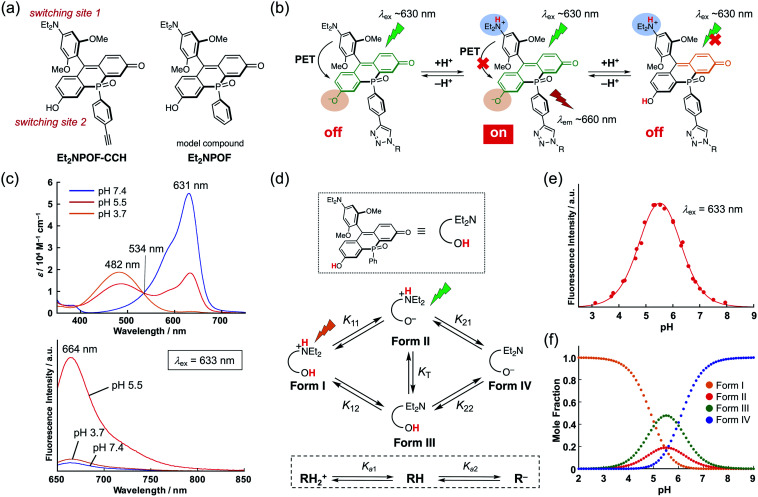
(a) Chemical structure of Et_2_NPOF-CCH and Et_2_NPOF. (b) Emissive protonated forms of the probe capable of being excited at ∼630 nm (left) and ∼480 nm (right). (c) Absorption and emission spectra (*λ*_ex_ = 633 nm) of Et_2_NPOF in pH-buffered solutions at pH 7.4 (blue), 5.5 (red), and 3.7 (orange). (d) Macroscopic and microscopic protonation equilibria for Et_2_NPOF. *K*_T_ is the equilibrium constant of tautomerisation. (e) A plot of fluorescence intensity at 660 nm (*λ*_ex_ = 633 nm) as a function of the pH value. The solid line is the result of curve fitting using the following p*K*_a_ values: p*K*_11_ = 5.45, p*K*_12_ = 5.05, p*K*_21_ = 5.57, and p*K*_22_ = 5.96. (f) Calculated species distributions as a function of the pH value.

To achieve a detailed examination of biological phenomena, it is desirable to furnish NIR-fluorescent probes with a complex fluorescence response, such as an “off–on–off” switching. However, in contrast to simple turn-on “off–on” probes, such fluorescent probes require a more sophisticated molecular design based on a multiple switching mechanism. This is hard to achieve due to the limited number of modifiable sites in ordinary xanthene skeletons. Indeed, although several types of bio-conjugate probe for monitoring pH changes in target subcellular regions have been reported, most of them are of the “off–on” or ratiometric type.^3,41–43^ While several pH sensors show an off–on–off response,^44–47^ they have critical limitations, such as an unsuitable pH sensing range, the need for UV-light excitation, and the lack of a site suitable for conjugation. Therefore, it remains a great challenge to develop pH-sensitive fluorescent sensors that satisfy all the aforementioned requirements. To solve this problem, we designed Et_2_NPOF-CCH, based on the POF fluorophore skeleton, with two fluorescence switching sites (Fig. 3a). In this system, the 9-aryl group, which contains a diethylamino (Et_2_N) moiety, acts as an electron donor in a photo-induced electron transfer (PET) mechanism, resulting in fluorescence quenching over a high pH range that prevents protonation of the Et_2_N group. The fluorescein OH group serves as the other site capable of switching in response to a pH change. Namely, the deprotonated POF exhibits an absorption band around ∼630 nm. When this band is excited, only the species in which the Et_2_N group is protonated and the fluorescein OH group is deprotonated can fluoresce at 660 nm. As the pH decreases, no fluorescence is observed following excitation around ∼630 nm, since the protonated POF only has an absorption band around ∼480 nm. Thus, the combination of the PET mechanism with the pH-dependence of the fluorescein skeleton furnishes an “off–on–off” fluorescence response upon pH changes (Fig. 3b).

To confirm the pH responsiveness of this fluorophore system, Et_2_NPOF, a compound without an alkyne group was examined (Fig. 3a). As expected, upon excitation at 633 nm, both an acidic (pH 3.7) and a neutral (pH 7.4) solution of this model compound showed weak fluorescence (Fig. 3c), while a 14-fold increase in emission intensity was observed at pH 5.5. The presence of biological nucleophiles such as glutathione in the mM range did not interfere with the pH response of Et_2_NPOF (Fig. S6†). The microscopic and macroscopic protonation equilibria of the amino and hydroxy groups of Et_2_NPOF are shown in Fig. 3d, where RH_2_^+^, RH, and R^−^ represent the cation (Form I), neutral species (Forms II and III), and anion (Form IV), respectively. In prototropic equilibria, Form II is the dominant emissive species when excited at 633 nm, while the fully protonated Form I also fluoresces following excitation at around 480 nm (right side of Fig. 3b). Using UV-vis absorption (Fig. S7†) and fluorescence (Fig. S8†) spectroscopic titration experiments and nonlinear curve fitting of the titration data (for details, see the ESI†), the four acid dissociation constants (microconstants) for the prototropic equilibria were determined to be p*K*_11_ = 5.45, p*K*_12_ = 5.05, p*K*_21_ = 5.57, and p*K*_22_ = 5.96. Based on these values, macroconstants (*K*_a_) and the tautomeric equilibrium constant (*K*_T_) were determined to be p*K*_a1_ = 4.91 (*K*_a1_ = *K*_11_ + *K*_12_), p*K*_a2_ = 6.11 (1/*K*_a2_ = 1/*K*_21_ + 1/*K*_22_), and *K*_T_ = 0.40 (Table S4†). Fig. 3e shows a plot of the fluorescence intensity upon excitation at 633 nm as a function of the pH value. The fluorescence intensity reaches its maximum at pH 5.5, implying that Et_2_NPOF is suitable for detecting physiological pH changes in a range between 4.5 and 6.5. Based on the microconstants, we calculated the species distribution at each pH value (Fig. 3f) and found that 19% of the total amount of Et_2_NPOF is present as the emissive Form II at pH 5.5. It is of note that, owing to the ample fluorescence quantum yield of Form I (*Φ*_F_ = 0.19 at pH 3), Et_2_NPOF would be a potential scaffold for the ratiometric measurement of pH changes in a dual excitation mode upon excitation at 488 nm and at the isosbestic point of 534 nm (Fig. 3c and S9†).

### Lysosomal imaging using an off–on–off type of pH probe

**Fig. 4:**
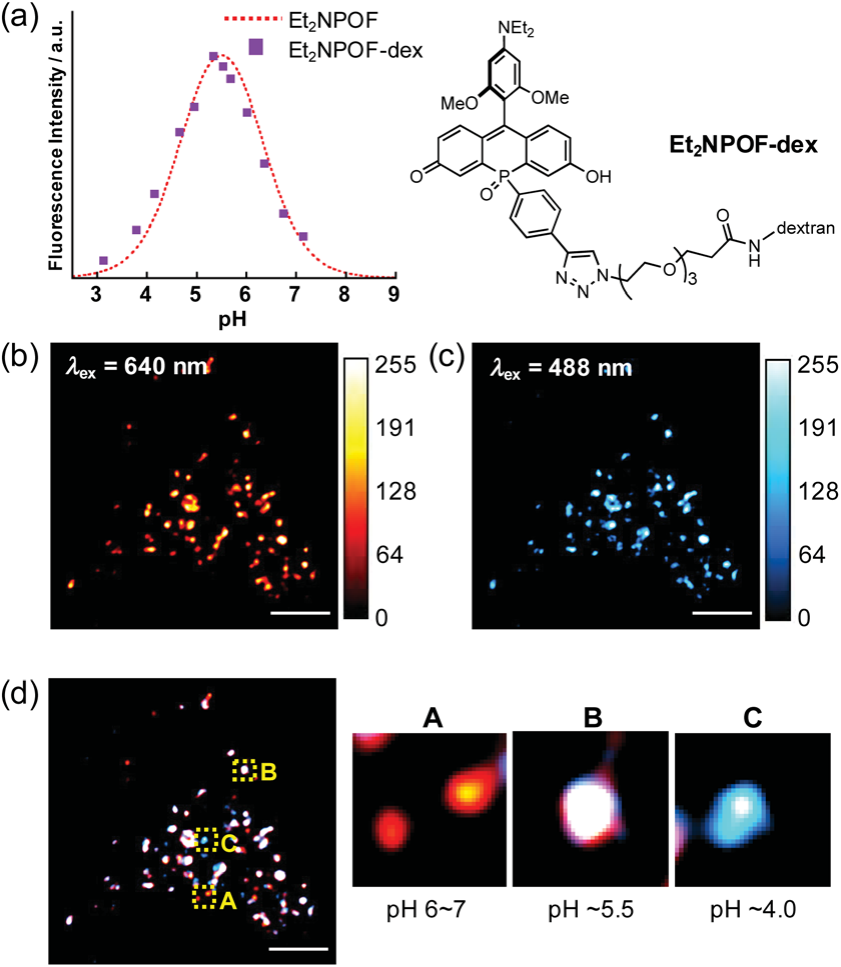
(a) pH-response of Et_2_NPOF-dex (purple squares) upon excitation at 630 nm shown with that of Et_2_NPOF (dotted line). (b and c) Endosomal images in HeLa cells incubated with Et_2_NPOF-dex. Images were acquired at 640 nm ((b), quasi-coloured in red) and 488 nm ((c), quasi-coloured in cyan) without washing the cells; scale bar = 5 μm. (d) Merged image of the two channels. The areas shown in yellow boxes are magnified on the right.

With the sensing capabilities of Et_2_NPOF-CCH established, dextran, a large-mass biomolecule, was introduced to the off–on–off pH probe *via* the late-stage functionalisation of the *P*-phenyl group. This probe was employed to visualise the pH changes in the cellular acidic compartments formed by endocytosis.^34,48^ To achieve the conjugation, amino dextran (MW = 10 kDa; Thermo Fisher Scientific) was initially treated with azido-PEG_3_-NHS ester, followed by a CuAAC reaction with Et_2_NPOF-CCH to form the target probe Et_2_NPOF-dex. The degree of labelling (DOL) in Et_2_NPOF-dex was calculated to be 1.1. Importantly, Et_2_NPOF-dex showed almost the same bell-shaped pH response as that observed for Et_2_NPOF, with the maximum fluorescence at pH 5.5 (Fig. 4a and S12†). HeLa cells were incubated in Dulbecco's modified Eagle's medium (DMEM) containing Et_2_NPOF-dex (2 mg mL^−1^) for 24 h. Images were recorded without washing at excitation wavelengths of 640 nm and 488 nm, which correspond to the wavelengths suitable for the excitation of Form II and Form I, respectively. Upon excitation at 640 nm, a punctate staining image was observed with intense fluorescence signals (Fig. 4b), in which each dot represents weak to moderately acidic compartments, such as lysosomes and endosomal vesicles, with a luminal pH value of around 4.5–6.5. The relatively intense spots are likely to be endosomes with pH ∼ 5.5. In contrast, the dots observed following excitation at 488 nm in Fig. 4c are likely to be more mature endosomes and highly acidic lysosomes with pH below 5.5. The difference in the staining patterns of these channels, which show a meaningfully low Pearson's correlation coefficient (*R* = 0.87, Fig. S22†), arises from the difference in pH values.

Considering the species distribution shown in Fig. 3f, the degree of endosomal maturation can be readily discriminated in the merged image (Fig. 4d).^49^ For example, a red dot observed in panel A can be attributed to a weakly acidic compartment with a luminal pH of around 6 to 7. In this pH range, the probe exists predominantly as Form II, while little Form I is present. Such dots should represent early endosomes and/or the recycling of endosomes. On the other hand, as the endosome matures and the luminal acidity increases, the red signal intensity, attributable to Form II, reaches a peak and eventually begins to decrease. Conversely, the intensity of the blue signals resulting from the formation of Form I increases during maturation. Thus, the punctate staining in white (panel B) and blue (panel C) in Fig. 4d is likely to be late endosomes (pH ∼ 5.5) and lysosomes (pH ∼ 4.0), respectively. It is also of great importance to note that low background fluorescence in the images was acquired without the need to wash the cells. This stands in sharp contrast to the intense background signals that arise from the medium observed when POF-dex was used (Fig. S23†). This is due to the efficient PET fluorescence quenching in the incubation solution caused by the off–on–off response of the Et_2_NPOF dye, a crucial advantage over conventional pH probes.

## Conclusions

In summary, we have developed a series of alkyne-modified phospha-xanthene dyes. A crucial advantage of the introduction of the >P(=O)Ph bridging moiety into the xanthene skeleton is that the phenyl group does not participate in the π-conjugation system and therefore this moiety can be used as a platform for additional functionalisation. Various groups for the targeted labelling of organelles were introduced at a late stage of the synthesis *via* a copper-catalysed azide–alkyne cycloaddition (CuAAC) reaction. The obtained NIR-emissive organelle markers were successfully applied to the selective staining of lysosomes, mitochondria, and cell nuclei. Functionalisation of the phospha-fluorescein POF scaffold with a large molecular mass biomolecule, amino dextran, together with the sophisticated design of an “off–on–off” pH probe, enabled us to produce a probe that can discriminate the degree of endosomal maturation. This dextran-conjugated probe will be an important tool for the understanding of important biological events such as autophagy.^50,51^ Throughout this research we have demonstrated a unique way of modifying phospha-xanthene dyes, which we believe will open up new avenues to create various types of useful NIR-emissive probe.

## Author contributions

M. T. and S. Y. conceived and led all aspects of the project. H. O. and Y. T. performed most of the experiments including the organic synthesis, spectroscopy measurements, and cell-imaging experiments. All authors wrote the manuscript.

## Conflicts of interest

There are no conflicts to declare.

## Supplementary Material

SC-012-D1SC01705E-s001
