# Supplementary material for: Late-stage functionalisation of alkyne-modified phospha-xanthene dyes: lysosomal imaging using an off–on–off type of pH probe
Source: Chem Sci. 2021 Apr 30;12(22):7902–7. doi: 10.1039/d1sc01705e (PMC8188471; doi:10.1039/d1sc01705e)
Supplement: SC-012-D1SC01705E-s001 [file SC-012-D1SC01705E-s001.pdf]

Electronic Supplementary Material (ESI) for Chemical Science.
This journal is © The Royal Society of Chemistry 2021

Electronic Supplementary Information

Late-stage Functionalisation of Alkyne-modified Phospha-Xanthene Dyes: Lysosomal Imaging Using an OFF-ON-OFF Type of pH Probe

Hiroaki Ogasawara,[†a] Yoshiki Tanaka,[†a] Masayasu Taki,[*b] and Shigehiro Yamaguchi[*a,b]

[a] Department of Chemistry, Graduate School of Science, and Integrated Research Consortium on Chemical Sciences (IRCCS), Nagoya University, Furo, Chikusa, Nagoya, 464–8602, Japan
[b] Institute of Transformative Bio–Molecules (WPI–ITbM), Nagoya University, Furo, Chikusa, Nagoya, 464–8601, Japan

E–mail: taki@itbm.nagoya–u.ac.jp, yamaguchi@chem.nagoya–u.ac.jp

Table of Contents

1. Experimental Procedures

1-1. Synthesis

1-2. Photophysical properties

Table S1. Photophysical data

1-3. Theoretical calculations

1.4. Evaluation of the photostability

1-5. Determination of acid dissociation constant (pK_a)

1-6. Preparation of dye-conjugated dextran

1-7. Cell experiments

2. Figures and tables

Figs. S1–S3 Absorption and emission spectra of POX dyes with and without a triazole ring

Fig. S4 Theoretical calculation results

Fig. S5 Photostability of POX-SO_3H dyes

Fig. S6 UV-vis absorption spectra of Et_2NPOF in the presence of GSH

Fig. S7 UV-vis absorption titration spectra of Et_2NPOF

Fig. S8 Fluorescence titrations of Et_2NPOF

Fig. S9 Fluorescence ratiometric titration

Fig. S10 UV-vis absorption spectra of Et_2NPOF-dex

Figs. S11–S12 Fluorescence spectra of Et_2NPOF-dex

Fig. S13 Cell viability determined by MTT assay

Figs. S14–S21 Confocal and bright-field images stained with the original POXs or *P*-functionalised POXs

Fig. S22 Visualisation of pH differences with Et_2NPOF-dex

Fig. S23 Imaging with POF-dex

Tables S2–S3. Cartesian coordinates of DFT optimised geometries

Table S4. Acidity constants of Et_2NPOF

3. References

4. NMR Spectra

1. Experimental Procedures

1.1 Synthesis

General information

^{1}H, $^{13}C\{^{1}H\}$, and $^{31}P\{^{1}H\}$ NMR spectra were recorded with a JEOL ECZ 400 spectrometer (400 MHz for ^{1}H, 100 MHz for $^{13}C\{^{1}H\}$, and 162 MHz for $^{31}P\{^{1}H\}$) and JEOL ECA 500 II spectrometer (500 MHz for ^{1}H, 125 MHz for $^{13}C\{^{1}H\}$, and 202 MHz for $^{31}P\{^{1}H\}$) in CD_3OD and $CDCl_3$. ^{1}H and $^{13}C\{^{1}H\}$ spectra were recorded with a JEOL ECA 600 II spectrometer equipped with an UltraCOOL probe (600 MHz for ^{1}H and 150 MHz for $^{13}C\{^{1}H\}$). The chemical shifts in ^{1}H NMR spectra are reported in δ ppm using the residual protons of the solvents as an internal standard ($CDCl_3$: δ 7.26 and CD_3OD: δ 3.31), and those in ^{13}C NMR spectra are reported using the solvent signals as an internal standard ($CDCl_3$: δ 77.16, CD_3OD: δ 49.00, and CD_2Cl_2/CD_3OD (9/1, v/v): δ 53.84). The chemical shifts in ^{31}P NMR spectra are reported using H_3PO_4 (δ 0.00) as an external standard. The high-resolution mass spectra were measured with a Thermo Fisher Scientific Exactive with the ESI ionisation method. Thin layer chromatography (TLC) was performed on glass plates coated with 0.25 mm thickness of silica gel $60F_{254}$ (Merck). Column chromatography was performed using silica gel 60 (Kanto Chemicals). HPLC purification was performed on a Delta 600 HPLC (Waters) or a preparative HPLC system (Shimadzu) using a C18 reverse phase column (YMC Triart C18, 10 × 250 mm or 20 × 250 mm). Unless otherwise noted, chemical reagents and solvents were purchased from commercial suppliers (Tokyo Chemical Industry (TCI), Sigma Aldrich, Kanto Chemicals, and FUJIFILM Wako Pure Chemical Corporation (Wako)) and used without further purification. Hoechst 33258·3HCl was purchased from Cosmo Bio Co., LTD. Anhydrous THF, toluene, CH_3CN, and DMF were purchased from Kanto Chemicals or Wako and further purified by Glass Contour Solvent Systems. [(4-bromophenyl)ethynyl]triisopropylsilane (**1**),[1] bis[2-bromo-4-(diethylamino)phenyl]methane,[2] 4-bromo-3,5-dimethoxyaniline,[3] 3,7-bis(diethylamino)-5-phenyl-10H-acridophosphin-10-one 5-oxide (*P*-xanthone),[4] 2-[2-(2-Azidoethoxy)ethoxy]ethyl *p*-toluenesulfonate,[5] (3-azidopropyl)triphenylphosphonium bromide (N_3-TPP),[6] 3-azidopropane-1-sulfonic acid,[7] and AcPOF[8] were synthesised according to the literature methods.

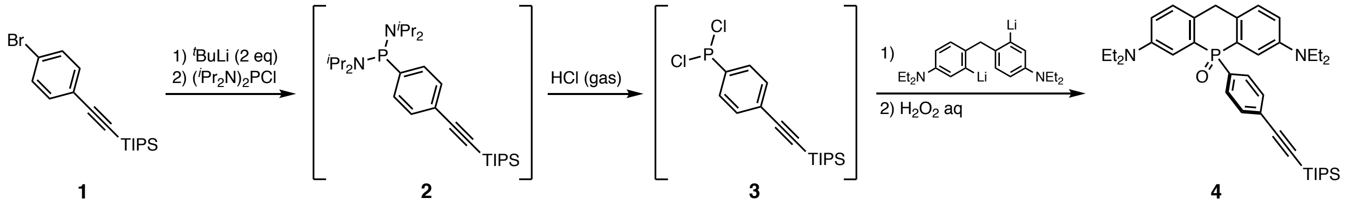

1 2 3 4

Compound 4. To a solution of [(4-bromophenyl)ethynyl]triisopropylsilane (**1**)[1] (4.93 g, 14.6 mmol) in anhydrous THF (60 mL) was added *tert*-BuLi (1.62 M in *n*-pentane, 18.0 mL, 29.2 mmol) at –78 °C and the mixture was stirred at the same temperature for 40 min, then at –20 °C for 10 min. To the solution, bis(diisopropylamino)chlorophosphine (0.86 M in THF, 17.0 mL, 14.6 mmol) was added dropwise at –78 °C. After the mixture was warmed up to room temperature and stirred for 10 h, all the volatiles were removed under reduced pressure to afford bis(diisopropylamino)[4-(triisopropylsilylethynyl)phenyl]phosphine (**2**) as a viscous oil. The conversion was confirmed by ^{1}H and ^{31}P NMR spectra (see 4. NMR Spectra in the ESI). All the obtained compounds were used for the next reaction without further purification.

The viscous oil of **2** thus obtained was suspended in anhydrous toluene (60 mL). Dry HCl, generated by adding concentrated HCl (37% wt) to concentrated H_2SO_4 followed by passing through a concentrated H_2SO_4 trap for drying, was bubbled into the suspension at 0 °C until the gas was no longer dissolved. The resulting suspension was filtered through Celite, and the volatiles were removed under reduced pressure to give [4-(triisopropylsilylethynyl)phenyl]phosphine dichloride **(3)** as a yellow viscous oil. The conversion was confirmed by ^{1}H and ^{31}P NMR spectra (see 4. NMR Spectra in the ESI). All the obtained compounds were dissolved in anhydrous THF (20 mL) and the resulting solution was used for the next reaction.

To a solution of bis[2-bromo-4-(diethylamino)phenyl]methane[2] (6.83 g, 14.6 mmol) in anhydrous THF (50 mL) was added *sec*-BuLi (1.01 M in cyclohexane and *n*-hexane, 29.0 mL, 29.2 mmol) at –78 °C and the mixture was stirred at the same temperature for 1 h. A solution of **3** in THF was slowly added at –78 °C and the resulting solution was allowed to warm up to room temperature. After stirring at room temperature for 15 h, a 30% aqueous solution of H_2O_2 (4 mL) was added at 0 °C. The mixture was stirred for 30 min and unreacted H_2O_2 was then quenched with an aqueous solution of Na_2SO_3 (15 mL) at 0 °C. The organic layer was separated, and the aqueous layer was extracted with CH_2Cl_2 (30 mL × 3). The combined organic layer was washed with an aqueous solution of Na_2SO_3 (20 mL) and brine, dried over Na_2SO_4, and concentrated. The resulting pale yellow solid was purified by silica-gel column chromatography eluted with CH_2Cl_2/acetone by a stepwise increase in the volume of acetone (19:1 to 9:1, v/v) to give **4** (4.81 g, 7.85 mmol, 54% for 3 steps) as a pale yellow solid. ^{1}H NMR ($CDCl_3$, 500 MHz) δ 7.46–7.37 (m, 6H), 7.17 (dd, J = 8.4, 6.1 Hz, 2H), 6.76 (dd, J = 8.4, 2.5 Hz, 2H), 3.81 (d, J = 18.2 Hz, 1H), 3.63 (dd, J = 18.2, 3.0 Hz, 1H), 3.44–3.33 (m, 8H), 1.15 (t, J = 7.3 Hz, 12H), 1.09–1.07 (m, 21H). ^{13}C NMR ($CDCl_3$, 125 MHz) δ 146.8 (d, J = 12.0 Hz, C), 134.9 (d, J = 103.1 Hz, C), 131.9 (d, J = 12.0 Hz, CH), 130.6 (d, J = 10.9 Hz, CH), 129.4 (d, J = 99.5Hz, C), 129.2 (d, J = 12.0 Hz, CH), 128.2 (d, J = 8.4 Hz, C), 126.3 (s, C), 115.3 (s, CH), 113.3 (d, J = 8.4 Hz, CH), 106.4 (s, C), 93.2 (s, C), 44.4 (s, CH_2), 35.2 (d, J = 8.5 Hz, CH_2), 18.7 (s, CH_3), 12.6 (s, CH_3), 11.3 (s, CH). ^{31}P NMR ($CDCl_3$, 162 MHz) δ 14.0. HRMS (ESI) *m/z* calcd. for $C_{38}H_{54}N_2OSiP$ $[M+H]^+$: 613.3743; found: 613.3760.

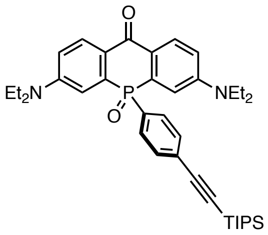

5

Compound 5. To a solution of **4** (3.04 g, 4.97 mmol) in anhydrous THF (80 mL) was added a solution of potassium *tert*-butoxide (570 mg, 5.08 mmol) in anhydrous THF (20 mL) at room temperature under an oxygen atmosphere. The reaction mixture was stirred for 1.5 h at room temperature, and then quenched with a 1 M aqueous solution of HCl (3.0 mL). To the solution, 30 mL of water was added and the aqueous layer was extracted with ethyl acetate (30 mL × 3). The combined organic layer was washed with brine, dried over Na_2SO_4, and concentrated. The resulting mixture was purified silica-gel column chromatography eluted with CH_2Cl_2/acetone by a stepwise increase in the volume of acetone (19:1 to 13:1, v/v) to afford 1.55 g (2.48 mmol, 50%) of **5** as a yellow solid. ^{1}H NMR ($CDCl_3$, 400 MHz) δ 8.27 (dd, J = 9.1, 6.4 Hz, 2H), 7.53 (dd, J = 12.4, 8.0 Hz, 2H), 7.40 (dd, J = 8.0, 2.6 Hz, 2H), 7.07 (dd, J = 14.8, 2.8 Hz, 2H), 6.82 (dd, J = 9.1, 2.8 Hz, 2H) 3.50–3.34 (m, 8H), 1.16 (t, J = 7.2 Hz, 12H), 1.09–1.06 (m, 21H). ^{13}C NMR ($CDCl_3$, 100 MHz) δ 180.0 (d, J = 8.6 Hz, C), 150.5 (d, J = 13.4 Hz, C), 135.3 (d, J = 105.4 Hz, C), 134.6 (d, J = 96.8 Hz, C), 132.1 (d, J = 12.5Hz, CH), 131.6 (d, J = 9.5 Hz, CH), 130.3 (d, J = 10.6 Hz, CH), 126.7 (d, J =2.8 Hz, C), 123.9 (d, J = 6.7 Hz, C), 114.4 (s, CH), 111.4 (d, J = 7.6 Hz, CH), 106.2 (s, C), 93.7 (s, C), 44.7 (s, CH_2), 18.7 (s, CH_3), 12.6 (s, CH_3), 11.3 (s, CH). ^{31}P NMR ($CDCl_3$, 162 MHz) δ 6.6. HRMS (ESI) m/z calcd. for $C_{38}H_{51}N_2O_2SiPNa$ $[M+Na]^+$: 649.3355; found: 649.3350.

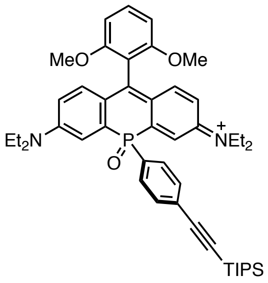

6

Compound 6. To a solution of 2-bromo-1,3-dimethoxybenzene (65.1 mg, 0.300 mmol) in anhydrous THF (3.0 mL) was added *tert*-BuLi (1.56 M in *n*-pentane, 0.39 mL, 0.60 mmol) at –78 °C. The resulting solution was stirred for 1 h at the same temperature and then at 0 °C for 5 min. A solution of **5** (62.7 mg, 0.100 mmol) in anhydrous THF (5.0 mL) was added dropwise at –78 °C over 10 min. The reaction mixture was allowed to warm to room temperature and was further stirred for 4 h. To the solution, 10 mL of a 0.05 M aqueous solution of HCl was carefully added and the mixture was stirred for 30 min. The resulting dark green solution was diluted with water (20 mL) and washed with hexane (15 mL × 2), then extracted six times with CH_2Cl_2 (15 mL × 6). The combined organic layer was washed with brine, dried over Na_2SO_4, and concentrated. The resulting mixture was purified by silica-gel column chromatography eluted with CH_2Cl_2/methanol by a stepwise increase in the volume of methanol (19:1 to 9:1, v/v) to afford 47.8 mg (61.0 μmol, 61%) of **6** as a dark green solid. ^{1}H NMR (CD_3OD, 500 MHz) δ 7.80 (dd, J = 12.0, 8.5 Hz, 2H), 7.62–7.54 (m, 5H), 7.28 (dd, J = 10.0, 6.0 Hz, 2H), 6.96–6.88 (m, 4H), 3.78–3.67

(m, 14H), 1.27 (t, J = 6.8 Hz, 12H), 1.14–1.10 (m, 21H). ^{13}C NMR (CD_3OD, 125 MHz) δ 159.1 (s, C), 158.8 (s, C), 155.1 (d, J = 13.3 Hz, C), 141.3 (d, J = 8.4 Hz, CH), 138.9 (d, J = 94.8 Hz, C), 133.9 (d, J = 108 Hz, C), 133.5 (d, J = 13.1 Hz, CH) 133.3 (s, CH), 131.0 (d, J = 10.9 Hz, CH), 129.3 (d, J = 3.6 Hz, C), 125.1 (d, J = 6.0 Hz, C), 120.0 (d, J = 7.1 Hz, CH), 116.9 (s, CH), 113.8 (s, C), 106.9 (s, C), 105.3 (s, CH), 105.2 (s, CH), 95.6 (s, C), 56.9 (s, CH_3), 56.6 (s, CH_3), 47.5 (CH_2), 19.0 (s, CH_3), 13.1 (s, CH_3), 12.4 (s, CH). One C peak was overlapped. ^{31}P NMR (CD_3OD, 162 MHz) δ 8.9. HRMS (ESI) *m/z* calcd. for $C_{46}H_{60}N_2O_3SiP$ $[M]^+$: 747.4105; found: 747.4103.

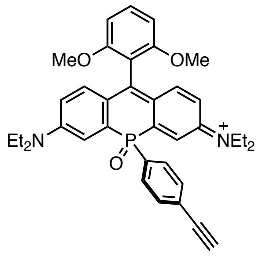

POR-CCH

POR-CCH. To a solution of **6** (134 mg, 0.171 mmol) in DMSO (5.0 mL) and methanol (1.0 mL), CsF (121 mg, 0.799 mmol) and acetic acid (48 mg, 0.799 mmol) in distilled water (1.0 mL) was added at room temperature. After stirring at 60 °C for 1.5 h, 15 mL of water was added to the reaction mixture and the aqueous layer was washed with toluene (15 mL × 3). The combined organic layer was then extracted with water (15 mL). All the aqueous layers were combined and extracted with CH_2Cl_2 (15 mL × 5). The combined organic layer was washed with brine, dried over Na_2SO_4, and concentrated. The resulting mixture was purified by silica-gel column chromatography eluted with CH_2Cl_2/methanol (10:1, v/v), followed by recrystallisation from a mixture of CH_2Cl_2/methanol/Et_2O at room temperature to afford 70.9 mg (0.113 mmol, 66 %) of POR-CCH as a dark green powder. ^{1}H NMR (CD_3OD, 500 MHz) δ 7.79 (dd, J = 10.5, 3.5 Hz, 2H), 7.63–7.53 (m, 5H), 7.27 (dd, J = 9.5, 6.5 Hz, 2H), 6.94 (dd, J = 9.5, 2.5 Hz, 2H), 6.90 (dd, J = 8.3, 6.3 Hz, 2H), 3.80–3.67 (m, 15H), 1.27 (t, J = 7.0 Hz, 12H). ^{13}C NMR (CD_3OD, 125 MHz) δ 159.2 (s, C), 158.8 (s, C), 155.1 (d, J = 13.3 Hz, C), 141.3 (d, J = 8.5 Hz, CH), 138.9 (d, J = 95.4 Hz, C), 134.1 (d, J = 108.6 Hz, C), 133.8 (d, J = 13.3 Hz, CH), 133.3 (s, CH), 130.9 (d, J = 10.9 Hz, CH), 128.7 (d, J = 3.6 Hz, C), 125.1 (d, J = 6.0 Hz, C), 120.0 (d, J = 7.2 Hz, CH), 116.9 (s, CH), 113.8 (s, C), 105.3 (s, CH), 105.2 (s, CH), 83.0 (s, C), 82.6 (s, C), 56.8 (s, CH_3), 56.6 (s, CH_3), 47.5 (s, CH_2), 13.1 (s, CH_3). One CH peak was overlapped. ^{31}P NMR (CD_3OD, 162 MHz) δ 8.9. HRMS (ESI) *m/z* calcd. for $C_{37}H_{40}N_2O_3P$ $[M]^+$: 591.2771; found: 591.2776.

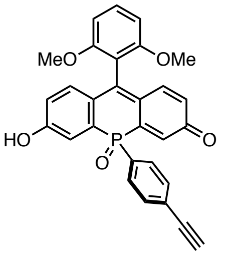

POF-CCH

POF-CCH. To a solution of POR-CCH (8.0 mg, 13 μmol) in methanol (2.0 mL) was added a 2 M aqueous solution of NaOH (2.0 mL) and the reaction mixture was stirred at room temperature for 4 days. After addition of water (10 mL), the resulting suspension was washed with CH_2Cl_2 (15 mL × 3). The aqueous layer was acidified with a 2 M

aqueous solution of HCl and extracted with ethyl acetate (15 mL × 4). The combined organic layer was washed with brine, dried over Na_2SO_4, and concentrated. The resulting mixture was purified by reversed phase HPLC eluted with a gradient of CH_3CN/H_2O containing 0.1% TFA to afford POF-CCH (3.3 mg, 6.9 µmol, 53%) as a red solid. ^{1}H NMR (CD_3OD, 400 MHz) δ 7.73 (dd, J = 12.2, 8.6 Hz, 2H), 7.61 (dd, J = 8.6 Hz, 2.8 Hz, 2H), 7.56 (t, J = 8.8 Hz, 1H), 7.21 (dd, J = 16.2, 2.1 Hz, 2H), 7.11 (dd, J = 9.5, 6.9 Hz, 2H), 6.87 (t, J = 8.8 Hz, 2H), 6.59 (brd, J = 9.5 Hz, 2H), 3.76 (s, 3H), 3.75 (s, 3H), 3.72 (s, 1H). ^{13}C NMR (CD_2Cl_2/CD_3OD = 9/1, 125 MHz) δ 158.3 (s, C), 157.9 (s, C), 150.1 (d, J = 7.2 Hz, C), 138.1 (brs, C), 133.9 (d, J = 108.6 Hz, C), 132.8 (d, J = 13.3 Hz, CH), 131.7 (s, CH), 130.4 (d, J = 10.9 Hz, CH), 127.5 (brs, C), 126.73 (s, C), 126.70 (s, C), 124.5 (brs, C), 113.7 (s, C), 104.4 (s, CH), 104.3 (s, CH), 82.7 (s, CH), 80.5 (s, CH), 56.5 (s, CH_3), 56.3 (s, CH_3). Two CH peaks were overlapped. ^{31}P NMR (CD_3OD, 162 MHz) δ 8.0. HRMS (ESI) *m/z* calcd. for $C_{29}H_{20}O_5P$ $[M–H]^-$: 479.1054; found: 479.1054.

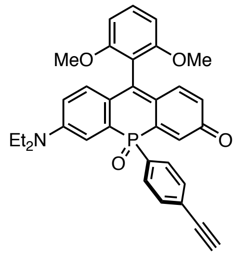

PORL-CCH

PORL-CCH. To a solution of POR-CCH (59.5 mg, 94.9 µmol) in methanol (3.0 mL) and distilled water (23.0 mL) was added a 0.75 M aqueous solution of NaOH (4.0 mL). After stirring for 3 h, 10 mL of water was added and the aqueous layer was extracted with CH_2Cl_2 (15 mL × 7). The combined organic layer was washed with brine, dried with Na_2SO_4, and concentrated. The resulting mixture was dissolved in a 1:1 solution of water and CH_3CN containing 0.1% TFA, followed by filtration through Presep® (Wako, ODS). The filtrate was purified by reversed phase HPLC eluted with a gradient of CH_3CN/H_2O containing 0.1% TFA to afford a dark blue solid of **PORL-CCH** (40.5 mg, 75.6 µmol, 80%). ^{1}H NMR (CD_3OD, 400 MHz) δ 7.80–7.73 (m, 3H), 7.64–7.59 (m, 3H), 7.45 (dd, J = 15.0 Hz, 2.8 Hz, 1H), 7.39 (dd, J = 10.1, 5.8 Hz, 1H), 7.30 (dd, J = 9.2, 6.7 Hz, 1H), 7.09 (dd, J = 10.0 Hz, 2.4 Hz, 1H), 6.96–6.89 (m, 3H), 3.95–3.85 (m, 4H), 3.77 (s, 1H), 3.76 (s, 3H), 3.75 (s, 3H), 1.34 (t, J = 7.3 Hz, 6H). ^{13}C NMR (125 MHz, CD_3OD) δ 166.5 (d, J = 14.5 Hz, C), 162.7 (s, C), 159.1 (s, C), 158.8 (s, C), 157.8 (d, J = 13.3 Hz, C), 144.2 (d, J = 7.2 Hz, CH), 143.2 (d, J = 93.0 Hz, C), 139.7 (d, J = 10.9 Hz, CH), 135.3 (d, J = 99.0 Hz, C), 133.8 (d, J = 13.3 Hz, CH), 133.7 (s, C), 132.8 (d, C, coupling partner is overlapped with 133.8–133.7 peaks), 131.2 (d, J = 10.9 Hz, CH), 129.1 (brs, C), 128.8 (d, J = 2.4 Hz, C), 127.4 (d, J = 7.2 Hz, C), 124.2 (d, J = 7.2 Hz, CH), 122.0 (s, CH), 121.7 (d, J = 4.8 Hz, CH), 119.0 (s, CH), 113.5 (s, C), 105.4 (s, CH), 105.3 (s, CH) , 83.0 (s, CH), 82.7 (s, CH), 56.8 (s, CH_3), 56.6 (s, CH_3), 49.3 (s, CH_2), 13.5 (s, CH_3). ^{31}P NMR (CD_3OD, 162 MHz) δ 7.7. HRMS (ESI) *m/z* calcd. for $C_{33}H_{30}NO_4P$ $[M+H]^+$: 536.1985; found: 536.1986.

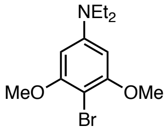

7

4-Bromo-*N*,*N*-diethyl-3,5-dimethoxyaniline (7). A mixture of 4-bromo-3,5-dimethoxyaniline[3] (1.17 g, 5.25 mmol), iodoethane (1.7 mL, 21.0 mmol), and K_2CO_3 (2.90 g, 21.0 mmol) in CH_3CN (30 mL) was refluxed for 36 h under a nitrogen atmosphere. After the reaction mixture was cooled to room temperature, the inorganic salts were removed by filtration and the filtrate was concentrated. The resulting mixture was dissolved in a small amount of CH_2Cl_2, and hexane was added to form precipitates, which were collected by filtration to give **7** (1.42 g, 4.91 mmol, 93%). Because this compound is highly air sensitive, it was used for the next step immediately after the synthesis. ^{1}H NMR ($CDCl_3$, 400 MHz) δ 5.92 (s, 2H), 3.86 (s, 6H), 3.35 (q, J = 7.2 Hz, 4H), 1.19 (t, J = 7.2Hz, 6H). ^{13}C NMR ($CDCl_3$, 100 MHz) δ 157.8 (s, C), 148.6 (s, C), 90.1 (s, CH), 87.1 (s, C), 56.4 (s, CH_3), 44.9 (s, CH_2), 12.7 (s, CH_3). HRMS (ESI): m/z calcd. for $C_{12}H_{19}NO_2{}^{79}Br$ $[M+H]^+$: 288.0599; found: 288.0592.

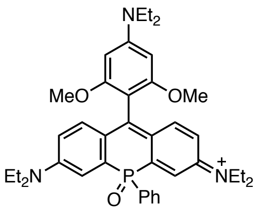

Et2NPOR

Et_2NPOR. To a solution of **7** (145 mg, 0.503 mmol) in anhydrous THF (5.0 mL) was added *tert*-BuLi (1.62 M in *n*-pentane, 0.62 mL, 1.00 mmol) at –78 °C. The resulting solution was stirred at the same temperature for 2 h and then at 0 °C for 10 min. A solution of *P*-xanthone[4] (62.7 mg, 0.100 mmol) in anhydrous THF (5.0 mL) was added dropwise at –78 °C over 10 min. The reaction mixture was allowed to warm to room temperature and was further stirred for 14 h. To the solution, 10 mL of a 0.1 M aqueous solution of HCl was carefully added and the mixture was stirred for 30 min. The resulting dark green solution was diluted with 15 mL of water and washed with toluene (20 mL × 2), then extracted with CH_2Cl_2 (20 mL × 4). The combined organic layer was washed with brine, dried over Na_2SO_4, and concentrated. The resulting mixture was purified by silica-gel column chromatography eluted with CH_2Cl_2/methanol (9:1, v/v), followed by recrystallisation from a mixture of CH_2Cl_2/Et_2O at room temperature to obtain Et_2NPOR (20.5 mg, 28.8 μmol, 14 %) as a dark green powder. ^{1}H NMR (CD_3OD, 400 MHz) δ 7.79 (dd, J = 12.8, 7.2 Hz, 2H), 7.66–7.60 (m, 2H), 7.59–7.51 (m, 3H), 7.29–7.10 (m, 4H), 6.97 (dd, J = 9.5, 2.8, 2H), 3.86 (s, 3H), 3.84 (s, 3H), 3.82 (q, J = 7.0 Hz, 4H), 3.74 (q, J = 7.2 Hz, 8H), 1.35 (t, J = 7.0 Hz, 6H), 1.28 (t, J = 7.2 Hz, 12H), ^{13}C NMR (CD_3OD, 125 MHz) δ 160.4 (s, C), 160.1 (s, C), 155.1 (d, J = 13.1 Hz, C), 140.7 (d, J = 8.4 Hz, CH), 139.4 (d, J = 94.9 Hz, C), 134.4 (s, CH), 133.7 (d, J = 108.0 Hz, C), 130.8 (d, J = 10.9 Hz, CH), 130.6 (d, J = 13.3 Hz, CH), 129.0 (s, C), 128.8 (s, C), 128.6 (s, C), 124.7 (brs, C), 120.4 (d, J = 7.3 Hz, CH), 117.0 (s, CH), 57.6 (s, CH_3), 57.4 (s, CH_3), 47.6 (s, CH_2), 13.1 (s, CH_3), 11.1 (s, CH_3). One CH peak and one CH_2 peak were overlapped. ^{31}P NMR (CD_3OD, 162 MHz) δ 9.5. HRMS (ESI) m/z calcd. for $C_{39}H_{49}N_3O_3P$ $[M]^+$: 638.3506; found: 638.3497.

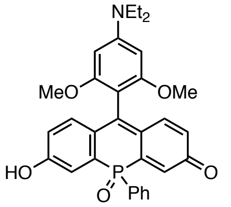

Et2NPOF

Et₂NPOF. To a solution of **Et₂NPOR** (10.0 mg, 11.5 μmol) in methanol (9.0 mL) was added a 4.5 M NaOH aqueous solution (3.0 mL). The reaction mixture was stirred at room temperature for 3 days. After addition of water (10 mL), the aqueous layer was washed with CH_2Cl_2 (15 mL × 3). The aqueous layer was acidified with 1 M HCl and extracted with CH_2Cl_2 (20 mL × 3). The organic layers were then washed with brine, dried over Na_2SO_4, and concentrated. The resulting mixture was dissolved in a 1:1 solution of water and CH_3CN containing 0.1% TFA, followed by filtration through Presep® (Wako, ODS). The filtrate was purified by reversed phase HPLC eluted with a gradient of CH_3CN/H_2O containing 0.1% TFA to afford **Et₂NPOF** (4.4 mg, 6.9 μmol, 60%) as a red solid. ^{1}H NMR (CD_3OD, 400 MHz) δ 7.74 (dd, J = 12.8, 6.7 Hz, 2H), 7.59 (t, J = 6.7 Hz, 1H), 7.52 (td, J = 6.7, 3.3 Hz, 2H), 7.22–7.15 (m, 4H), 6.68–6.58 (m, 4H), 3.79 (s, 6H), 3.68 (q, J = 7.1Hz, 4H), 1.30 (t, J = 7.1 Hz, 6H). ^{13}C NMR (CD_3OD, 100 MHz) δ 174.4 (d, J = 13.5 Hz, C), 160.3 (s, C), 149.5 (d, J = 7.7 Hz, C), 142.4 (s, C), 138.7 (d, J = 9.6 Hz, CH), 137.4 (d, J = 94.4 Hz, C), 134.1 (s, CH), 133.7 (d, J = 109.8 Hz, C), 131.0 (d, J = 10.6 Hz, CH), 130.3 (d, J = 13.5 Hz, CH), 127.94 (s, C), 127.89 (s, C) 127.8 (s, CH), 125.2 (s, CH), 115.4 (s, C), 99.5 (s, CH), 57.4 (s, CH_3), 54.4 (s, CH_2), 11.0 (s, CH_3). One CH peak was overlapped. ^{31}P NMR (CD_3OD, 162 MHz) δ 8.6. HRMS (ESI) m/z calcd. for $C_{31}H_{29}NO_5P$ [M–H]$^-$: 526.1783; found: 526.1783.

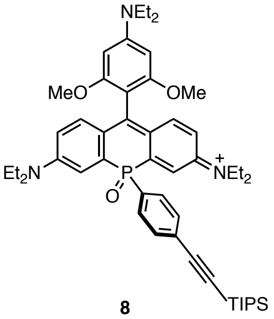

8

Compound 8. To a solution of **7** (338 mg, 1.18 mmol) in anhydrous THF (15.0 mL) was added *tert*-BuLi (1.56 M in *n*-pentane, 1.51 mL, 1.13 mmol) at –78 °C. The resulting solution was stirred at the same temperature for 1 h and then at –20 °C for 10 min. A solution of **5** (184.3 mg, 0.294 mmol) in anhydrous THF (6.0 mL) was added dropwise at –78 °C over 10 min. The reaction mixture was allowed to warm to room temperature followed by stirring for 18 h. To the solution, 20 mL of a 0.1 M HCl aqueous solution was added and the mixture was stirring for another 1 h. The resulting dark green solution was diluted with 15 mL of water and washed with toluene (20 mL × 2). The toluene solution was extracted with 15 mL of 0.1 M HCl. After addition of brine (10 mL) to the corrected aqueous solutions, the resulting aqueous layer was extracted with CH_2Cl_2 (20 mL × 4). The organic layer was washed with brine, dried over Na_2SO_4, and concentrated. The mixture was purified by reversed phase HPLC eluted with a gradient of CH_3CN/H_2O containing 0.1% TFA to afford 62.1 mg (59 μmol, 20%) of **8** as a TFA salt. ^{1}H NMR (CD_3OD, 400 MHz) δ 7.76 (dd, J = 12.6, 8.2 Hz, 2H), 7.61–7.54 (m, 4H), 7.27 (dd, J = 9.8, 6.2 Hz, 2H),

6.98–6.94 (m, 4H), 3.83 (s, 3H), 3.81 (s, 3H), 3.79–3.68 (m, 12H), 1.34–1.25 (m, 18H), 1.14–1.10 (m, 21H), ^{13}C NMR (CD_3OD, 125 MHz) δ 160.4 (s, C), 160.1 (s, C), 155.1 (d, J = 13.3 Hz, C), 144.4 (brs, C), 141.02 (s, CH), 140.96 (s, CH), 138.8 (d, J = 94.8 Hz, C), 133.8 (d, J = 109.2 Hz, C), 133.6 (d, J = 13.1 Hz, CH), 131.0 (d, J = 10.9 Hz, CH), 129.4 (d, J =2.4 Hz, C), 124.9 (d, J = 6.0 Hz, CH), 120.3 (d, J = 7.3 Hz, CH), 117.0 (s, CH), 112.6 (brs, C), 106.9 (s, C), 97.6 (brs, C), 95.7 (s, C), 57.4 (s, CH_3), 57.1 (s, CH_3), 52.9 (s, CH_2), 47.6 (s, CH_2), 19.0 (s, CH_3), 13.1 (s, CH_3), 12.4 (s, CH_3), 11.3 (s, CH). One C peak was overlapped. ^{31}P NMR (CD_3OD, 162 MHz) δ 8.9. HRMS (ESI) *m/z* calcd. for $C_{50}H_{69}N_3O_3PSi$ $[M]^+$: 818.4840; found: 818.4837

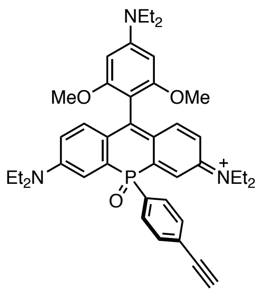

Et2NPOR-CCH

Et_2NPOR-CCH. This compound was synthesised according to the procedure for **POR-CCH**. The compound was purified by recrystallisation from a mixture of CH_2Cl_2/Et_2O at room temperature, followed by reversed phase HPLC eluted with a gradient of CH_3CN/H_2O containing 0.1% TFA to give the target compound as a TFA salt in 87% yield. ^{1}H NMR (CD_3OD, 400 MHz) δ 7.76 (dd, J = 12.2, 8.6 Hz, 2H), 7.60 (dd, J = 8.6, 3.1 Hz, 2H), 7.52 (dd, J = 16.2, 2.8 Hz, 2H), 7.42 (dd, J = 9.5, 6.4 Hz, 2H), 6.95 (dd, J = 9.5, 2.8 Hz, 2H), 6.30 (brs, 2H), 3.77–3.67 (m, 15H), 3.59 (q, J = 7.1 Hz, 4H), 1.32–1.25 (m, 18H). ^{13}C NMR (CD_3OD, 100 MHz) δ 160.4 (s, C), 160.0 (s, C), 159.7 (d, J = 6.7 Hz, C), 155.1 (d, J = 13.5 Hz, C), 143.5 (s, C), 140.9 (d, J = 8.7 Hz, CH), 138.9 (d, J = 95.4 Hz, C), 134.0 (d, J = 108.9 Hz, C), 133.8 (d, J = 13.5 Hz, CH), 130.9 (d, J = 10.6 Hz, CH), 128.7 (d, J = 2.9 Hz, C), 124.7 (d, J = 5.8 Hz, C), 120.4 (d, J = 6.7 Hz, CH), 117.1 (s, CH), 113.8 (s, C), 99.1 (brs, C), 83.0 (s, CH), 82.6 (s, CH), 57.5 (s, CH_3), 57.3 (s, CH_3), 53.7 (s, CH_2), 47.6 (s, CH_2), 13.1 (s, CH_3), 11.1 (s, CH_3). One CH peak was overlapped. ^{31}P NMR (CD_3OD, 162 MHz) δ 8.9. HRMS (ESI) *m/z* calcd. for $C_{41}H_{49}N_3O_3P$ $[M]^+$: 662.3506; found: 662.3507.

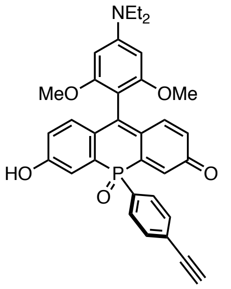

Et2NPOF-CCH

Et_2NPOF-CCH. This compound was synthesised according to the procedure for **POF-CCH**. The compound was purified by reversed phase HPLC eluted with a gradient of CH_3CN/H_2O containing 0.1% TFA in 84% yield (as a TFA salt). ^{1}H NMR (CD_3OD, 400 MHz) δ 7.71 (dd, J = 12.2, 8.4 Hz, 2H), 7.61 (dd, J = 8.4, 2.8 Hz, 2H), 7.22 (dd, J = 15.6, 2.2 Hz, 2H), 7.13 (dd, J = 9.4, 6.7 Hz, 2H), 6.74 (brs, 2H), 6.63 (dd, J = 9.4, 2.2 Hz, 2H), 3.80 (s, 6H), 3.76–3.68 (m, 5H), 1.31 (t, J = 7.4 Hz, 6H). ^{13}C NMR (CD_2Cl_2/CD_3OD = 9/1, 125 MHz) δ 173.1 (brs, C), 159.2 (s,

C), 149.3 (brs, C), 145.2 (s, C), 138.2 (d, J= 8.5 Hz, CH), 135.8 (d, J = 95.4 Hz, C), 133.8 (d, J = 108.6 Hz, C) 132.8 (d, J = 13.3 Hz, CH), 130.4 (d, J = 9.7 Hz, CH), 127.7 (d, J = 6.0 Hz, C), 127.0 (brs, CH), 126.8 (s, C), 124.6 (s, CH), 109.2 (brs, C), 94.5 (s, C), 82.6 (brs, CH), 80.6 (s, CH), 56.6 (s, CH3), 50.3 (s, CH2), 11.6 (s, CH3). One C peak and one CH peak were overwrapped. ^{31}P NMR (CD_3OD, 162 MHz) δ 8.0. HRMS (ESI) m/z calcd. for $C_{33}H_{30}NO_5PNa$ $[M+Na]^+$: 574.1759; found: 574.1754.

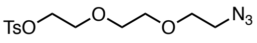

9

2-[2-(2-Azidoethoxy)ethoxy]ethyl *p*-toluenesulfonate (9). The compound was synthesised according to reported procedures.[5] Briefly, a mixture of NaN_3 (3.14 g, 48.2 mmol), 2-[2-(2-chloroethoxy)ethoxy]ethanol (2.91 mL, 3.38 g, 20.0 mmol), and KI (502 mg, 3.02 mmol) in anhydrous CH_3CN (20 mL) was refluxed for 60 h under a nitrogen atmosphere. After confirming the consumption of the starting material by ^{1}H NMR spectroscopy, the reaction mixture was cooled to room temperature. White precipitates were filtered off and the filtrate was concentrated. Et_2O (100 mL) was added and insoluble materials were filtered off through a pad of Celite. After removal of the solvent, the resulting colourless liquid was dissolved in anhydrous CH_3CN (7.5 mL), which was added to a solution of *p*-toluene sulfonyl chloride (4.51 g, 23.7 mmol) and triethylamine (5.0 mL, 36.0 mmol) in anhydrous CH_3CN (15 mL) at 0 °C. The reaction mixture was warmed up to room temperature followed by stirring for 2 h. To the solution, a 0.1 M HCl aqueous solution (20 mL) was added and the aqueous layer was extracted with ethyl acetate (30 mL × 3). The combined organic layer was successively washed with a 0.1 M HCl aqueous solution (20 mL), a 0.1 M NaOH aqueous solution (20 mL), water, and brine. After drying over Na_2SO_4, filtration, and concentration, the mixture was purified by silica-gel column chromatography eluted with hexane/ethyl acetate by a stepwise increase in the volume of ethyl acetate (9:1 to 3:1, v/v) to yield **9** (5.95 g, 18.1 mmol, 90%) as a colourless liquid. ^{1}H NMR (400 MHz, $CDCl_3$): δ 7.79 (d, J = 8.4 Hz, 2H), 7.34 (d, J = 8.4 Hz, 2H), 4.16 (t, J = 4.8 Hz, 2H), 3.69 (t, J = 4.8 Hz, 2H), 3.63 (t, J = 4.8 Hz, 2H), 3.60 (s, 4H), 3.36 (t, J = 4.8 Hz, 2H), 2.44 (s, 3H). ^{13}C NMR (100 MHz, $CDCl_3$): δ 144.9 (s, C), 133.1 (s, C), 129.9 (s, CH), 128.1 (s, CH), 70.9 (s, CH_2), 70.7 (s, CH_2), 70.2 (s, CH_2), 69.4 (s, CH_2), 68.9 (s, CH_2), 50.8 (s, CH_2), 21.8 (s, CH_3). HRMS (ESI): *m/z* calcd. for $C_{13}H_{19}N_3O_5SNa$ $[M+Na]^+$: 352.0943; found: 352.0932.

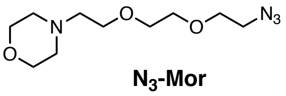

N_3-Mor

N_3-Mor. A suspension of **9** (359 mg, 1.09 mmol), morpholine (292 mg, 3.35 mmol), and K_2CO_3 (453 mg, 3.28 mmol) in anhydrous CH_3CN (5.0 mL) was refluxed for 12 h under a nitrogen atmosphere. After all the volatiles were removed under reduced pressure, the mixture was dissolved in toluene (20 mL) and extracted with a 0.1 M HCl aqueous solution (15 mL × 3). The combined aqueous layer was basified with a 2 M NaOH aqueous solution (5.0 mL) and extracted with CH_2Cl_2 (20 mL × 4). The organic layer was washed with brine, dried over anhydrous Na_2SO_4, and concentrated. The resulting colourless liquid of **N_3-Mor** (250 mg, 1.03 mmol, 94%) was used for next reaction without further purification. ^{1}H NMR ($CDCl_3$, 500 MHz) δ 3.72 (brs, 4H), 3.68–3.59 (m, 8H), 3.38 (t, J = 5.0 Hz, 2H), 2.61 (brs, 2H), 2.52 (brs, 4H). ^{13}C NMR ($CDCl_3$, 125 MHz) δ 70.8 (s, CH_2), 70.6 (s, CH_2), 70.2 (s,

CH_2), 68.9 (s, CH_2), 66.9 (s, CH_2), 58.3 (s, CH_2), 54.2 (s, CH_2), 50.8 (s, CH_2). HRMS (ESI) *m/z* calcd. for $C_{10}H_{20}N_4O_3Na$ $[M+Na]^+$: 267.1433; found: 267.1433.

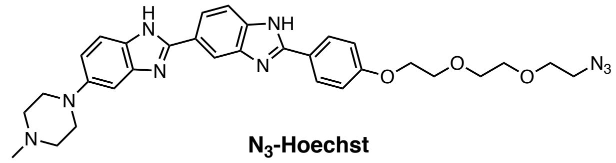

N_3-Hoechst

N_3-Hoechst. A suspension of Hoechst 33258·3HCl (50.7 mg, 95.0 μmol), **9** (66.4 mg, 202 μmol), and K_2CO_3 (80.5 mg, 582 μmol) in anhydrous CH_3CN/DMF (0.9 mL/2.0 mL) was stirred at 60 °C for 48 h. The inorganic salts were filtered off and the filtrate was concentrated under reduced pressure. The mixture was purified by silica-gel chromatography eluted with CH_2Cl_2/MeOH (199/1, v/v) containing 1% triethylamine, followed by reversed phase HPLC eluted with a gradient of CH_3CN/H_2O containing 0.1% TFA to afford a yellow viscous oil of **N_3-Hoechst** as a 3TFA salt (19.6 mg, 21.2 μmol, 23%). ^1H NMR (CD_3OD, 600 MHz) δ 8.33 (s, 1H), 8.07 (d, J = 6.9 Hz, 2H), 8.00 (d, J = 8.4 Hz, 1H), 7.86 (d, J = 8.4 Hz, 1H), 7.68 (d, J = 9.0 Hz, 1H), 7.35 (d, J = 9.0 Hz, 1H), 7.27 (s, 1H), 7.15 (d, J = 6.9 Hz, 2H), 4.23 (brs, 2H), 4.01–3.81 (m, 4H), 3.77–3.73 (m, 2H), 3.72–3.58 (m, 6H), 3.43–3.32 (m, 4H), 3.21 (brs, 2H), 3.01 (s, 3H). ^{13}C NMR (CD_3OD, 150 MHz) δ 163.6 (s, C), 155.9 (s, C), 150.8 (s, C), 150.5 (s, C), 141.0 (s, C), 139.2 (s, C), 134.9 (s, C), 130.3 (s, CH), 128.6 (s, C), 123.9 (s, CH), 120.3 (s, C), 119.7 (s, C), 119.3 (s, CH), 116.6 (s, CH), 116.5 (s, CH), 115.6 (s, CH), 115.5 (s, CH), 101.2 (s, CH), 71.9 (s, CH_2), 71.6 (s, CH_2), 71.2 (s, CH_2), 70.7 (s, CH_2), 69.1 (s, CH_2), 54.7 (s, CH_2), 51.8 (s, CH_2), 49.6 (s, CH_2), 43.6 (s, CH_3). HRMS (ESI) *m/z* calcd. for $C_{31}H_{36}N_9O_3$ $[M+H]^+$: 582.2941; found: 582.2937.

General procedure for the CuAAC reaction

Except for POX-SO_3H, a solution of POX-CCH, an azide compound (N_3-TPP,[6] N_3-Mor, or N_3-Hoechst), $Cu(CH_3CN)_4PF_6$, and tris[(1-benzyl-*1H*-1,2,3-triazol-4-yl)methyl]amine (TBTA) in anhydrous CH_3OH was stirred at room temperature for 21 h under a nitrogen atmosphere. For POX-SO_3H, a solution of POX-CCH, 3-azidopropane-1-sulfonic acid,[7] $CuSO_4$, tris[(hydroxypropyl-*1H*-1,2,3-triazol-4-yl)methyl]amine (THPTA), and sodium ascorbate in DMSO/PBS (1:1, v/v) was stirred at room temperature for 1 h. After all the volatiles were removed under reduced pressure, the mixture was dissolved in CH_3CN/water (1:1, v/v) containing 0.03% NH_3 (for PORL-SO_3H) or 0.1% TFA (for the others) and filtered through Presep® (Wako, ODS). The filtrate was purified by reversed phase HPLC eluted with a gradient of CH_3CN/H_2O containing 0.03% NH_3 (for PORL-SO_3H) or 0.1% TFA (for the others) to yield the target compounds.

POF-SO_3H. POF-CCH (4.0 mg, 8.3 μmol), azidopropane-1-sulfonic acid (4.7 mg, 25 μmol), $CuSO_4$ (0.66 mg, 4.1 μmol), and THPTA (3.6 mg, 8.3 μmol), sodium ascorbate (8.1 mg, 41 μmol), DMSO/PBS (2 mL/2 mL). 2.7 mg (48% yield) as a red/orange solid. ^1H NMR (400 MHz, CD_3OD): δ 8.56 (s, 1H), 8.00 (dd, J = 8.4, 2.8 Hz, 2H), 7.84 (dd, J = 12.8, 8.4 Hz, 2H), 7.57 (t, J = 8.6 Hz, 1H), 7.27 (dd, J = 15.6, 2.2 Hz, 2H), 7.15 (dd, J = 9.3, 6.7 Hz, 2H), 6.89 (dd, J = 8.6, 3.1 Hz, 2H), 6.62 (dd, J = 9.3, 2.2 Hz, 2H), 4.67 (t, J = 7.0 Hz, 2H), 3.79 (s, 3H), 3.77 (s, 3H), 2.85 (t, J = 7.0 Hz, 2H), 2.45–2.38 (m, 2H) ^{31}P NMR (162 MHz, CD_3OD): δ 8.2. HRMS (ESI): *m/z* calcd. for

$C_{32}H_{27}N_3O_8PS$ [M–H]$^-$: 644.1262; found: 644.1256. A satisfactory ^{13}C NMR spectrum was not obtained due to a small amount of product and the splitting of carbon signals.

PORL-SO$_3$H. PORL-CCH (4.3 mg, 8.0 µmol), azidopropane-1-sulfonic acid (4.5 mg, 24 µmol), $CuSO_4$ (0.64 mg, 4.0 µmol), and THPTA (3.5 mg, 8.0 µmol), sodium ascorbate (7.9 mg, 40 µmol), DMSO/PBS (2 mL/2 mL). 3.0 mg (52% yield) as a dark blue solid. ^1H NMR (500 MHz, CD_3OD): δ 8.50 (s, 1H), 7.98 (dd, J = 8.4, 2.3 Hz, 2H), 7.85 (dd, J = 12.3, 8.4 Hz, 2H), 7.55 (t, J = 8.4 Hz, 1H), 7.33 (dd, J = 15.7, 2.7 Hz, 1H), 7.14-7.04 (m, 3H), 6.87 (d, J = 8.4 Hz, 2H), 6.77 (dd, J = 9.2, 3.1 Hz, 1H), 6.29 (dd, J = 10.0, 2.3 Hz, 1H), 4.64 (t, J = 7.1 Hz, 2H), 3.80 (s, 3H), 3.76 (s, 3H), 3.53 (q, J = 7.2 Hz, 4H), 2.83 (t, J = 7.1 Hz, 2H), 2.43-2.37 (m, 2H), 1.18 (t, J = 7.2 Hz, 6H). ^{31}P NMR (202 MHz, CD_3OD): δ 9.5. HRMS (ESI): *m/z* calcd. for $C_{36}H_{36}N_4O_7PS$ [M–H]$^-$: 699.2048; found: 699.2042. A satisfactory ^{13}C NMR spectrum was not obtained due to a small amount of product and the splitting of carbon signals.

POR-SO$_3$H. POR-CCH (4.9 mg, 7.8 µmol), azidopropane-1-sulfonic acid (4.3 mg, 23 µmol), $CuSO_4$ (0.62 mg, 3.9 µmol), and THPTA (3.2 mg, 7.4 µmol), sodium ascorbate (7.6 mg, 38 µmol), DMSO/PBS (2 mL/2 mL). 2.5 mg (42% yield) as a green solid. ^1H NMR (400 MHz, CD_3OD): δ 8.50 (s, 1H), 8.00 (dd, J = 8.4, 3.1 Hz, 2H), 7.87 (dd, J = 12.5, 8.4 Hz, 2H), 7.62–7.57 (m, 3H), 7.28 (dd, J = 9.8, 6.1 Hz, 2H), 6.94–6.90 (m, 4H), 4.64 (t, J = 7.0 Hz, 2H), 3.81 (s, 3H), 3.75–3.70 (m, 11H), 2.80 (t, J = 7.0 Hz, 2H), 2.40 (t, J = 7.0 Hz, 2H), 1.27 (t, J = 7.3 Hz, 12H). ^{31}P NMR (202 MHz, CD_3OD): δ 9.3. HRMS (ESI): *m/z* calcd. for $C_{40}H_{47}N_5O_6PS$ [M]$^+$: 756.2979; found: 756.2984. A satisfactory ^{13}C NMR spectrum was not obtained due to a small amount of product and the splitting of carbon signals.

POF-TPP. POF-CCH (2.2 mg, 4.6 µmol), N_3-TPP (6.2 mg, 15 µmol), $Cu(CH_3CN)_4PF_6$ (1.2 mg, 3.2 µmol), and TBTA (2.7 mg, 5.1 µmol), anhydrous CH_3OH (2 mL). 1.2 mg (28% yield) as a red/orange powder. ^1H NMR (400 MHz, CD_3OD): δ 8.43 (s, 1H), 7.97 (dd, J = 8.4, 2.8 Hz, 2H), 7.89–7.70 (m, 17H), 7.57 (t, J = 8.2 Hz, 1H), 7,23 (brd, J = 15.6 Hz, 2H), 7.12 (dd, J = 9.6, 6.4 Hz, 2H), 6.88 (t, J = 8.2 Hz, 2H), 6.61 (brs, 2H), 4.65 (t, J = 6.4 Hz, 2H), 3.78 (s, 3H), 3.77 (s, 3H), 3.53–3.46 (m, 2H), 2.39–2.30 (m, 2H). ^{31}P NMR (162 MHz, CD_3OD): δ 23.0, 8.3. HRMS (ESI): *m/z* calcd. for $C_{50}H_{42}N_3O_5P_2$ [M]$^+$: 826.2594; found: 826.2591. A satisfactory ^{13}C NMR spectrum was not obtained due to a small amount of product and the splitting of carbon signals.

PORL-Mor. PORL-CCH (2.6 mg, 4.9 µmol), N_3-Mor (11.7 mg, 47.9 µmol), $Cu(CH_3CN)_4PF_6$ (2.1 mg, 5.6 µmol), and TBTA (2.6 mg, 49 µmol), anhydrous CH_3OH (2 mL). 1.8 mg (41% yield) as a dark blue powder. ^1H NMR (400 MHz, CD_3OD): δ 8.48 (s, 1H), 7.99 (dd, J = 8.4, 3.2 Hz, 2H), 7.87 (dd, J = 12.4, 8.4 Hz, 2H), 7.55 (t, J = 8.2 Hz, 1H), 7.33 (dd, J = 16.0, 2.4 Hz, 1H), 7.16–7.03 (m, 3H), 6.87 (dd, J = 8.6, 1.8 Hz, 2H), 6.77 (dd, J = 9.2, 3.2 Hz, 1H), 6.28 (dd, J = 10.2, 2.2 Hz, 1H), 4.62 (t, J = 5.2 Hz, 2H), 3.91 (t, J = 5.2 Hz, 2H), 3.81 (s, 3H), 3.76 (s, 3H), 3.63–3.58 (m, 2H), 3.57–3.45 (m, 12H), 2.38 (t, J = 5.4 Hz, 2H), 2.31 (t, J = 4.6 Hz, 4H), 1.19 (t, J = 7.0 Hz, 6H). ^{13}C NMR (CD_3OD, 100 MHz) δ 185.3 (d, J = 13.5 Hz, C), 159.2 (s, C), 158.9 (s, C), 155.4 (d, J = 6.7 Hz, C), 152.0 (d, J = 12.4 Hz, C), 147.2 (s, C), 142.4 (d, J = 9.6 Hz, CH), 141.0 (d, J = 92.0 Hz, C), 137.4 (d, J = 10.6 Hz, CH),

136.1 (d, J = 1.9 Hz, C), 134.7 (d, J = 96.8 Hz, C), 134.2 (d, J = 108.3 Hz, C), 132.8 (d, J = 2.9 Hz, CH), 132.6 (s, CH), 131.7 (d, J = 11.5 Hz, CH), 127.3 (s, CH), 127.1 (d, J = 12.5 Hz, CH), 124.5 (d, J = 6.7 Hz, C), 124.43 (s, C), 124.39 (s, CH), 116.3 (d, J = 7.7 Hz, CH), 115.7 (s, CH), 114.8 (s, C), 105.2 (s, CH), 105.1 (s, CH), 71.5 (s, CH_2), 71.2 (s, CH_2), 70.2 (s, CH_2), 69.3 (s, CH_2), 67.4 (s, CH_2), 59.2 (s, CH_2), 56.8 (s, CH_3), 56.5 (s, CH_3), 55.0 (s, CH_2), 51.7 (s, CH_2), 46.1 (s, CH_2), 12.8 (s, CH_3). ^{31}P NMR (CD_3OD, 162 MHz): δ 9.4. HRMS (ESI): m/z calcd. for $C_{43}H_{51}N_5O_7P$ $[M+H]^+$: 780.3526; found: 780.3549.

POR-Hoechst. POR-CCH (3.6 mg, 5.7 µmol), N_3-Hoechst (12.0 mg, 13.0 µmol), $Cu(CH_3CN)_4PF_6$ (2.4 mg, 6.4 µmol), and TBTA (4.3 mg, 8.1 µmol), DIEA (0.10 mL, 0.57 mmol), anhydrous CH_3OH (2 mL). 4.2 mg (44% yield) as a green powder. ^{1}H NMR (400 MHz, CD_3OD): δ 8.54 (s, 1H), 8.40 (s, 1H), 8.08–7.99 (m, 5H), 7.90–7.85 (m, 3H), 7.71 (d, J = 9.2 Hz, 1H), 7.60–7.52 (m, 3H), 7.37 (dd, J = 9.2, 1.8 Hz, 1H), 7.33 (s, 1H), 7.27 (dd, J = 9.8, 6.1 Hz, 2H), 7.03 (d, J = 9.2 Hz, 2H), 6.92 (dd, J = 9.8, 2.4 Hz, 2H), 6.86 (dd, J = 7.9, 6.7 Hz, 2H), 4.64 (t, J = 5.2 Hz, 2H), 4.12 (t, J = 4.6 Hz, 2H), 3.95 (t, J = 4.9 Hz, 2H), 3.80–3.67 (m, 26H), 3.35 (s, 2H), 3.01 (s, 3H), 1.24 (t, J = 7.0 Hz, 12H). $^{31}P\{^1H\}$ NMR (162 MHz, CD_3OD): δ 9.4. HRMS (ESI): m/z calcd. for $C_{68}H_{75}N_{11}O_6P$ $[M]^+$: 1172.5634; found: 1172.5645. A satisfactory ^{13}C NMR spectrum was not obtained due to the small amount of product and the splitting of carbon signals.

1.2. Photophysical properties

UV-vis absorption spectra were measured with a Shimadzu UV-3150 spectrometer, an Agilent 8454 UV-visible spectrophotometer, or HORIBA Duetta fluorescence and absorbance spectrometer using a 1 cm square quartz cuvette. Emission spectra were measured with a HORIBA FluoroMax-4 spectrometer, a HORIBA Fluorolog-3 VIS/NIR fluorescence spectrophotometer, or HORIBA Duetta fluorescence and absorbance spectrometer. Absolute fluorescence quantum yields (Φ_F) were determined using a HAMAMATSU Quantaurus-QY (C11347-11 or C13534) spectrometer equipped with a calibrated integrating sphere system.

Table S1. Photophysical Data for POX and POX-SO_3H Dyes.[a]

	λ_{abs} (nm)	ε (M^{-1} cm^{-1})	λ_{em} (nm)	Φ_F
**POF**[b]	632	54,500	661	0.24
**POF-SO_3H**	632	33,700	662	0.24
**PORL**[b]	670	66,100	698	0.11
**PORL-SO_3H**	670	54,000	699	0.12
**POR**[b]	712	94,200	740	0.13
**POR-SO_3H**	713	95,300	739	0.12

[a] Measured in 50 mM HEPES buffer (pH 7.4) containing 0.1% DMSO as a co-solvent.
[b] The data are cited from references 4 and 9.

1.3. Theoretical calculations

The geometry optimisations for **POR** and **POR-triazole** were carried out using density functional theory (DFT) at the level of M06-2X[10]/6-31+G[11] in aqueous phase using the SMD solvation model,[12] implemented in the Gaussian 16 Revision B.01 software package.[13] All stationary points were optimised without any symmetry assumptions and characterised by frequency analysis at the same level of theory (the number of imaginary frequencies, NIMAG, was 0). The cartesian coordinates of the optimised geometries are given in Tables S2 and S3. The excitation energies for the optimised structures were calculated based on TD-DFT at the M06-2X/6-31+G(d) level of theory, using the SMD solvent model in water. The calculation results are shown in Fig. S4.

1.4. Evaluation of the photostability

A PBS solution of POX-SO_3H, Alexa Fluor 700 (Thermo Fisher Scientific), or Cy5.5 (GE Healthcare) was prepared to give the absorption maximum of ~0.1. The samples were then irradiated with 300 W xenon lamp (MAX-302, Asahi Spectra) using continuous range of visible light 385–740 nm (light power: 400 mW/cm^2). Changes of absorption and fluorescence spectra over time were monitored.

1.5. Determination of acid dissociation constant (pK_a)

Spectroscopic determination of acid dissociation constants (pK_a) is shown below. A series of pH-buffered solutions was prepared with 0.1 M citric acid/0.1 M sodium citrate for pH 3.1–5.7 and 0.1 M Na_2HPO_4/0.1 M NaH_2PO_4 for pH 5.8–7.9. For sample preparation, a stock solution of Et_2NPOF in DMSO was added to 2 mL of each pH solution (2 µL from 10 mM stock and 1 µL from 2.5 mM stock for the absorption and fluorescence measurements, respectively).

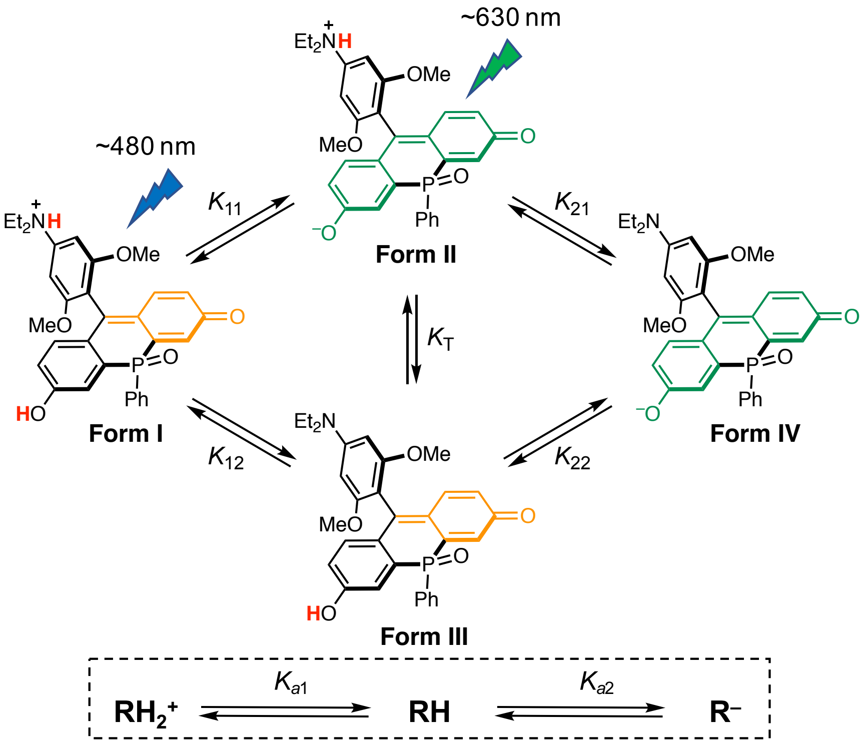

Scheme S1. Macroscopic and microscopic protonation equilibria for Et_2NPOF.

Protonation equilibrium constants of Et_2NPOF in Scheme S1 can be described as follows; K_{11} = [**II**][H]/[**I**], K_{12} = [**III**][H]/[**I**], K_{21} = [**IV**][H]/[**II**], and K_{22} = [**IV**][H]/[**III**], where [**I**]-[**IV**] mean the concentration of each protonated form. An equilibrium constant for tautomerisation (K_T) is K_T = [**II**]/[**III**], thus $K_T = K_{11}/K_{12} = K_{22}/K_{21}$. Overall, macroscopic protonation equilibria are defined by K_{a1} = [RH][H]/[RH_2^+] and K_{a2} = [R^-][H]/[RH], in which RH_2^+, RH, and R^- represent cation (Form I), neutral species (Form II and Form III), and anion (Form IV), respectively. Thus, K_{a1} and K_{a2} are expressed by $K_{a1} = K_{11} + K_{12}$ and $1/K_{a2} = 1/K_{21} + 1/K_{22}$, respectively.

UV-vis spectroscopic titration: Because of the presence of a distinct isosbestic point in the UV-vis spectroscopic titration, the spectral features of Form I and Form III as well as Form II and Form IV should be comparable to each other. Thus, the absorbance (A) is defined by

$$A = \varepsilon_{OH}[\mathbf{I}] + \varepsilon_{O^-}[\mathbf{II}] + \varepsilon_{OH}[\mathbf{III}] + \varepsilon_{O^-}[\mathbf{IV}] \quad \text{(Eq. 1)}$$

where ε_{OH} and ε_{O^-} indicate the absorption coefficients of protonated (Form I and Form III) and deprotonated (Form II and Form IV) forms of the OH group, respectively. Due to the negligible values of ε_{OH} beyond 600 nm, the absorbance at 630 nm (A_{630}) can be simplified as

$$A_{630} = \varepsilon_{O^-}[\mathbf{II}] + \varepsilon_{O^-}[\mathbf{IV}] \quad \text{(Eq. 2)}$$

The total concentration of Et_2NPOF ($[POF]_T$) in a solution is given in Eq. 3.

$$\begin{aligned}[POF]_T &= [\mathbf{I}] + [\mathbf{II}] + [\mathbf{III}] + [\mathbf{IV}] \\ &= [\mathbf{II}][H]/K_{11} + [\mathbf{II}] + K_{21}[\mathbf{II}]/K_{22} + K_{21}[\mathbf{II}]/[H] \\ &= \frac{[H]^2 + K_{11}[H] + K_{12}[H] + K_{11}K_{21}}{K_{11}[H]}[\mathbf{II}] \quad \text{(Eq. 3)}\end{aligned}$$

Thus, Eq. 2 is

$$\begin{aligned}A_{630} &= \varepsilon_{O^-}[\mathbf{II}] + \varepsilon_{O^-} K_{21}[\mathbf{II}]/[H] \\ &= (1 + K_{21}/[H])\varepsilon_{O^-}[\mathbf{II}] \\ &= \frac{K_{11}[H] + K_{11}K_{21}}{[H]^2 + K_{11}[H] + K_{12}[H] + K_{11}K_{21}}\varepsilon_{O^-}[POF]_T \\ &= \frac{K_{11}[H] + K_{11}K_{21}}{[H]^2 + K_{11}[H] + K_{12}[H] + K_{11}K_{21}}A_{O^-} \\ &= \frac{10^{-(pK_{11}+pH)} + 10^{-(pK_{11}+pK_{21})}}{10^{-2pH} + 10^{-(pK_{11}+pH)} + 10^{-(pK_{12}+pH)} + 10^{-(pK_{11}+pK_{21})}}A_{O^-} \quad \text{(Eq. 4)}\end{aligned}$$

where A_{O^-} means the absorbance at a given wavelength when all Et_2NPOF are present as the deprotonated form, namely the absorbance at pH 8. The absorption at 630 nm was plotted as a function of pH and the data were fitted by nonlinear curve fitting with Eq. 4 (R = 0.99997) to calculate the protonation equilibrium constants (Fig. S5). The results are summarised in Table S4.

Fluorescence spectroscopic titration: Due to the enhanced acidity of the phenolic proton of POF dyes in the excited state, even upon excitation of the protonated forms at around 480 nm, it is observed as the emission spectrum of the deprotonated form with a maximum at λ_{em} = 664 nm. Therefore, in the following titration experiments, fluorescence intensities at 664 nm were plotted as a function of pH.

When excited at 633 nm, the overall fluorescence intensity is described as $I_{ex633} = c_2^{633}[II]$, where c_2^{633} represents the proportionality coefficient for Form II at λ_{ex} = 633 nm. Thus,

$$I_{ex633} = \frac{K_{11}[H]}{[H]^2 + K_{11}[H] + K_{12}[H] + K_{11}K_{21}} c_2^{633}[POF]_T$$

$$= \frac{K_{11}[H]}{[H]^2 + K_{11}[H] + K_{12}[H] + K_{11}K_{21}} I_{max(II)}^{633} \quad \text{(Eq. 5)}$$

Here, $I_{max(II)}^{633}$ is the maximum fluorescence intensity defined as $I_{max(II)}^{633} = c_2^{633}[POF]_T$, which cannot be obtained experimentally. Therefore, using the calculated protonation equilibrium constants in Table S4, $[II]/[POF]_T$ at pH 5.5 was calculated to be 0.189 from Eq. 3. The Eq. 5 is

$$I_{ex633} = \frac{K_{11}[H]}{[H]^2 + K_{11}[H] + K_{12}[H] + K_{11}K_{21}} c_2^{633}[II]/0.189$$

$$= \frac{K_{11}[H]}{[H]^2 + K_{11}[H] + K_{12}[H] + K_{11}K_{21}} I_{obs}^{633} \times 5.29 \quad \text{(Eq. 5')}$$

in which I_{obs}^{633} is the fluorescence intensity observed at pH 5.5. Using Eq. 5', the plots were fitted to give the equilibrium constants shown in Fig. S6 and Table S4.

Ratiometric fitting: Upon excitation at 488 nm and 534 nm, the observed fluorescence signals are the sum of Form I and Form II. In this case, the fluorescence intensities are described as $I_{ex488} = c_1^{488}[I] + c_2^{488}[II]$ and $I_{ex534} = c_1^{534}[I] + c_2^{534}[II]$ by using the corresponding proportionality coefficient for Form I and Form II at given excitation wavelengths.

$$I_{ex488} = \frac{c_1^{488}[POF]_T[H]^2 + c_2^{488}[POF]_T K_{11}[H]}{[H]^2 + K_{11}[H] + K_{12}[H] + K_{11}K_{21}} = \frac{I_{max(I)}^{488}[H]^2 + I_{max(II)}^{488}K_{11}[H]}{[H]^2 + K_{11}[H] + K_{12}[H] + K_{11}K_{21}} \quad \text{(Eq. 6)}$$

$$I_{ex534} = \frac{c_1^{534}[POF]_T[H]^2 + c_2^{534}[POF]_T K_{11}[H]}{[H]^2 + K_{11}[H] + K_{12}[H] + K_{11}K_{21}} = \frac{I_{max(I)}^{534}[H]^2 + I_{max(II)}^{534}K_{11}[H]}{[H]^2 + K_{11}[H] + K_{12}[H] + K_{11}K_{21}} \quad \text{(Eq. 7)}$$

Thus, fluorescence intensity ratio R can be described as

$$R = I_{ex534}/I_{ex488} = \frac{I_{max(I)}^{534}[H] + I_{max(II)}^{534}K_{11}}{I_{max(I)}^{488}[H] + I_{max(II)}^{488}K_{11}} \quad \text{(Eq. 8)}$$

Here, $I_{max(I)}^{488}$ and $I_{max(I)}^{534}$ can be determined as the fluorescence intensities at pH 3. Using a given K_{11} value, $I_{max(II)}^{488}$ and $I_{max(II)}^{534}$ are determined by non-linear curve fitting with Eq. 8 (Fig. S7). In principle, pH can be ratiometically calculated using R values by Eq. 9:

$$[H] = \frac{R - I_{max(II)}^{534} / I_{max(II)}^{488}}{I_{max(I)}^{534} / I_{max(I)}^{488} - R} \left(\frac{I_{max(II)}^{488}}{I_{max(I)}^{488}}\right) K_{11} = 0.117 \times \frac{R_{max} - R}{R - R_{min}} K_{11}$$

$$pH = pK_{11} - \log\frac{R_{max} - R}{R - R_{min}} + 0.93 \quad \text{(Eq. 9)}$$

1.6. Preparation of dye-conjugated dextran

Synthesis of azido-dextran (N_3-dex)

A solution of 10 kDa amino dextran (49.1 mg, ThermoFisher) in 3 mL of tris-HCl buffer (pH 8.6) was stirred at room temperature for 20 min, to which a solution of 8.3 mg of azido-PEG_3-NHS ester (BroadPharm) in DMSO (500 µL) was added. The solution was stirred at room temperature for 12 h. After concentration under reduced pressure, the mixture was suspended in CH_3OH. The white precipitate was collected by filtration, washed with CH_3OH twice, and dissolved in water. The solution was passed through membrane filter (0.45 µm) and the filtrate was lyophilised to yield N_3-Dex (49.0 mg) as a white solid.

Labelling of N_3-dex with fluorescent dyes (POF-dex and Et_2NPOF-dex)

To a solution of N_3-dex, $CuSO_4$, tris[(hydroxypropyl-*1H*-1,2,3-triazol-4-yl)methyl]amine (THPTA) and sodium ascorbate in PBS (pH 7.4) was added POF-CCH or Et_2NPOF-CCH·TFA salt in DMSO. After stirring for 1.5 h at room temperature, unreacted free dyes were removed by gel filtration chromatography using Sephadex™ G-25 Fine (GE Healthcare). After concentrated under reduced pressure, the residue was suspended in CH_3OH. The residual dark blue solid was collected, washed with CH_3OH, dissolved in Mill-Q water containing 0.1% TFA, and lyophilised to afford **POF-dex** (9.7 mg) or **Et_2NPOF-dex** (16.1 mg) as a red solid.

Determination of the degree of labelling (DOL)

Lyophilised POF-dex (9.7 mg) or Et_2NPOF-dex (16.1 mg) were dissolved in distilled water (300 µL and 200 µL, respectively) to give aqueous solutions of the dye-conjugated dextran (3.2 mM and 5.9 mM, respectively). The degree of labelling (DOL) was determined from the UV-vis absorption spectra to be 1.05 (POF-dex) and 1.06 (Et_2NPOF-dex) using Eq. 10:

$$\mathrm{DOL} = \frac{A_{632}}{\varepsilon_{\mathrm{O}^-} \cdot C_{\mathrm{dex}}} \qquad \text{(Eq. 10)}$$

where A_{632}, $\varepsilon_{\mathrm{O}^-}$, and C_{dex} mean the absorbance at 632 nm, the molar absorption coefficient of POF at pH 7.4 (54,500 M^{-1} cm^{-1}), and the concentration of dextran, respectively.

Absorption and emission spectra of Et_2NPOF-dex

UV-vis absorption and emission spectra of Et_2NPOF-dex solutions were measured using SpectraMax i3 (Molecular Devices). For the absorption measurement, a stock solution of the dye (6.2 mM) was diluted with the various pH-buffer solutions to reach a final concentration of 40 µM in a 96-well microplate. For the emission measurement, a stock solution of the dye was diluted with the various pH-buffer solutions to get a final concentration of 1 µM.

1.7. Cell experiments

Cell culture

HeLa (RCB0007) and A431 (RCB0202) cells were purchased from RIKEN Cell Bank. Dulbecco's modified Eagle's medium (DMEM) with low glucose, L-glutamine, sodium pyruvate (Wako, 041-29775), fetal bovine serum (FBS, Biosera, 554-02155), and Antibiotic-Antimycotic (AA, Wako, 161-23181) were used for culture. Cells were cultured in DMEM containing 10% FBS and 1% AA at 37 °C in a humidified 5% CO_2 incubator. Three days before imaging, A431 cells (3×10^4) were seeded in poly-lysine coated glass-bottom dishes and HeLa cells (5×10^4) were seeded in non-coated glass-bottom dishes.

Cell viability assays

HeLa cells were seeded into a flat-bottomed 96-well plate (0.7×10^4 cells/well) and incubated in DMEM containing 10% FBS at 37 °C in a 5% CO_2 incubator for 36 h. The medium was then replaced with a culture medium containing various concentrations of POF-TPP, PORL-Mor or POR-Hoechst with indicated concentrations in the medium. After the cells were incubated for indicated time at 37 °C, the medium was removed and the cells were washed with PBS three times. MTT reagent (final concentration, 0.2 mg/mL) in PBS was added to each well, and the plates were incubated for another 3 h in a CO_2 incubator. Excess MTT tetrazolium solution was then removed and the cells were once washed with PBS. After the formazan crystals were solubilised in DMSO (200 µL/well) for 30 min at room temperature, the absorbance of each well was measured by SpectraMax i3 (Molecular Devices) with an excitation at 535 nm.

Cell imaging

We used two confocal microscopies, FLUOVIEW FV10i (Olympus) equipped with four excitation lasers (405, 473, 559, and 635 nm) and FLUOVIEW FV3000 (Olympus) equipped with five excitation lasers (405, 488, 561, 640, and 730 nm), GaAsP PMT and GaAs PMT detectors, and TruFocus Red Z-Drift Compensator. The cell dish was mounted on a stage top incubator (Tokai Hit) maintained at 37 °C in an environment of humidified 5% CO_2. The images in multiple channels were recorded in a line-by-line.

Organelle labelling

Prior to staining cells with POF, the dye was acetylated (AcPOF) by treatment with acetic anhydride to allow permeabilisation.[8] After removal of the incubation medium from the culture dish, the cells were washed with DMEM three times. Typically, A431 cells were stained with each POX dye in DMEM containing 0.05 % Pluonic F-127 and 0.5% DMSO in a CO_2 incubator at the following concentrations and incubation times; POF-TPP: 5 µM for 3 h, PORL-Mor: 500 nM for 30 min, POF: 5 µM for 30 min, PORL: 500 nM for 30 min, POR: 500 nM for 30 min. For staining with POR-Hoechst, 500 nM of the dye was used and the cells were incubated in HBSS containing 25 mM HEPES, 0.05% Pluonic F-127, 0.1% NaN_3, and 0.5% DMSO for 30 min. For co-staining experiments, 50 nM LysoTracker Green®, 50 nM MitoTracker Green®, or 1 µM Hoechst 33342 were used. Acquisition conditions are indicated in each Figure caption.

Cell imaging with Et_2NPOF-dex

The incubation medium was removed from the culture dish and the cells were washed with DMEM three times. HeLa cells were incubated in DMEM containing 2 mg/mL of Et_2NPOF-dex in a CO_2 incubator for 24 h. The images were recorded using two different excitation wavelengths, 488 nm and 640 nm, with or without washing the cells. The fluorescence signal in the range of 650–750 nm was detected. After 24 h incubation with Et_2NPOF-dex, the cells were also stained with 50 nM of LysoTracker Green and the acidic compartments were imaged at λ_{ex} = 488 nm (λ_{em} = 500–600 nm). As a control, POF-dex was used instead of Et_2NPOF-dex.

2. Figures and tables

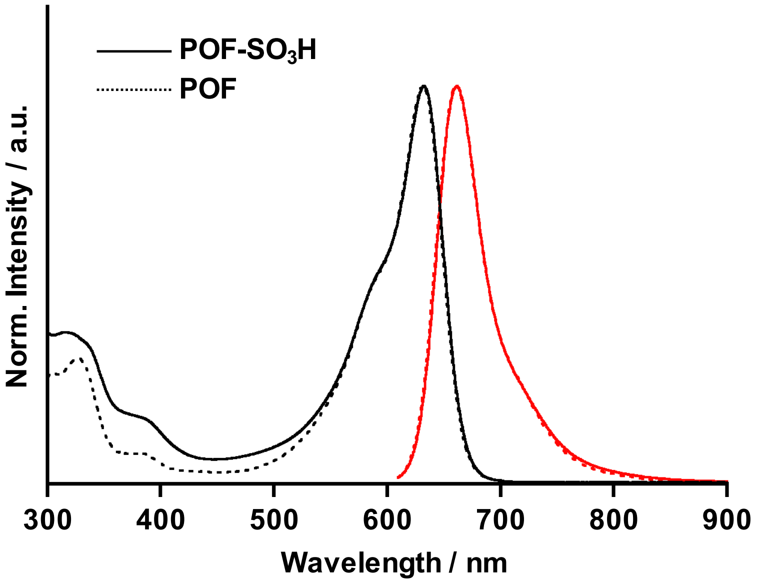

Fig. S1 Normalised absorption (black line) and emission (red line) spectra of POFs in 50 mM HEPES buffer (pH 7.4) containing 0.1% DMSO as a co-solvent. Solid and dashed lines represent the spectra of POF-SO_3H and POF, respectively.

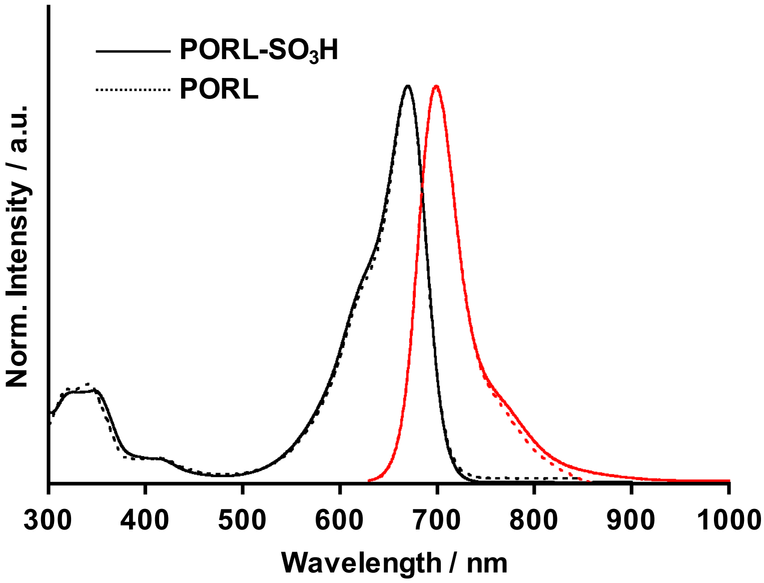

Fig. S2 Normalised absorption (black line) and emission (red line) spectra of PORLs in 50 mM HEPES buffer (pH 7.4) containing 0.1% DMSO as a co-solvent. Solid and dashed lines represent the spectra of PORL-SO_3H and PORL, respectively.

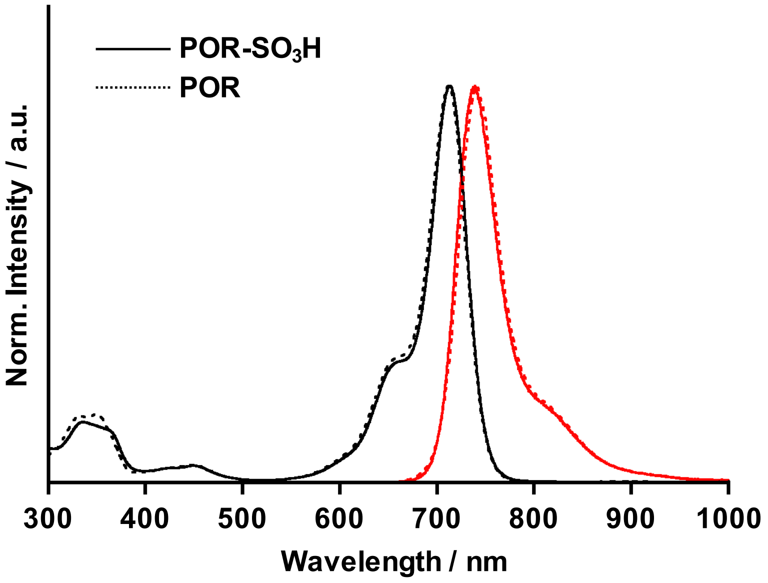

Fig. S3 Normalised absorption (black line) and emission (red line) spectra of PORs in 50 mM HEPES buffer (pH 7.4) containing 0.1% DMSO as a co-solvent. Solid and dashed lines represent the spectra of POR-SO_3H and POR, respectively.

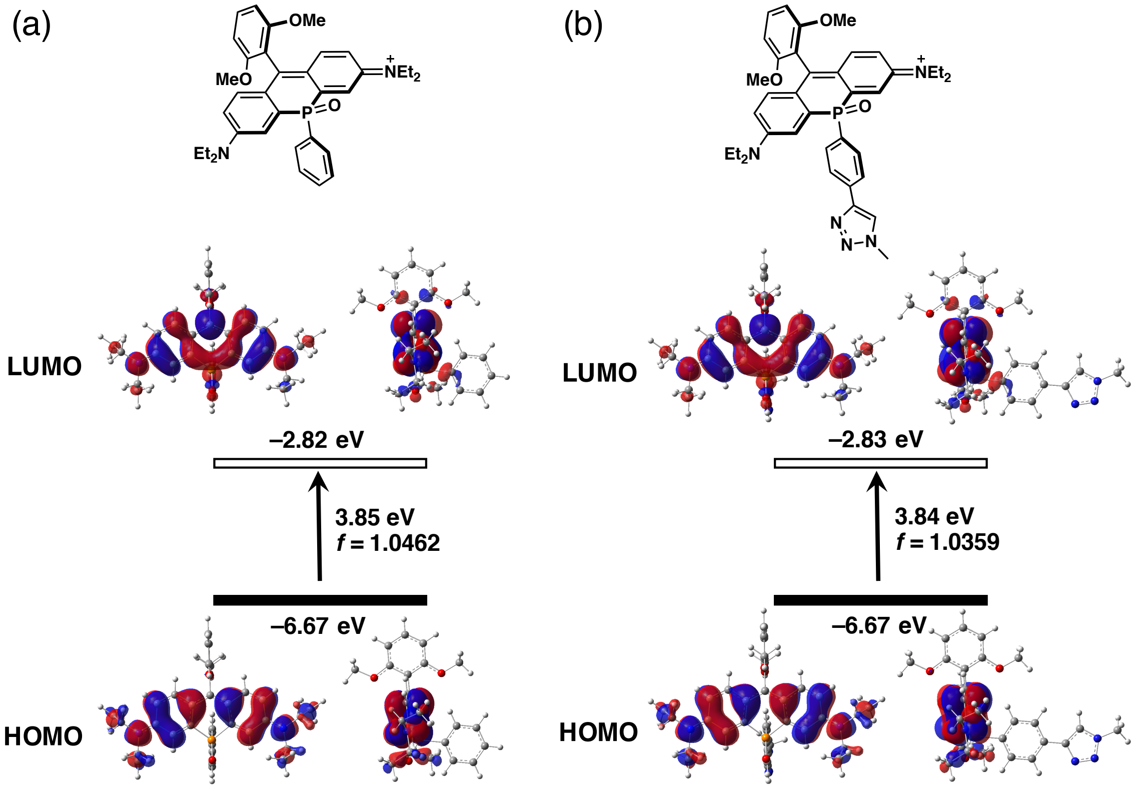

Fig. S4 Energy diagram, Kohn-Sham HOMOs and LUMOs, and the TD-DFT vertical excitation wavelengths and oscillator strengths (*f*) for the model compounds of (a) POR and (b) POR-triazole calculated at the M06-2X/6-31+G(d) level of theory with the SMD solvent model.

Table S2. Cartesian Coordinates (in Å) of POR

Atom	*x*	*y*	*z*	Atom	*x*	*y*	*z*
H	2.31613	2.60331	-0.01605	O	0.07346	1.99723	2.59260
C	2.39791	1.56121	-0.30423	O	-0.22696	3.38309	-1.89243
C	2.64160	-1.12336	-1.02827	C	0.11673	2.24837	4.02665
C	1.22031	0.75448	-0.32444	H	0.16374	1.26322	4.48609
C	3.63481	1.07528	-0.64577	H	-0.78676	2.77195	4.34840
C	3.80914	-0.28566	-1.05403	H	1.00640	2.82736	4.28515
C	1.42097	-0.61406	-0.67539	C	-0.35182	4.40615	-2.92219
H	4.48236	1.74484	-0.60267	H	-1.26933	4.98121	-2.77646
H	2.71763	-2.17477	-1.27920	H	-0.39548	3.86065	-3.86198
C	-0.05313	1.32637	-0.05140	H	0.51820	5.06697	-2.91033
P	0.02392	-1.79497	-0.56583	N	-5.08090	-1.10259	-0.85524
C	-1.45367	-0.71250	-0.50375	N	5.00532	-0.76733	-1.44675
C	-2.66903	-1.30202	-0.71789	C	5.19360	-2.19864	-1.76139
H	-2.70845	-2.35479	-0.97264	H	6.11628	-2.27527	-2.33818
C	-3.88475	-0.53943	-0.61645	H	4.38328	-2.52990	-2.41642
C	-3.75632	0.83771	-0.24270	C	5.28137	-3.07823	-0.51380
H	-4.63839	1.44822	-0.10663	H	4.38765	-2.98057	0.11042
C	-2.52505	1.40316	-0.03618	H	6.15345	-2.81186	0.08867
H	-2.47891	2.45047	0.24062	H	5.37610	-4.12633	-0.81284
C	-1.30505	0.67197	-0.18123	C	6.21404	0.08061	-1.42652
O	-0.00727	-2.90739	-1.75124	H	5.96214	1.06498	-1.82902
C	0.16186	-2.59874	1.08649	H	6.92768	-0.36965	-2.11807
C	0.38732	-3.82710	3.56711	C	6.83426	0.20106	-0.03442
C	0.16862	-1.81171	2.24289	H	7.68544	0.88744	-0.07106
C	0.26703	-3.98796	1.15315	H	7.19112	-0.77034	0.31739
C	0.38041	-4.60268	2.40474	H	6.11188	0.58837	0.69071
C	0.28183	-2.43309	3.48774	C	-6.33242	-0.32082	-0.84559
H	0.08788	-0.72918	2.17628	H	-6.14084	0.67468	-1.25084
H	0.26064	-4.58024	0.24403	H	-7.01701	-0.81827	-1.53734
H	0.46264	-5.68178	2.46788	C	-6.94859	-0.24406	0.54926
H	0.28762	-1.83290	4.39066	H	-7.88515	0.31942	0.51089
H	0.47529	-4.30677	4.53564	H	-6.27251	0.25447	1.25050
H	-0.13992	6.45820	1.51933	H	-7.16468	-1.24749	0.92875
C	-0.12224	5.42283	1.19862	C	-5.23913	-2.53966	-1.15358
C	-0.07601	2.75936	0.37217	H	-6.24427	-2.81617	-0.82555
C	-0.03244	4.41308	2.15882	H	-4.54056	-3.11866	-0.54613
C	-0.18986	5.13017	-0.16506	C	-5.06550	-2.83885	-2.64079
C	-0.16537	3.79203	-0.56609	H	-4.06039	-2.57307	-2.98228
C	-0.01111	3.08311	1.73186	H	-5.79374	-2.27567	-3.23251
H	0.01910	4.66374	3.21033	H	-5.22233	-3.90540	-2.82503
H	-0.25942	5.92993	-0.89069				

Table S3. Cartesian Coordinates (in Å) of POR-Triazole

Atom	*x*	*y*	*z*	Atom	*x*	*y*	*z*
H	-2.30212	2.97091	1.36870	H	0.01735	1.21334	4.77672
C	-1.69629	2.87199	0.47483	C	-6.12580	1.15261	0.49645
C	-0.11551	2.64532	-1.81451	H	-6.67515	0.36517	1.01787
C	-1.40270	1.56583	-0.02054	H	-6.35277	1.12749	-0.56687
C	-1.25352	4.01515	-0.14229	H	-6.37612	2.13161	0.91191
C	-0.46705	3.95427	-1.33669	N	-2.03487	-4.81816	-1.49939
C	-0.56729	1.52183	-1.17646	N	-0.07299	5.06395	-1.99258
H	-1.51410	4.96986	0.29295	C	0.82109	4.98881	-3.16616
H	0.52114	2.53587	-2.68447	H	0.72643	5.93878	-3.69396
C	-1.96493	0.41494	0.59874	H	0.45548	4.21338	-3.84491
P	0.02390	-0.09518	-1.80332	C	2.28261	4.74138	-2.79074
C	-1.12306	-1.30071	-1.03482	H	2.39890	3.81763	-2.21533
C	-1.15145	-2.56266	-1.56027	H	2.67656	5.56973	-2.19630
H	-0.55613	-2.78512	-2.43847	H	2.88303	4.65458	-3.70116
C	-1.97035	-3.58641	-0.96790	C	-0.39300	6.40947	-1.47698
C	-2.70109	-3.23109	0.21191	H	-1.43155	6.42000	-1.13692
H	-3.29383	-3.97405	0.72752	H	-0.33078	7.09540	-2.32320
C	-2.65325	-1.95790	0.71716	C	0.55123	6.86142	-0.36298
H	-3.22727	-1.73541	1.60973	H	0.22927	7.83578	0.01651
C	-1.89064	-0.91354	0.10691	H	1.57394	6.95877	-0.73621
O	0.11426	-0.19985	-3.42264	H	0.55245	6.15208	0.47035
C	1.67228	-0.37837	-1.03415	C	-2.94895	-5.85202	-0.97705
C	4.18042	-0.82002	0.11356	H	-3.87669	-5.38012	-0.64679
C	1.81667	-0.31585	0.35539	H	-3.20349	-6.49707	-1.82202
C	2.76506	-0.65697	-1.85412	C	-2.31206	-6.67034	0.14355
C	4.01721	-0.87647	-1.27850	H	-3.01017	-7.44102	0.48243
C	3.06749	-0.53696	0.92460	H	-2.05550	-6.03584	0.99722
H	0.96400	-0.09882	0.99441	H	-1.40080	-7.16212	-0.21035
H	2.64262	-0.70326	-2.93097	C	-1.21157	-5.23006	-2.65315
H	4.86898	-1.09440	-1.91155	H	-1.07029	-6.31020	-2.56454
H	3.17630	-0.48842	2.00294	H	-0.22152	-4.77629	-2.57457
H	-4.66389	1.18139	5.17295	C	-1.87672	-4.88912	-3.98447
C	-4.12292	1.02766	4.24611	H	-2.01986	-3.80934	-4.08951
C	-2.73348	0.63213	1.86108	H	-2.85279	-5.37795	-4.06169
C	-2.74934	0.78063	4.29127	H	-1.25071	-5.23689	-4.81108
C	-4.81638	1.08169	3.03519	C	5.49135	-1.05507	0.71625
C	-4.10862	0.88246	1.84690	C	5.86325	-1.07037	2.05283
C	-2.06534	0.58455	3.08929	H	5.31094	-0.92043	2.96530
H	-2.23228	0.74315	5.24116	N	6.61562	-1.31128	-0.03576
H	-5.88108	1.27481	3.02577	N	7.65072	-1.48212	0.77536
O	-0.70271	0.33239	3.00778	N	7.19107	-1.33467	2.04726
O	-4.69124	0.91478	0.58619	C	8.08265	-1.49492	3.18977
C	0.06534	0.26121	4.24308	H	7.90289	-0.68978	3.90105
H	1.08754	0.05731	3.93064	H	7.90757	-2.45929	3.66772
H	-0.30459	-0.54986	4.87483	H	9.10819	-1.44340	2.82847

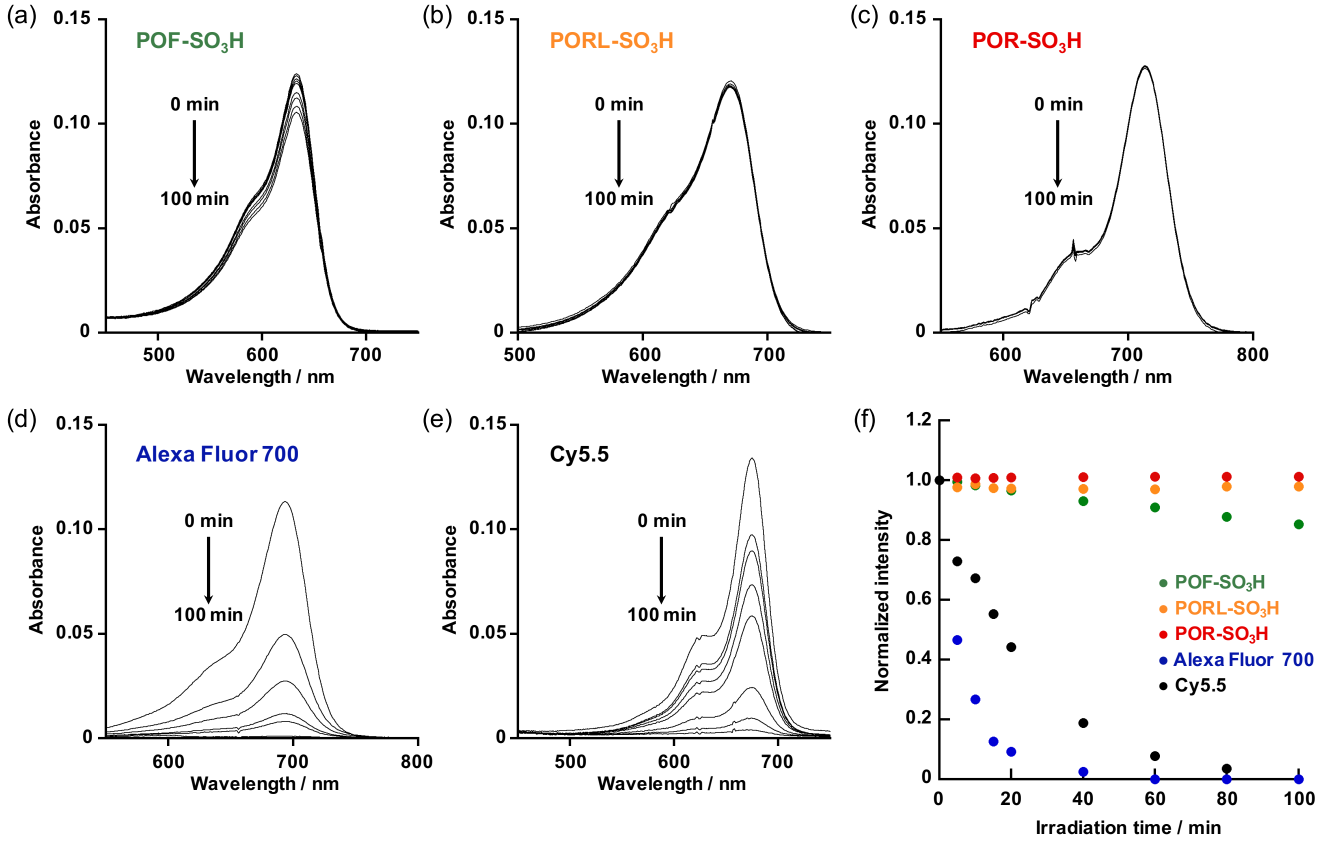

Fig. S5 Absorption spectral changes of (a) POF-SO_3H, (b) PORL-SO_3H, (c) POR-SO_3H, (d) Alexa Fluor 700, and (e) Cy5.5 upon irradiation with a xenon lamp. (f) Normalized absorption intensity was plotted as a function of irradiation time with a Xe lamp.

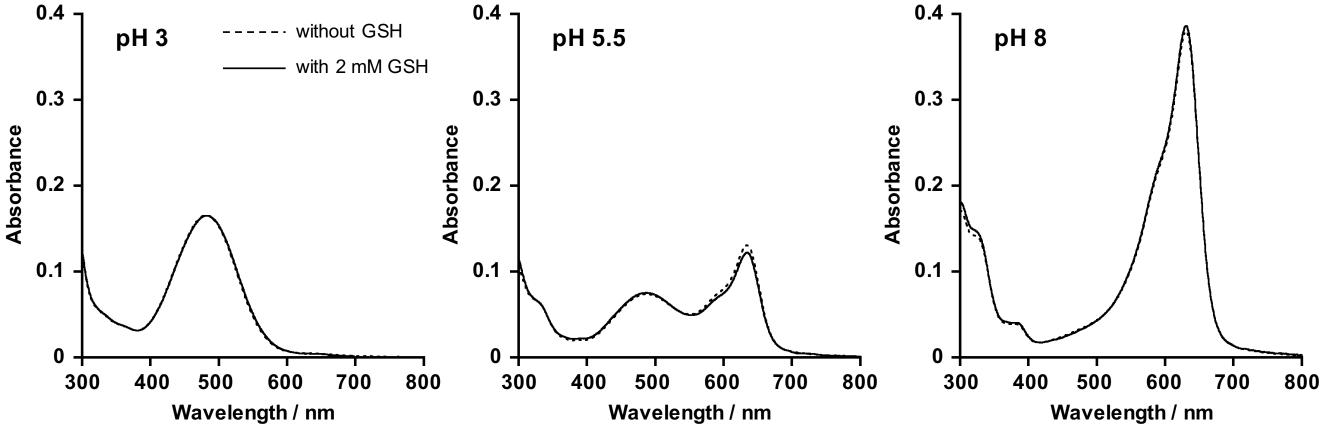

Fig. S6 Absorption spectra of Et_2NPOF (10 μM) in a pH-buffered solution at pH 3, 5.5 and 8 in the absence (dashed line) or presence (solid line) of 2 mM GSH. The spectra were measured 8 h after preparation of the solution.

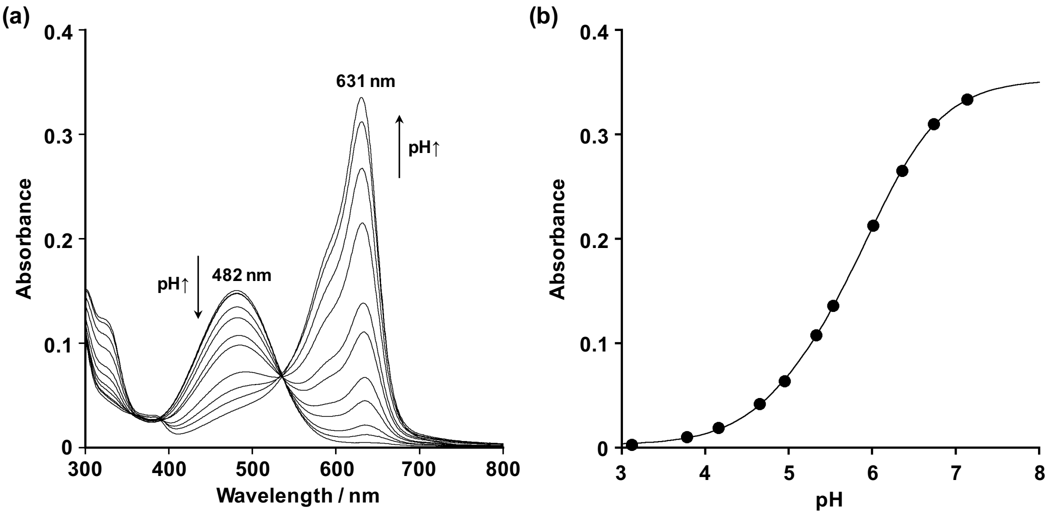

Fig. S7 (a) UV-vis absorption titration spectra of Et_2NPOF (10 μM) in various pH buffer solutions from pH 3.12 to pH 7.14. (b) Plots of the absorbance at 631 nm as a function of pH. The titration data were fitted by non-linear regression to Eq. 4 (R = 0.999997).

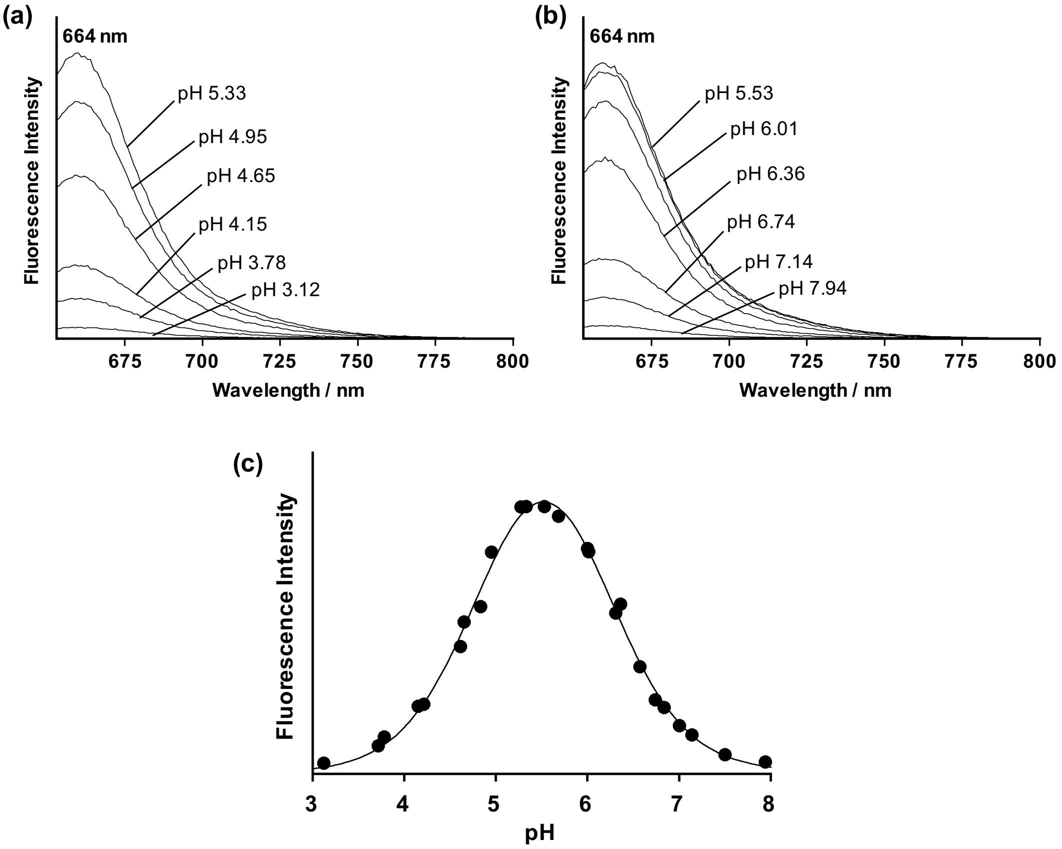

Fig. S8 Fluorescence titration of Et_2NPOF (1 μM) monitored with excitation at 633 nm. (a) Emission spectra in the indicated pH buffer (pH 3.12–5.33). (b) Emission spectra in the indicated pH buffer (pH 5.53–7.94). (c) Plots of the fluorescence intensities at 664 nm as a function of pH. The titration data were fitted by non-linear curve regression to Eq. 5' (R = 0.996).

Table S4. Acidity constants of Et_2NPOF.

	pK_{11}	pK_{12}	pK_{21}	pK_{22}	K_T	pK_{a1}	pK_{a2}
A_{630}	5.48	5.10	5.53	5.91	0.42	4.95	6.06
I_{ex633}	5.42	5.01	5.60	6.02	0.39	4.87	6.16
average	5.45	5.05	5.57	5.96	0.40	4.91	6.11

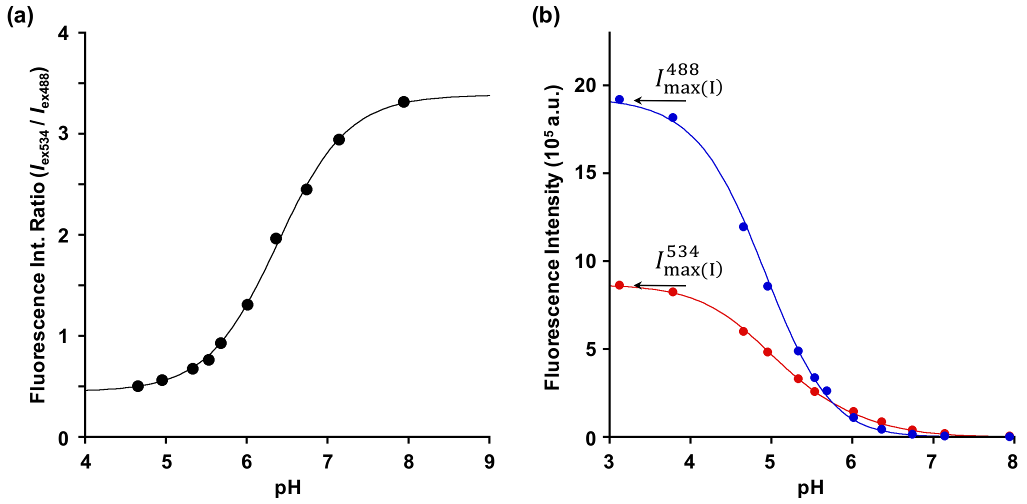

Fig. S9 (a) Plots of the fluorescence intensity ratio of Et_2NPOF (1 μM) at excitation wavelengths of 534 and 488 nm (I_{ex534}/ I_{ex488}) as a function of pH. The data were fitted by a non-linear regression method shown in Eq. 8 (R = 0.99951). (b) Plots of the fluorescence intensity at 664 nm in pH buffer solutions observed upon excitation at 534 nm (red) and 488 nm (blue). The titration data were fitted by a non-linear curve regression method shown in Eqs. 6 and 7 (R = 0.99949 and 0.9997 for I_{ex534} and I_{ex488}, respectively).

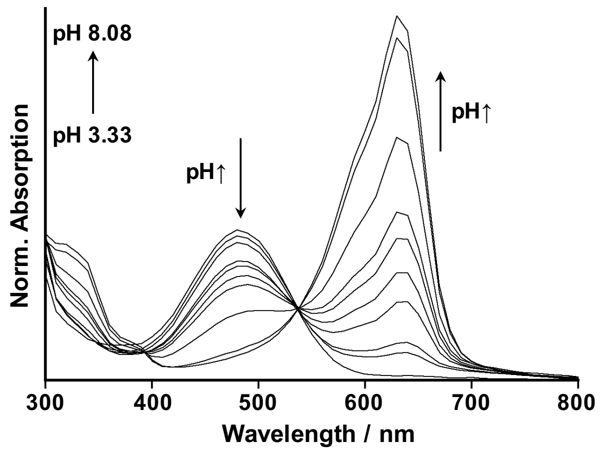

Fig. S10 UV-vis absorption titration spectra of Et_2NPOF-dex (40 μM) in various pH buffer solutions from pH 3.33 to pH 8.08.

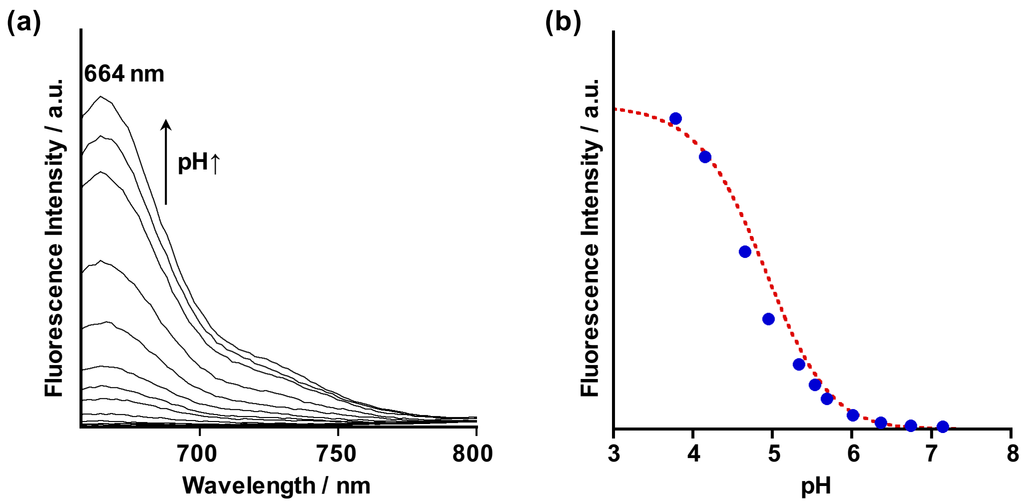

Fig. S11 Fluorescence titration of Et_2NPOF-dex (1 μM) observed upon excitation at 488 nm. (a) Emission spectra in various pH buffer solutions from pH 3.78 to pH 7.14. (b) Plots of the fluorescence intensities at 664 nm (blue closed circle) as a function of pH together with the fitting curve of Et_2NPOF shown in Fig. S7b (dashed line in red).

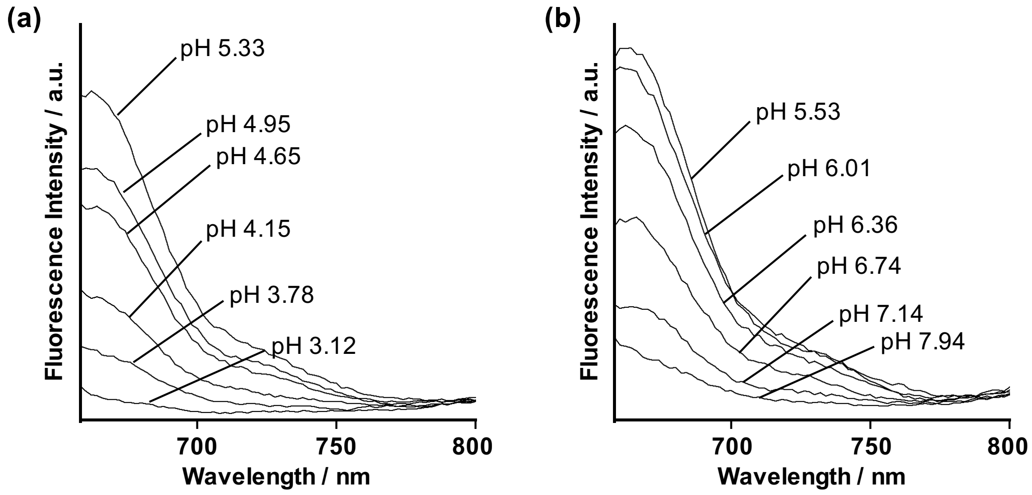

Fig. S12 Fluorescence titration of Et_2NPOF-dex (1 μM) monitored with excitation at 633 nm. (a) Emission spectra in the indicated pH buffer (pH 3.12–5.33). (b) Emission spectra in the indicated pH buffer (pH 5.68–7.14).

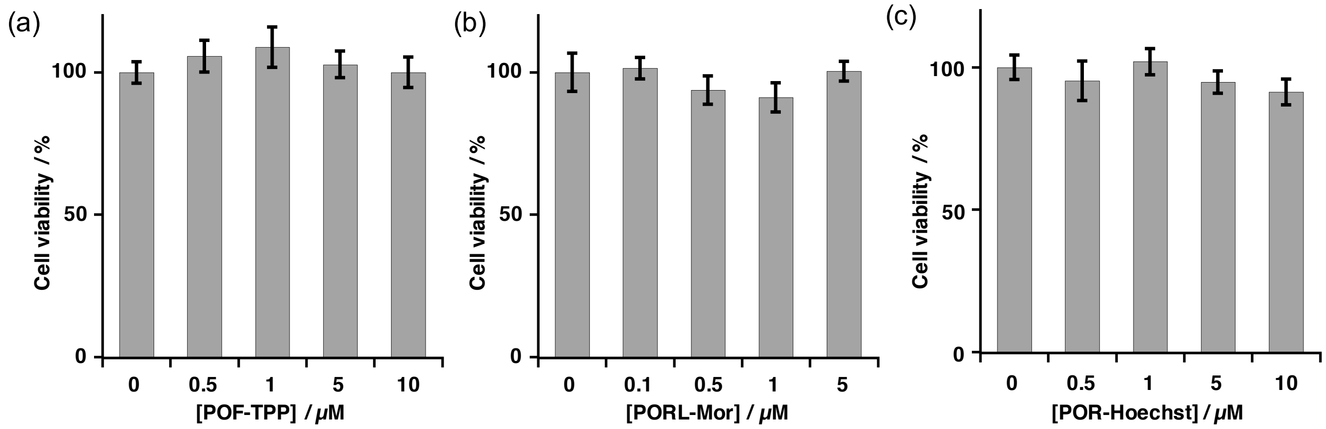

Fig. S13 Cell viability determined by MTT assay. The cells incubated with each concentration of (a) POF-TPP (0, 0.5, 1, 5, 10 μM), (b) PORL-Mor (0, 0.1, 0.5, 1, 5 μM), (c) POR-Hoechst (0, 0.5, 1, 5, 10 μM) in DMEM containing 0.5% DMSO and 0.05% pluronic F-127 in a CO_2 incubator. The incubation time was 2 h for PORL-Mor and POR-Hoechst and 8 h for POF-TPP, respectively. The results are expressed as percentages of the dye-free controls. All data are presented as mean standard deviation (n > 12).

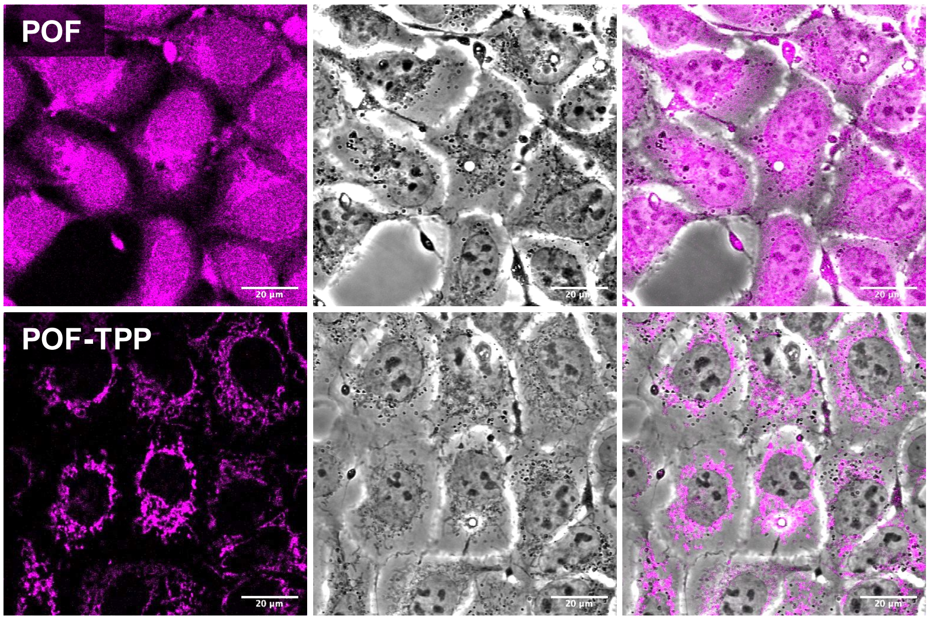

Fig. S14 Confocal images of A431 cells stained with POF-acetate (top) and POF-TPP (bottom) acquired with λ_{ex} = 635 nm and λ_{em} = 660–760 nm. The corresponding bright-filed and merged images are shown in the middle and right columns, respectively. Scale bar = 20 μm.

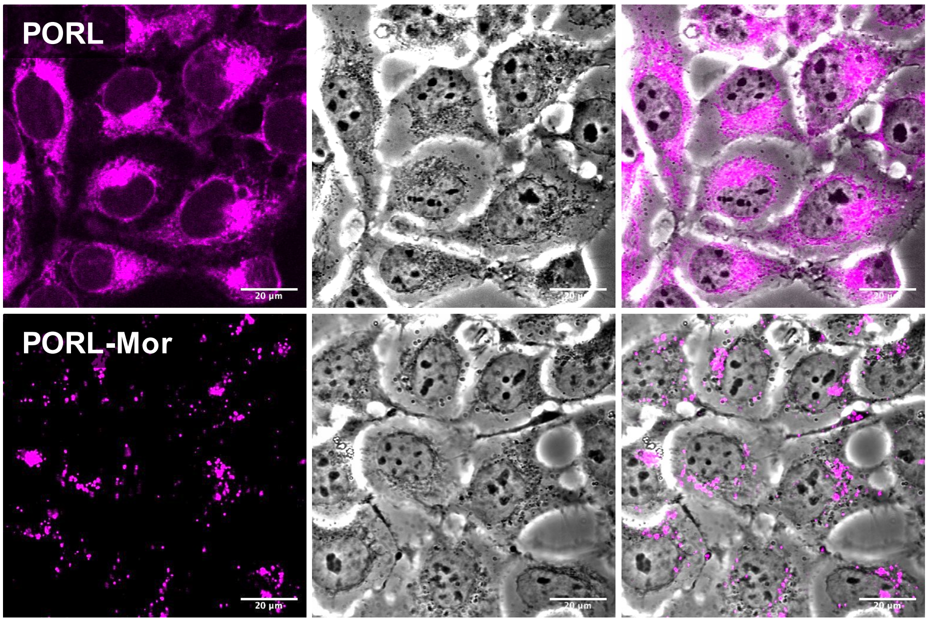

Fig. S15 Confocal images of A431 cells stained with PORL (top) and PORL-Mor (bottom) acquired with λ_{ex} = 635 nm and λ_{em} = 660–760 nm. The corresponding bright-filed and merged images are shown in the middle and right columns, respectively. Scale bar = 20 μm.

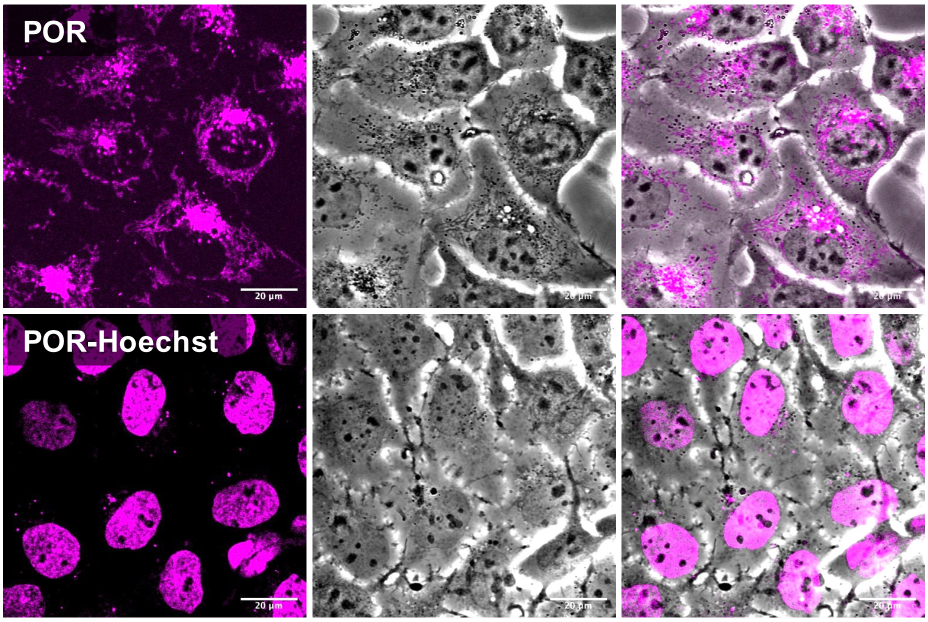

Fig. S16 Confocal images of A431 cells stained with POR (top) and POR-Hoechst (bottom) acquired with λ_{ex} = 635 nm and λ_{em} = 660–760 nm. The corresponding bright-filed and merged images are shown in the middle and right columns, respectively. Scale bar = 20 μm.

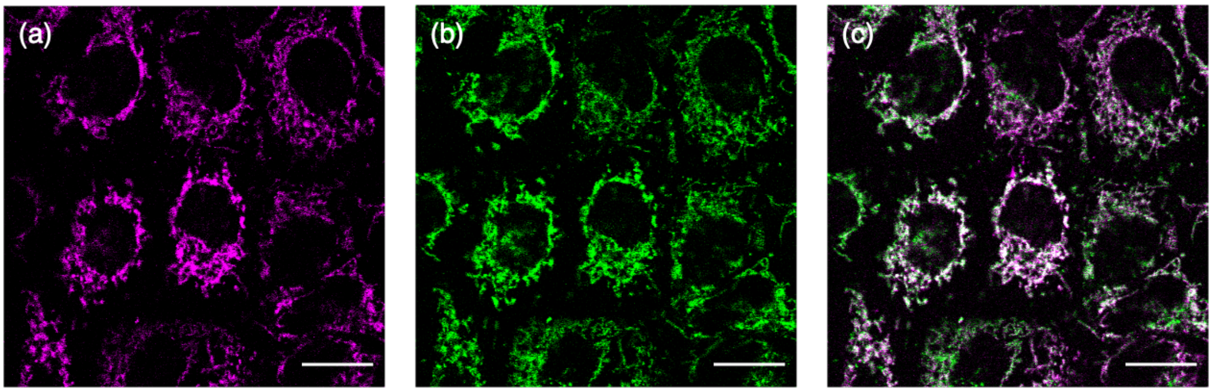

Fig. S17 Confocal images of A431 cells stained with (a) POF-TPP (λ_{ex} = 635 nm, λ_{em} = 660–760 nm) and (b) MitoTracker Green (MTG, λ_{ex} = 473 nm, λ_{em} = 490–590 nm). Images were recorded after washing with DMEM. (c) Merged image of (a) and (b). Scale bar = 20 µm.

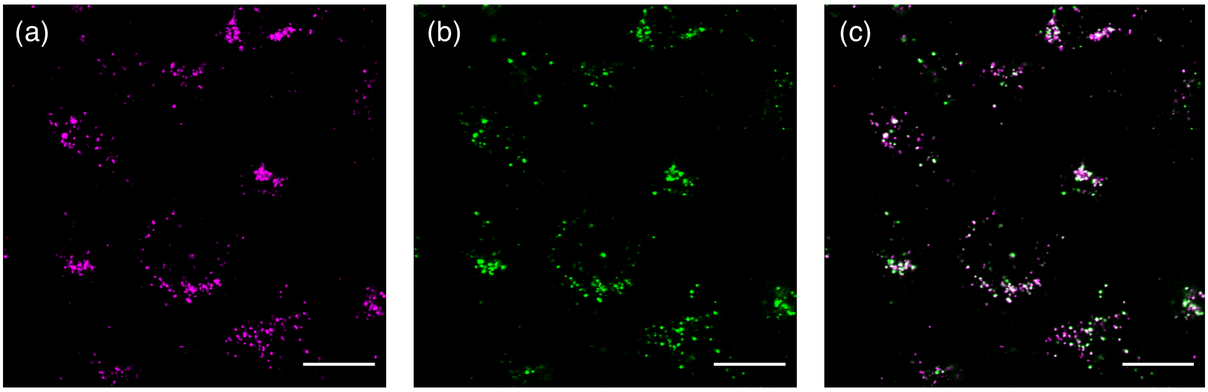

Fig. S18 Confocal images of A431 cells stained with (a) PORL-Mor (λ_{ex} = 635 nm, λ_{em} = 660–760 nm) and (b) LysoTracker Green (LTG, λ_{ex} = 473 nm, λ_{em} = 490–590 nm). Images were recorded after washing with DMEM. (c) Merged image of (a) and (b). Scale bar = 20 µm.

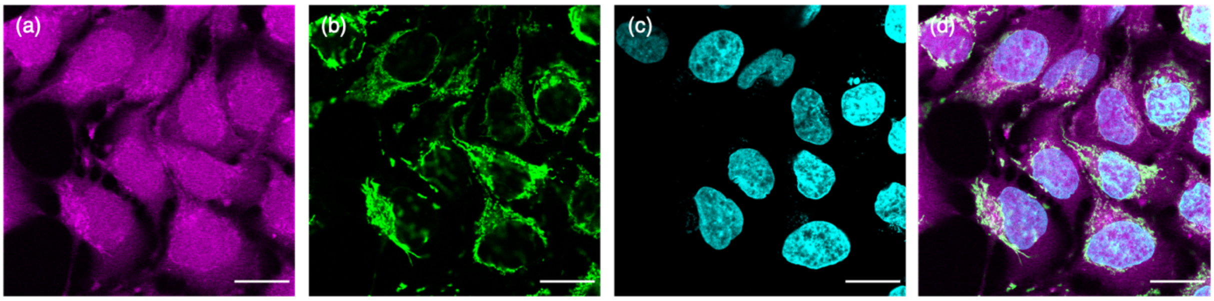

Fig. S19 Confocal images of A431 cells stained with (a) AcPOF (λ_{ex} = 640 nm, λ_{em} = 650–750 nm), (b) MTG (λ_{ex} = 488 nm, λ_{em} = 500–600 nm), and (c) Hoechst 33342 (λ_{ex} = 405 nm, λ_{em} = 420–520). Images were recorded after washing with DMEM. (d) Merged image of (a)–(c). Scale bar = 20 µm.

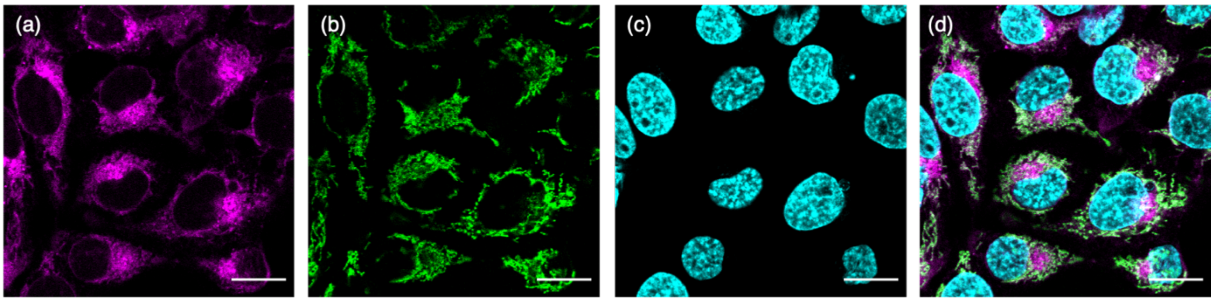

Fig. S20 Confocal images of A431 cells stained with (a) PORL (λ_{ex} = 635 nm, λ_{em} = 660–760 nm), (b) MTG (λ_{ex} = 473 nm, λ_{em} = 490–590 nm), and (c) Hoechst 33342 (λ_{ex} = 405 nm, λ_{em} = 420–520). Images were recorded after washing with DMEM. (d) Merged image of (a)–(c). Scale bar = 20 μm.

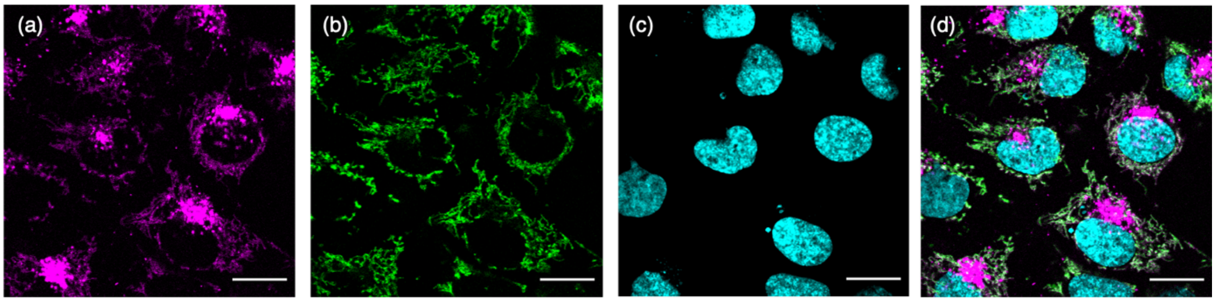

Fig. S21 Confocal images of A431 cells stained with (a) POR (λ_{ex} = 635 nm, λ_{em} = 660–760 nm), (b) MTG (λ_{ex} = 473 nm, λ_{em} = 490–590 nm), and (c) Hoechst 33342 (λ_{ex} = 405 nm, λ_{em} = 420–520). Images were recorded without washing process. (d) Merged image of (a)–(c). Scale bar = 20 μm.

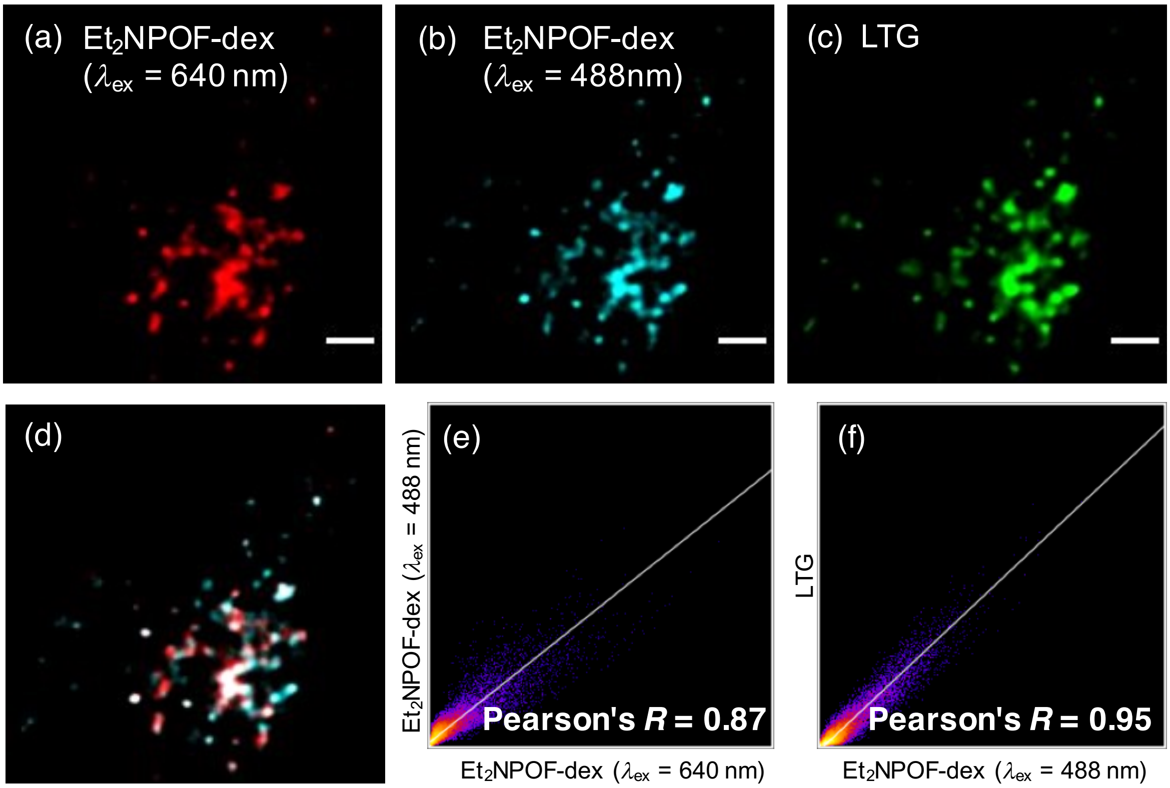

Fig. S22 Confocal images of HeLa cells stained with Et_2NPOF-dex (a and b) and LTG (c). The images were acquired with (a) λ_{ex} = 640 nm and λ_{em} = 650–750 nm, (b) λ_{ex} = 488 nm and λ_{em} = 650–750 nm, and (c) λ_{ex} = 488 nm and λ_{em} = 500–600 nm. Scale bar = 2 µm. (d) Merged image of (a) and (b), where red. white, and cyan spots represent weakly acidic (pH ~6.5), moderately acidic (pH ~5.5), and strongly acidic (pH ~4.0), respectively. (e) Pearson correlation coefficient plot of panels (a) and (b). (f) Pearson correlation coefficient plot of panels (b) and (c). A smaller Pearson's R values of 0.87 in (e) indicates that the acidic compartments with different pH values can be discriminated.

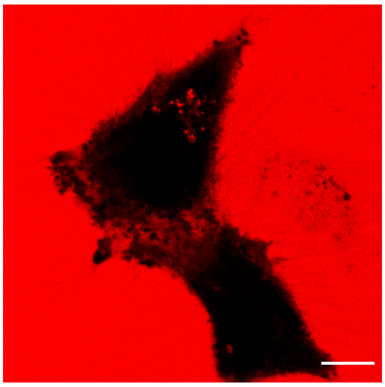

Fig. S23 Confocal images of HeLa cells stained with POF-dex. The image was acquired with λ_{ex} = 640 nm and λ_{em} = 650–750 nm. Scale bar = 20 µm.

3. References

1 D. Türp, M. Wagner, V. Enkelmann and K. Müllen, *Angew. Chem. Int. Ed.* 2011, **50**, 4962–4965.

2 T. Wang, Q.-J. Zhao, H.-G. Hu, S.-C. Yu, X. Liu, L. Liu and Q.-Y. Wu, *Chem. Commun.,* 2012, **48**, 8781–8783.

3 G. Li, Q. Feng, J. Dai and S. Chen, Donor-πacceptor based diboron oxapyrene compounds and application thereof as luminescent materials or host materials in optical or photoelectric devices, 2019, CN 110183475

4 M. Grzybowski, M. Taki, K. Senda, Y. Sato, T. Ariyoshi, Y. Okada, R. Kawakami, T. Imamura and S. Yamaguchi, *Angew. Chem. Int. Ed.,* 2018, **57**, 10137–10141.

5 E. F.-Megia, J. Correa, I. R.-Meizoso and R. Riguera, *Macromolecules*, 2006, **39**, 2113–2120.

6 A. Chhen, M. Vaultier and R. Carrié, *Tetrahedron Lett*., 1989, **30**, 4953–4956.

7 J. Grünewald, Y. Jin, J. Vance, J. Read, X. Wang, Y. Wan, H. Zhou, W. Ou, H. E. Klock, E. C. Peter, T. Uno, A. Brock and B. Geierstanger, *Bioconjugate Chem*., 2017, **28**, 7, 1906–1915.

8 A. Fukazawa, S. Suda, M. Taki, E. Yamaguchi, M. Grzybowski, Y. Sato, T. Higashiyama and S. Yamaguchi, *Chem. Commun.,* 2016, **52**, 1120–1123.

9 M. Grzybowski, M. Taki and S. Yamaguchi, *Chem.–Eur. J.,* 2017, **23**, 13028–13032.

10 Y. Zhao and D. G. Truhlar, *Theor. Chem. Account,* 2008, **120**, 215–241.

11 a) R. Ditchfield, W. J. Hehre and J. A. Pople, *J. Chem. Phys.,* 1971, **54**, 724–728; b) W. J. Hehre, R. Ditchfield and J. A. Pople, *J. Chem. Phys.,* 1972, **56**, 2257–2261; c) P. C. Hariharan and J. A. Pople, *Theor. Chim. Acta,* 1973, **28**, 213–222; d) M. M. Francl, W. J. Pietro, W. J. Hehre, J. S. Binkley, M. S. Gordon, D. J. DeFrees and J. A. Pople, *J. Chem. Phys.,* 1982, **77**, 3654–3665.

12 A. V. Marenich, C. J. Cramer, D. G. Truhlar, *J. Phys. Chem. B*, 2009, **113**, 6378–6696.

13 Gaussian 16, Revision B.01, M. J. Frisch, G. W. Trucks, H. B. Schlegel, G. E. Scuseria, M. A. Robb, J. R. Cheeseman, G. Scalmani, V. Barone, G. A. Petersson, H. Nakatsuji, X. Li, M. Caricato, A. V. Marenich, J. Bloino, B. G. Janesko, R. Gomperts, B. Mennucci, H. P. Hratchian, J. V. Ortiz, A. F. Izmaylov, J. L. Sonnenberg, D. Williams-Young, F. Ding, F. Lipparini, F. Egidi, J. Goings, B. Peng, A. Petrone, T. Henderson, D. Ranasinghe, V. G. Zakrzewski, J. Gao, N. Rega, G. Zheng, W. Liang, M. Hada, M. Ehara, K. Toyota, R. Fukuda, J. Hasegawa, M. Ishida, T. Nakajima, Y. Honda, O. Kitao, H. Nakai, T. Vreven, K. Throssell, J. A. Montgomery, Jr., J. E. Peralta, F. Ogliaro, M. J. Bearpark, J. J. Heyd, E. N. Brothers, K. N. Kudin, V. N. Staroverov, T. A. Keith, R. Kobayashi, J. Normand, K. Raghavachari, A. P. Rendell, J. C. Burant, S. S. Iyengar, J. Tomasi, M. Cossi, J. M. Millam, M. Klene, C. Adamo, R. Cammi, J. W. Ochterski, R. L. Martin, K. Morokuma, O. Farkas, J. B. Foresman, and D. J. Fox, Gaussian, Inc., Wallingford CT, 2016.

4. NMR Spectra

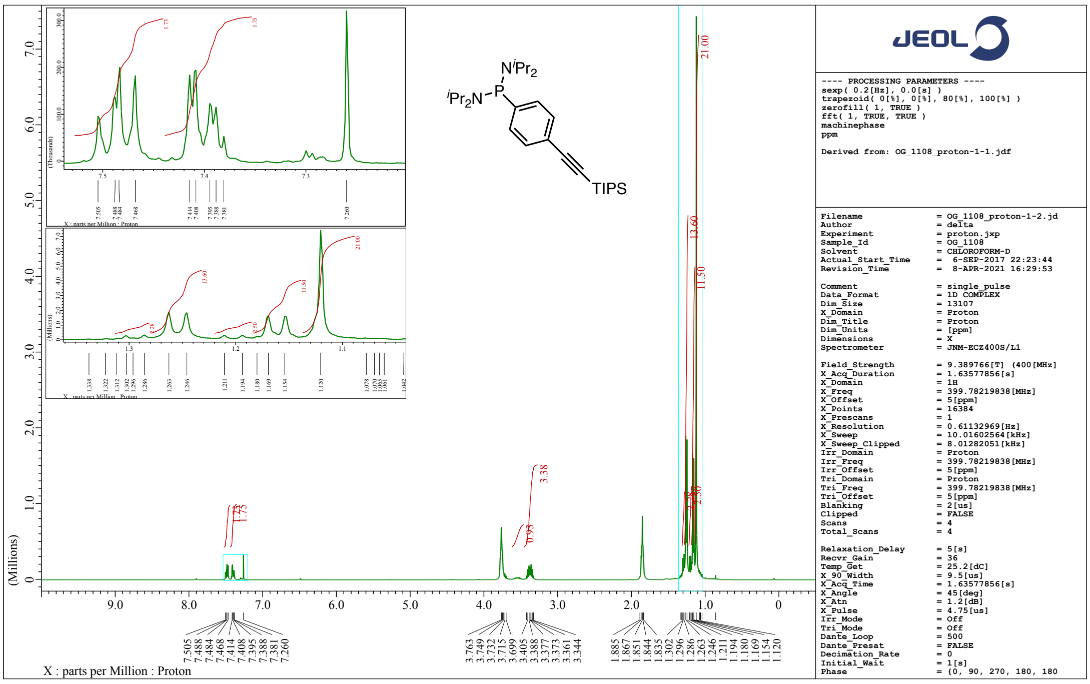

^{1}H NMR spectrum of **2** (500 MHz, $CDCl_3$)

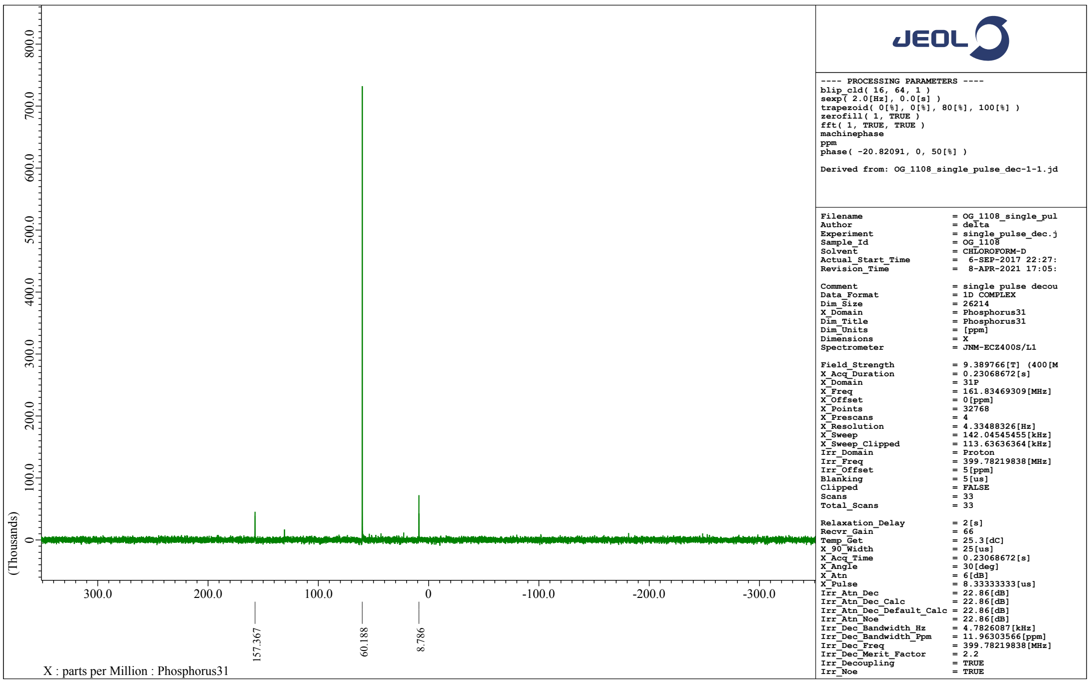

^{31}P NMR spectrum of **2** (162 MHz, $CDCl_3$)

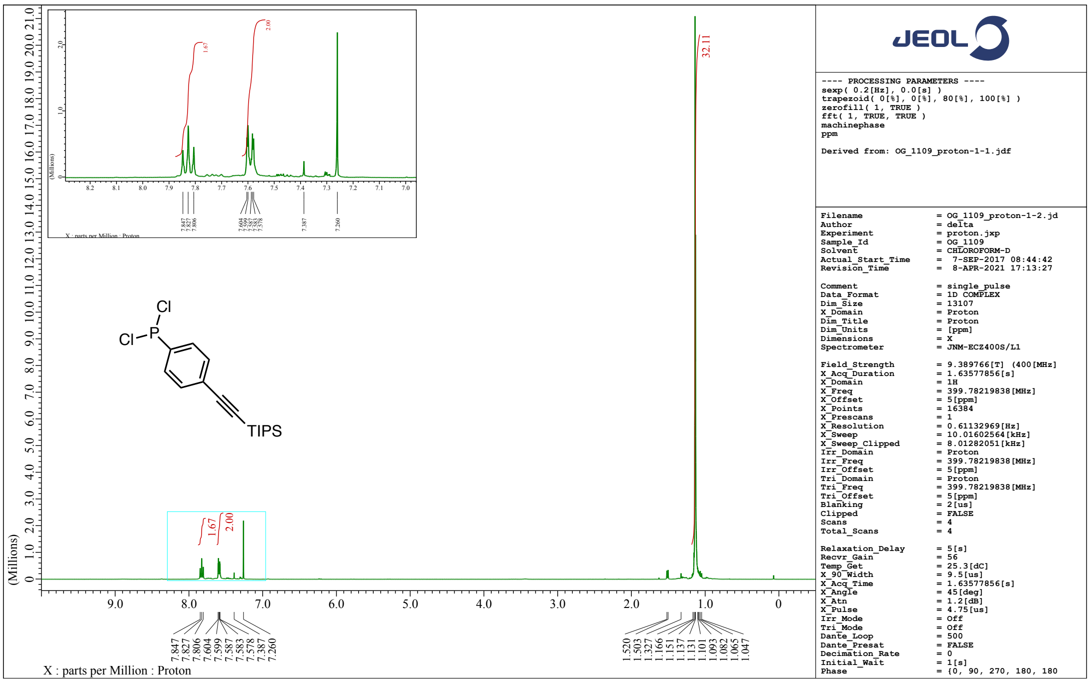

^{1}H NMR spectrum of **3** (500 MHz, $CDCl_3$)

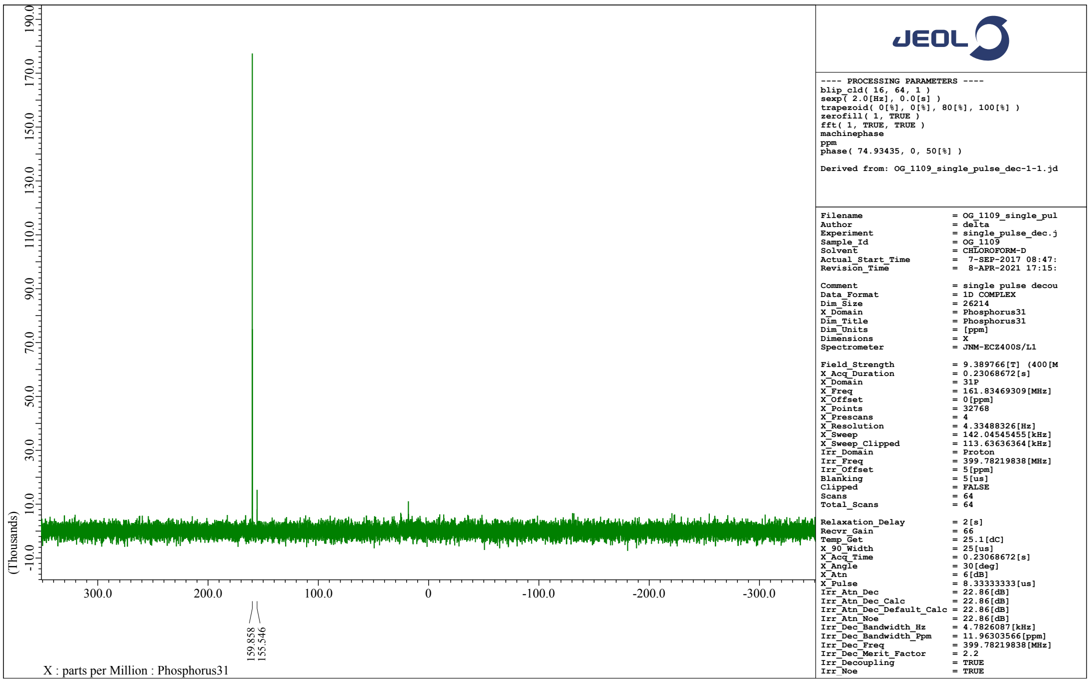

^{31}P NMR spectrum of **3** (162 MHz, $CDCl_3$)

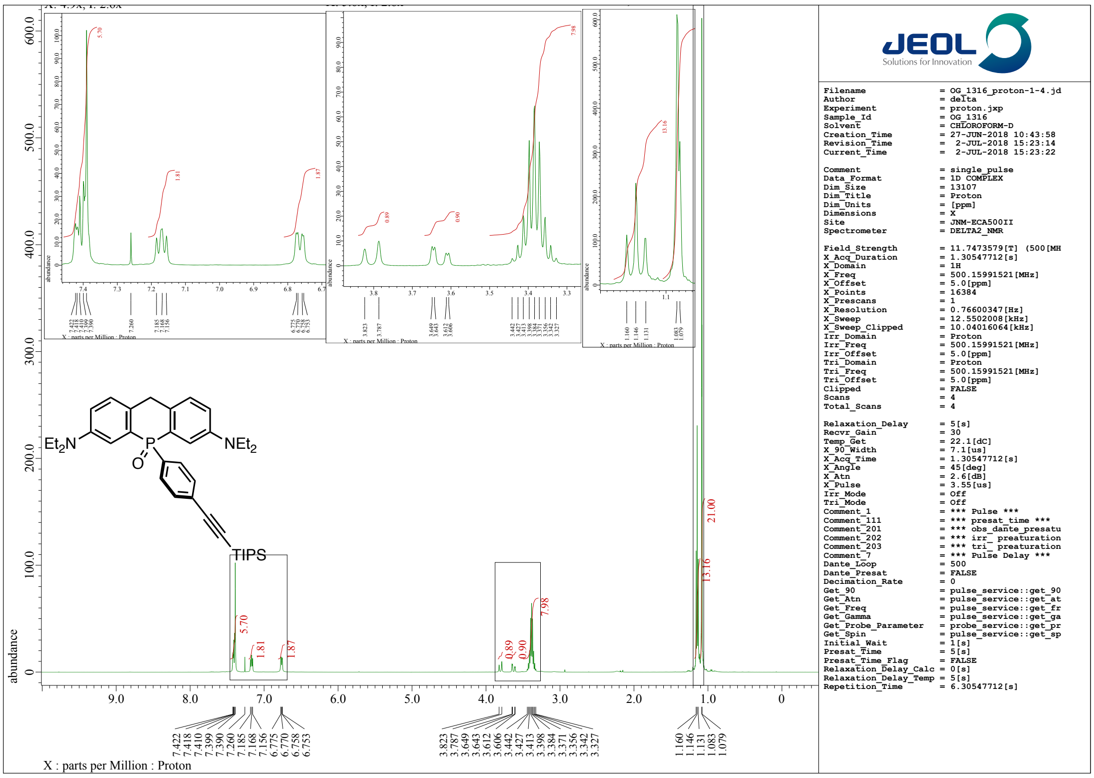

^{1}H NMR spectrum of **4** (500 MHz, $CDCl_3$)

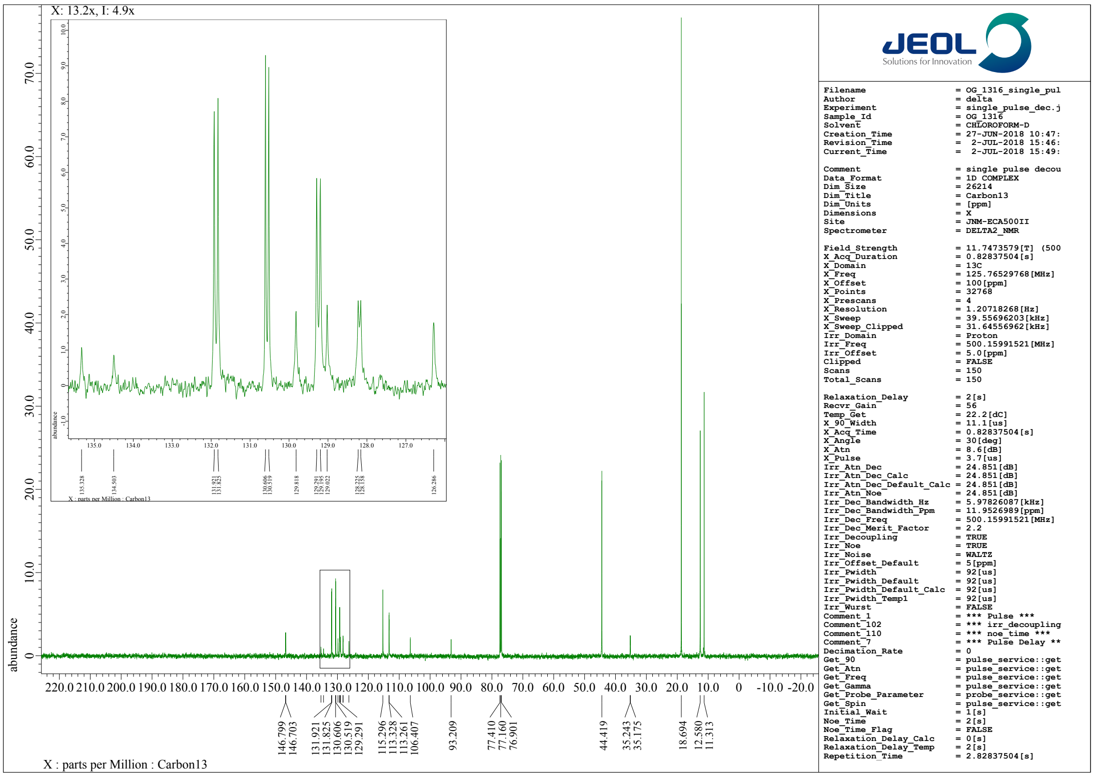

^{13}C NMR spectrum of **4** (125 MHz, $CDCl_3$)

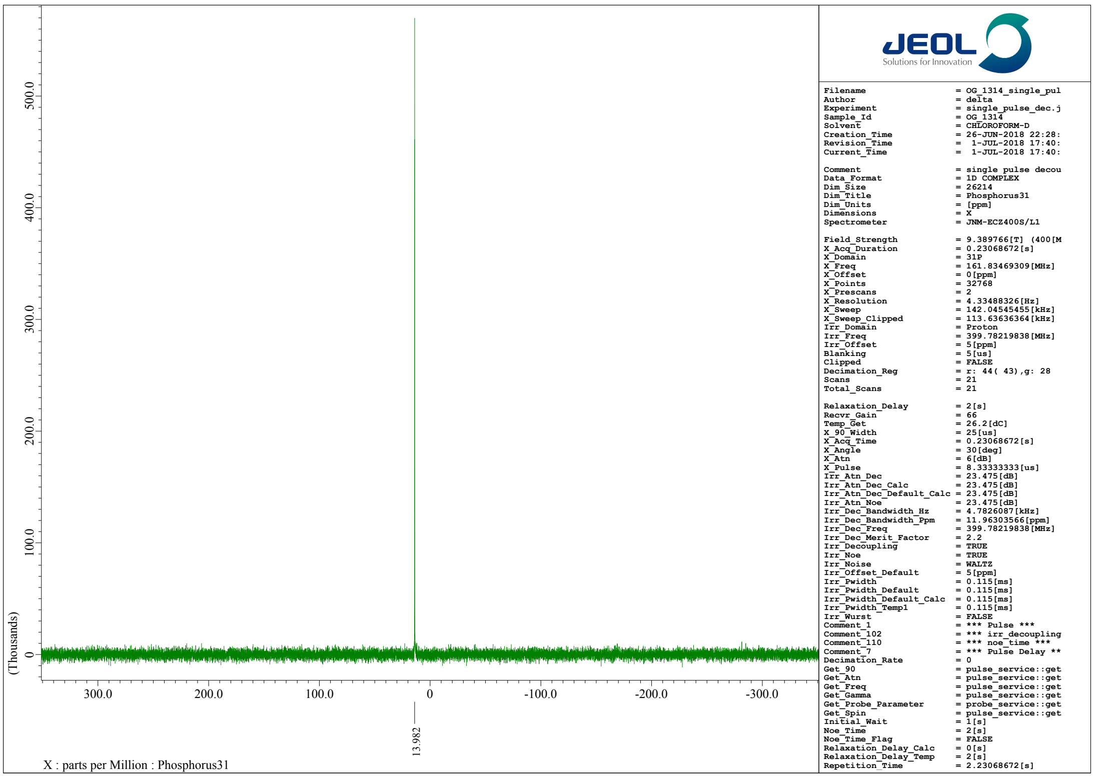

^{31}P NMR spectrum of **4** (162 MHz, $CDCl_3$)

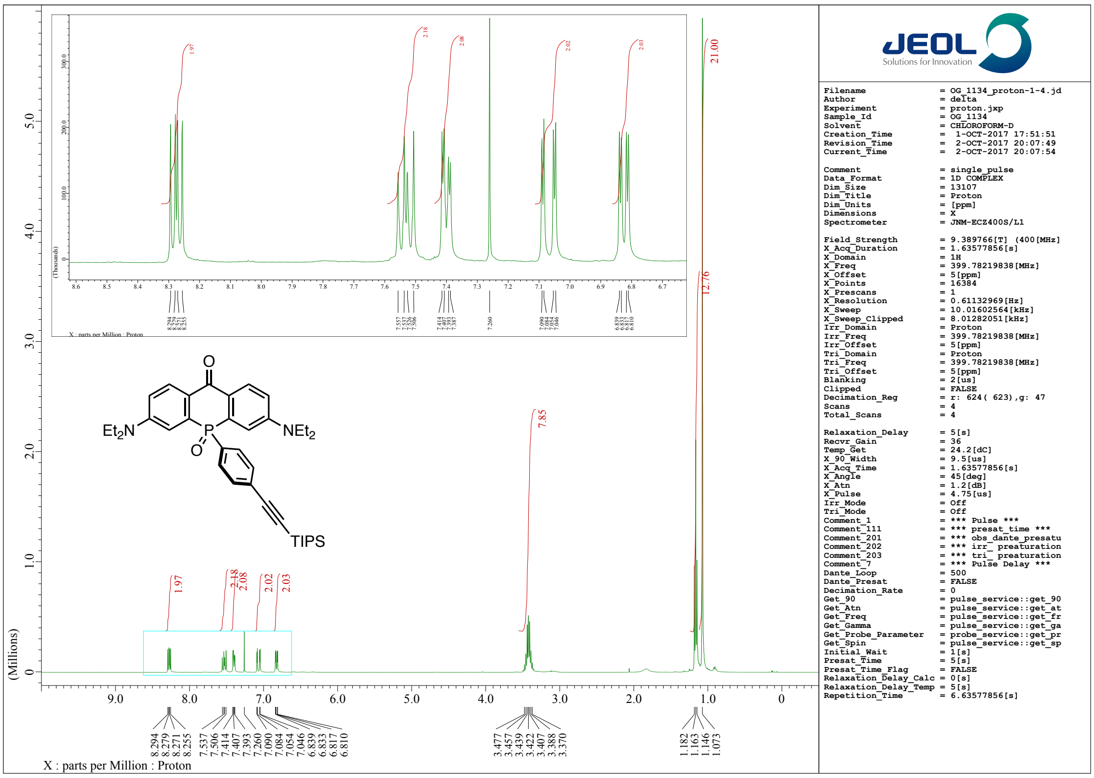

^{1}H NMR spectrum of **5** (400 MHz, $CDCl_3$)

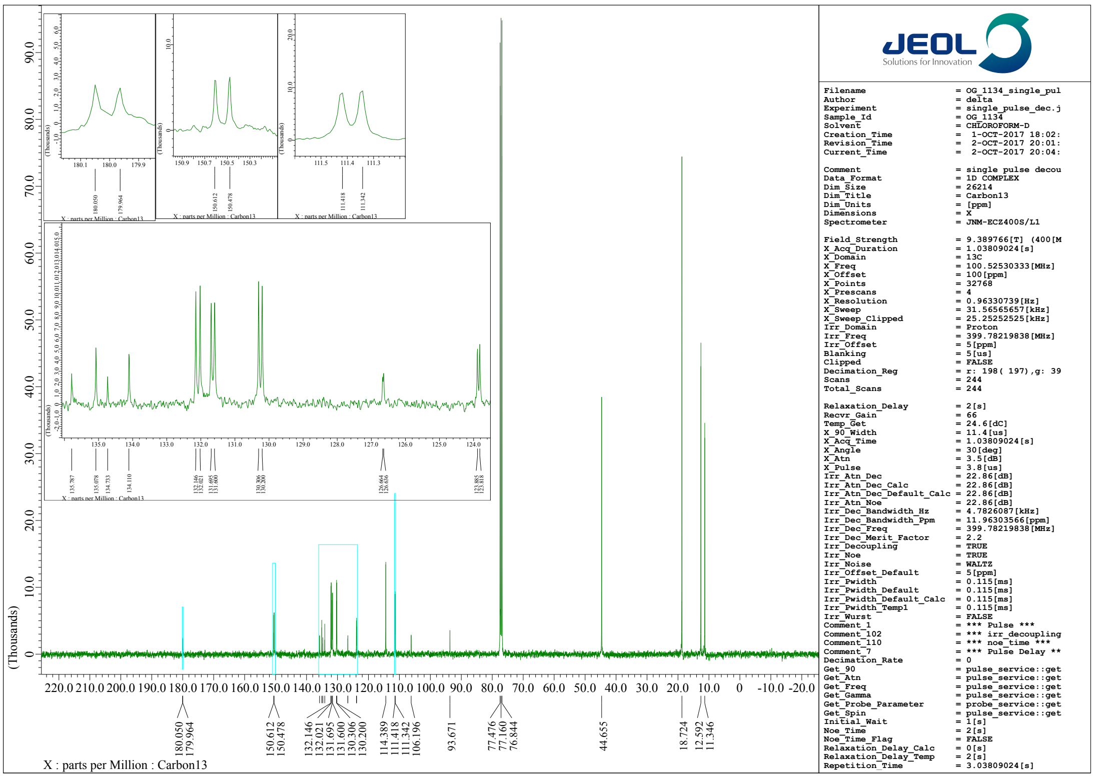

^{1}H NMR spectrum of **5** (100 MHz, $CDCl_3$)

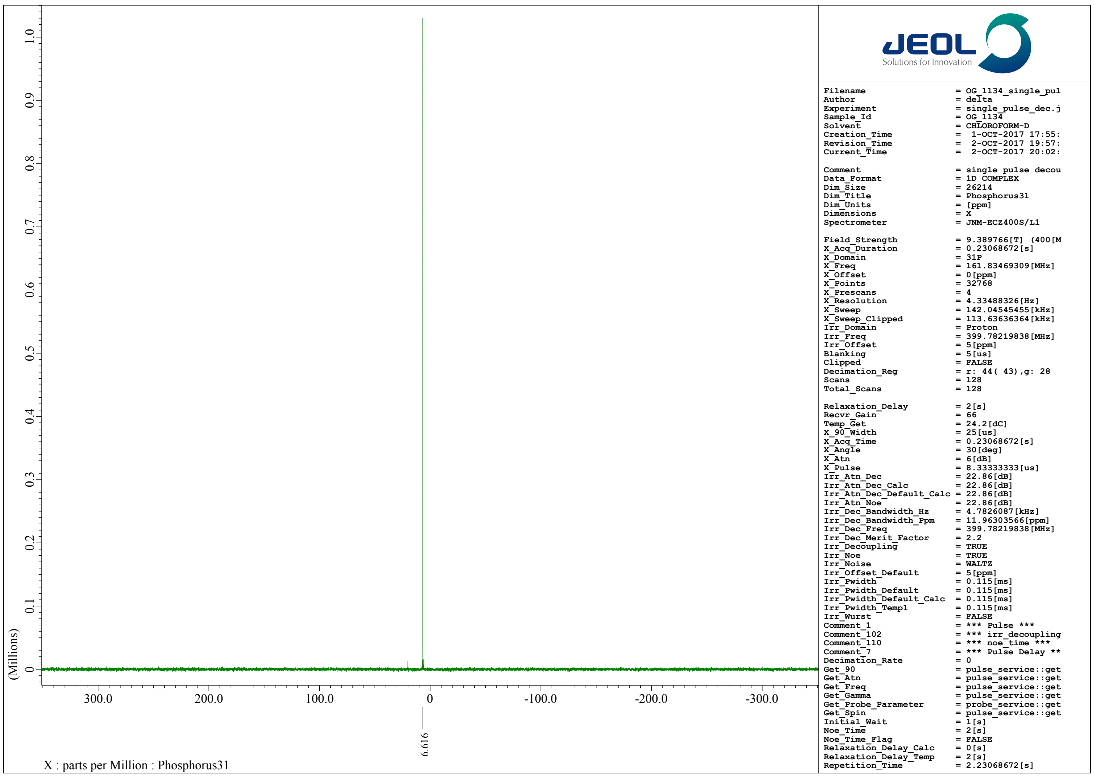

^{31}P NMR spectrum of **5** (162 MHz, $CDCl_3$)

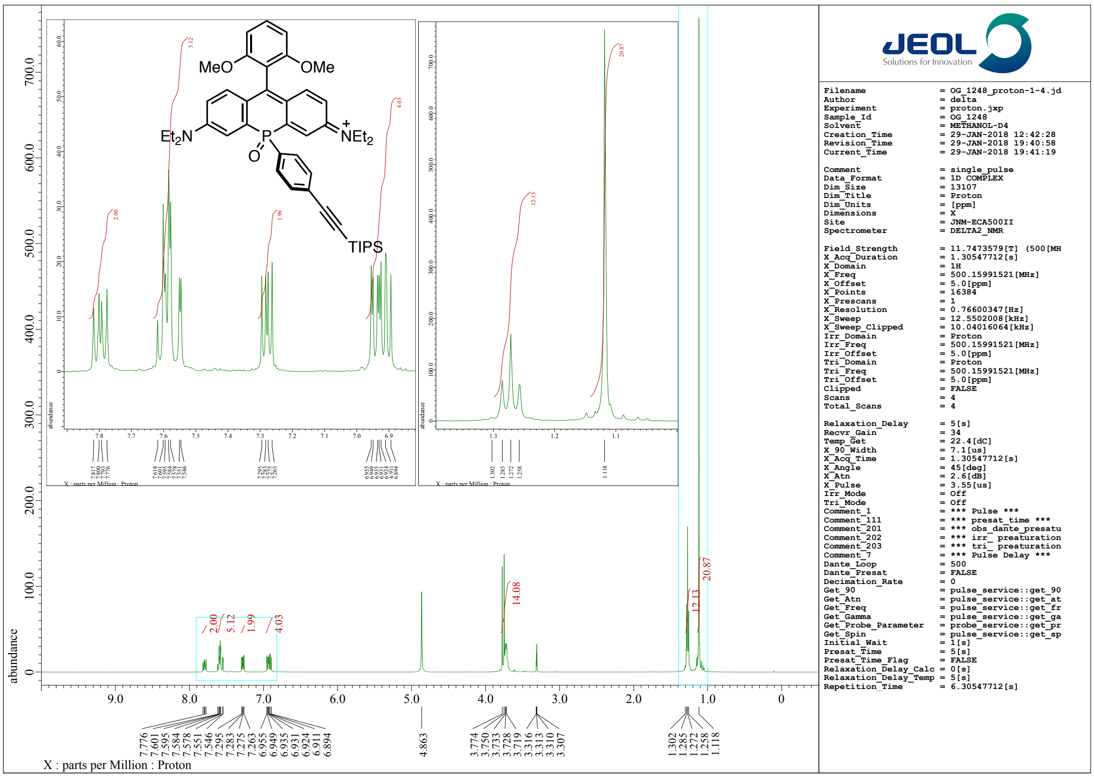

^{1}H NMR spectrum of **6** (500 MHz, CD_3OD)

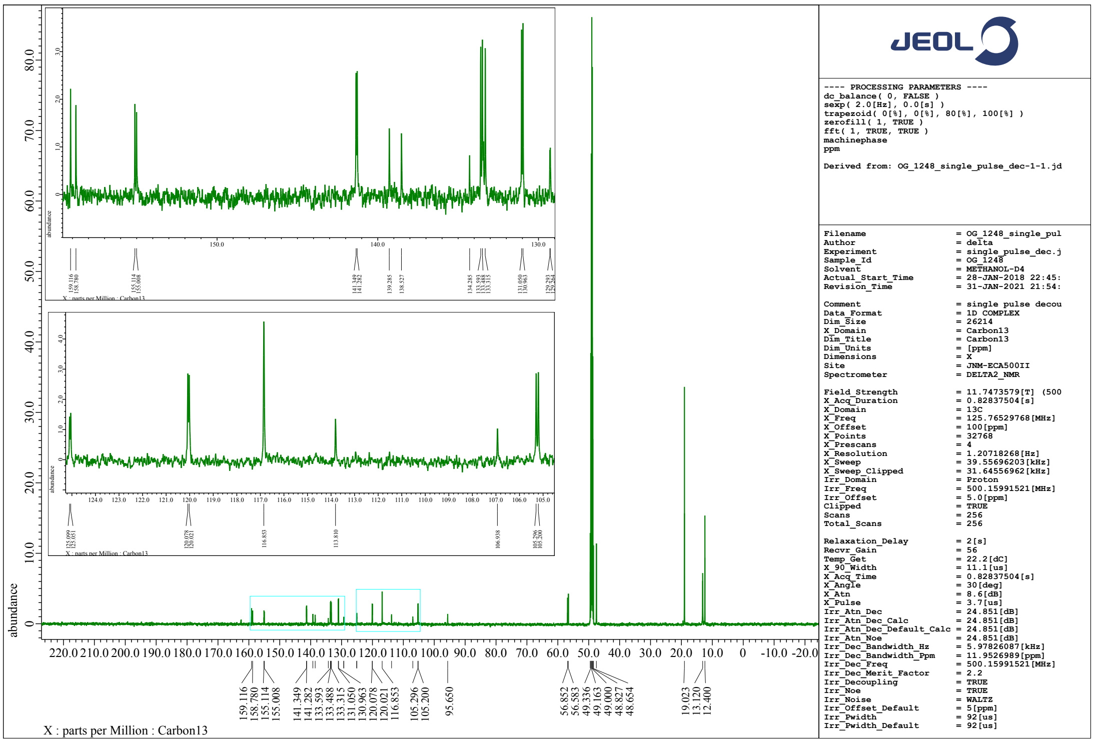

^{13}C NMR spectrum of **6** (125 MHz, CD_3OD)

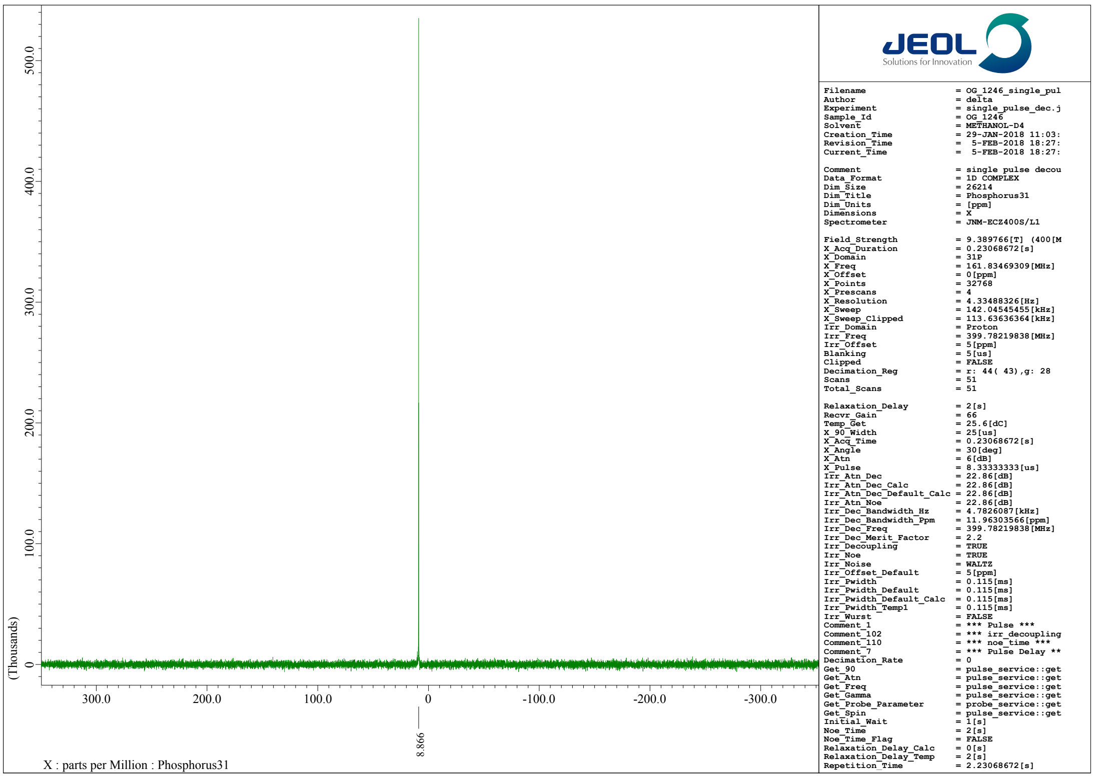

^{31}P NMR spectrum of **6** (162 MHz, CD_3OD)

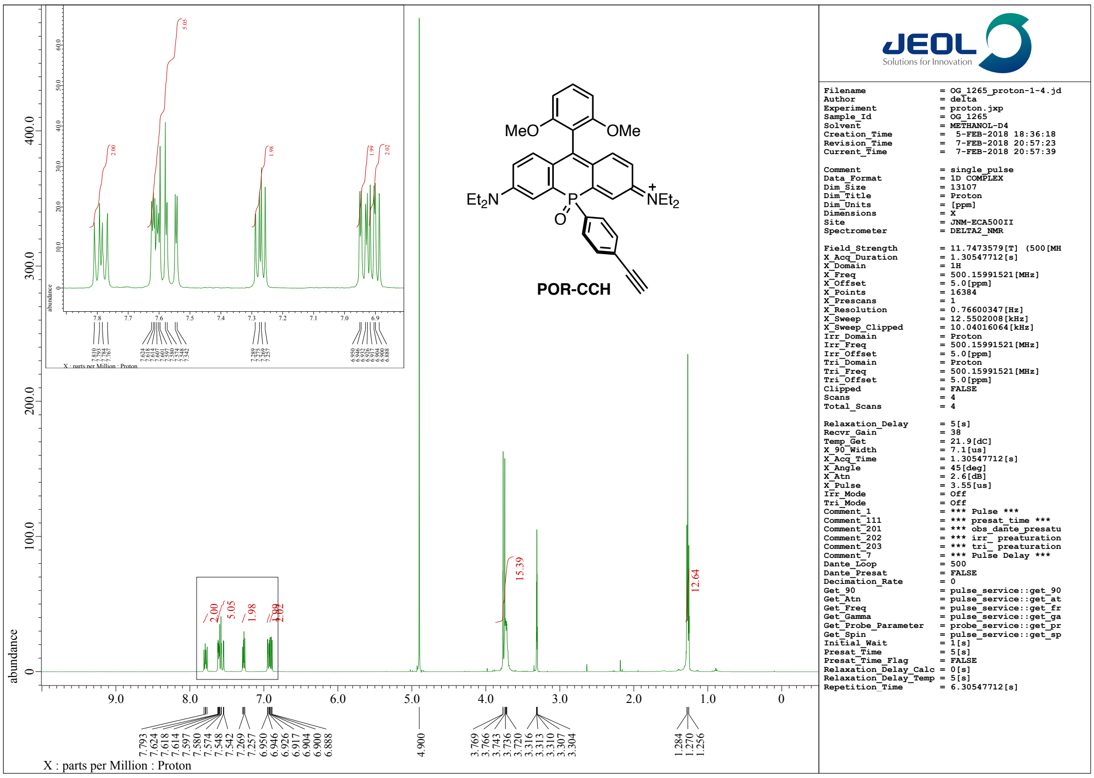

^{1}H NMR spectrum of **POR-CCH** (500 MHz, CD_3OD)

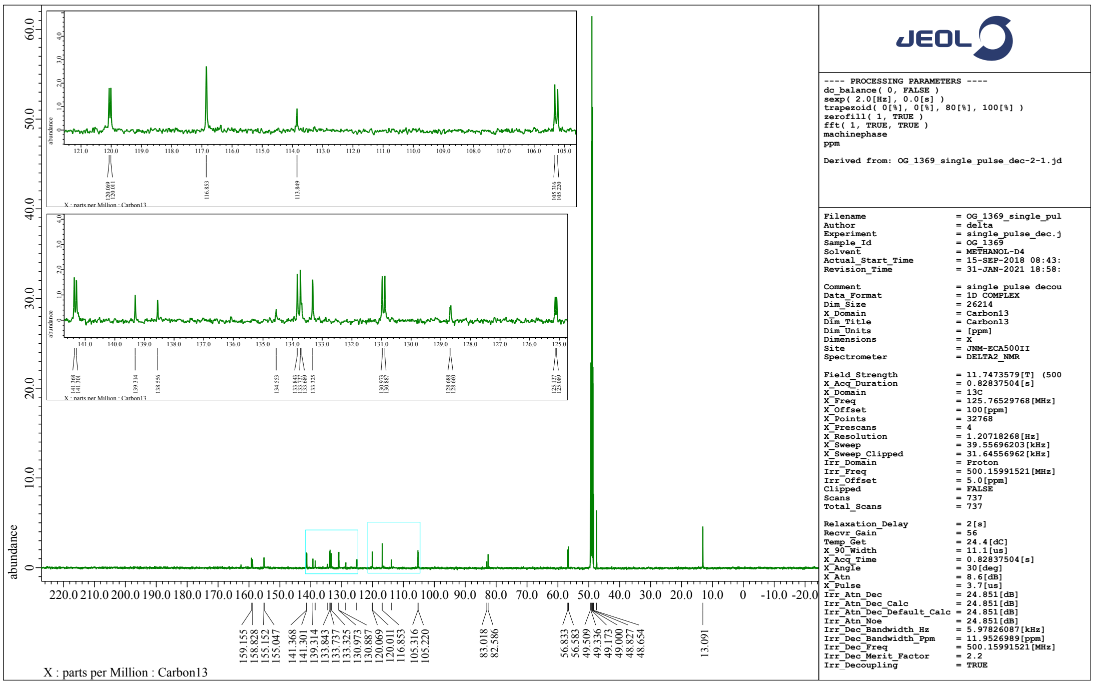

^{13}C NMR spectrum of **POR-CCH** (125 MHz, CD_3OD)

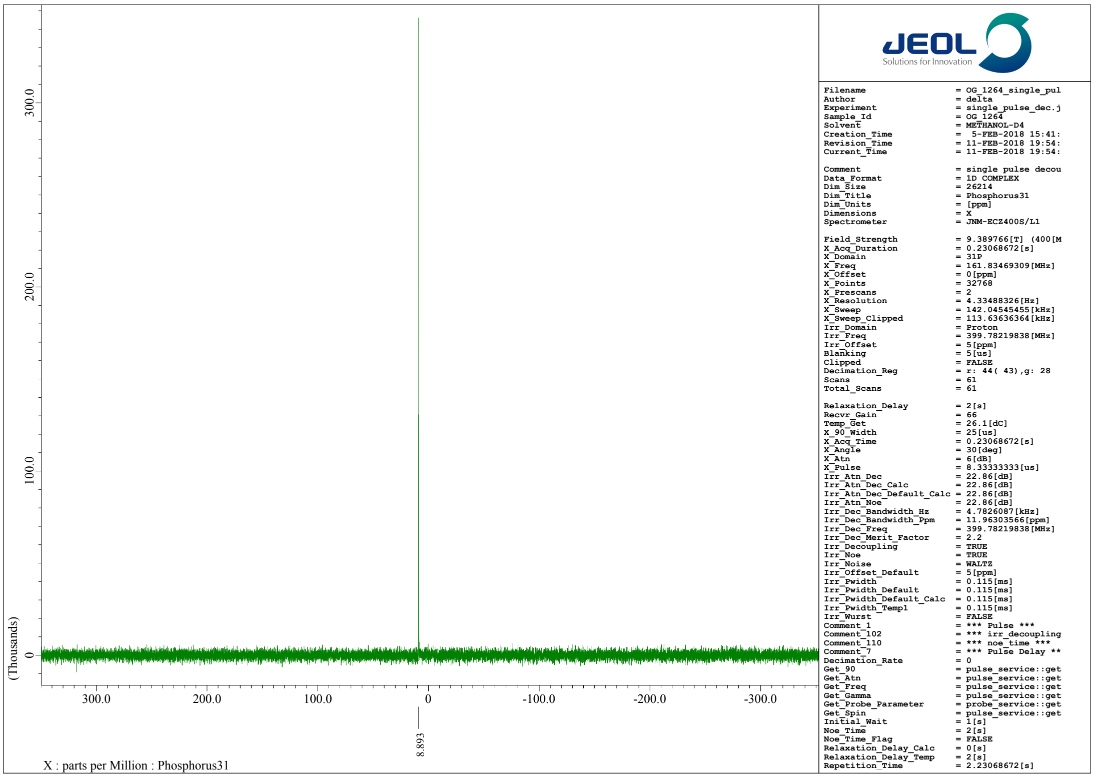

^{31}P NMR spectrum of **POR-CCH** (162 MHz, CD_3OD)

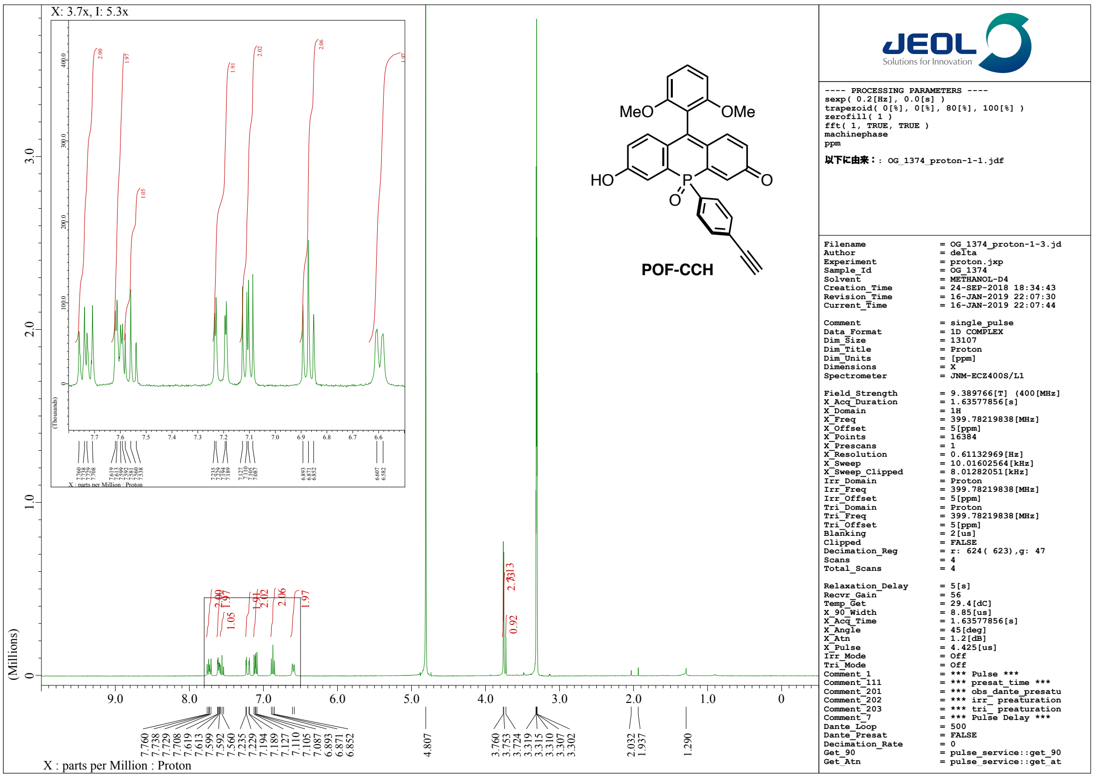

^{1}H NMR spectrum of **POF-CCH** (400 MHz, CD_3OD)

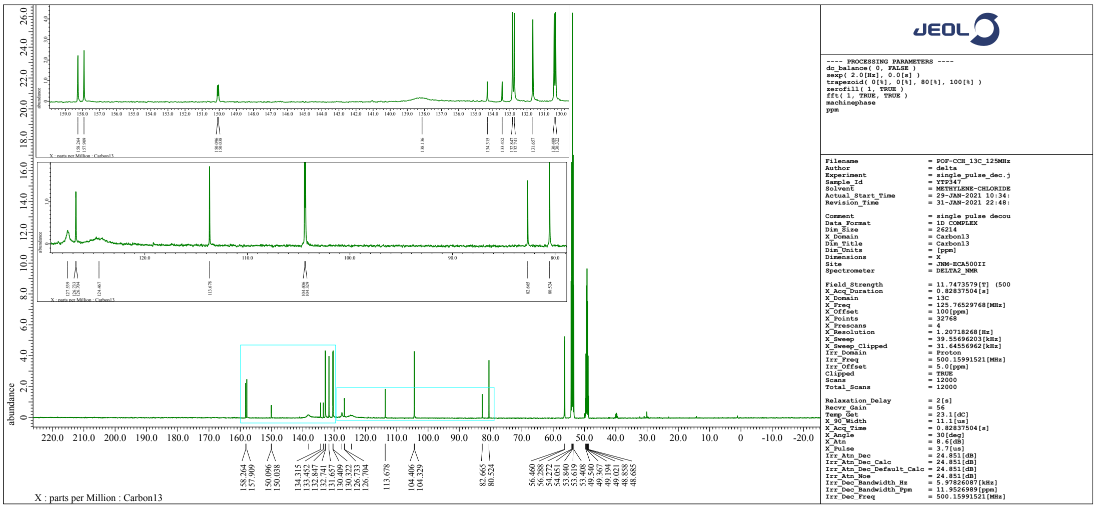

^{13}C NMR spectrum of **POF-CCH** (125 MHz, CH_2Cl_2/CD_3OD = 9/1)

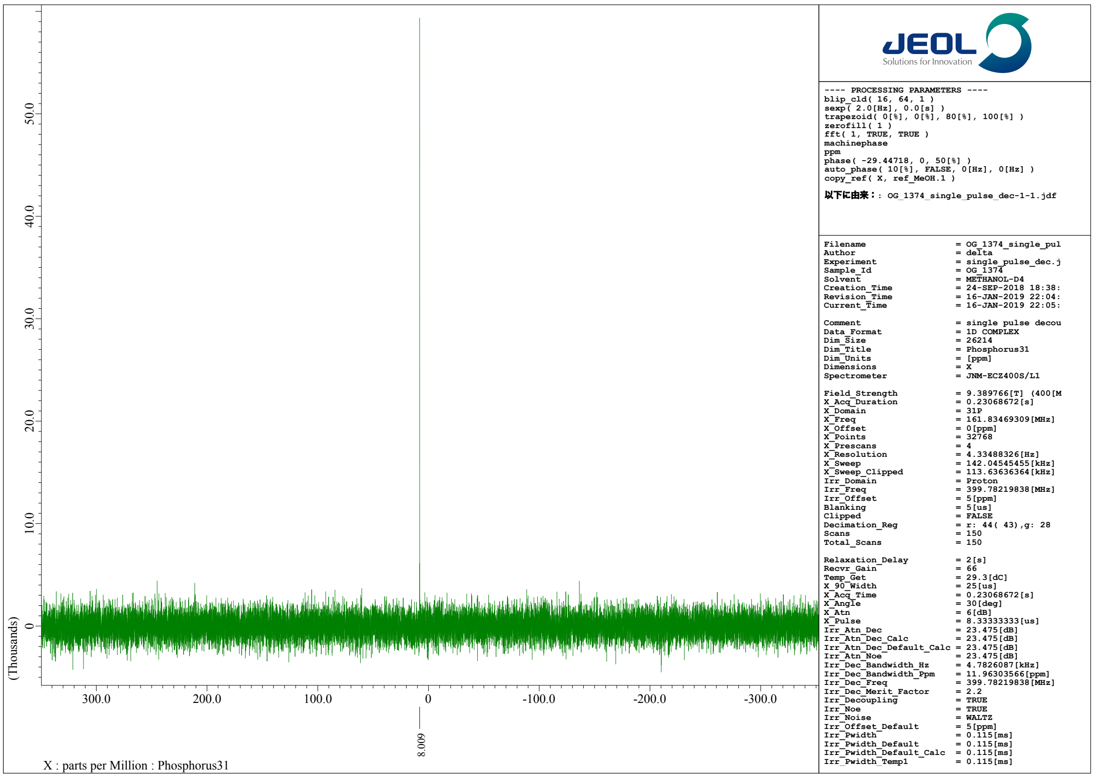

^{31}P NMR spectrum of **POF-CCH** (162 MHz, CD_3OD)

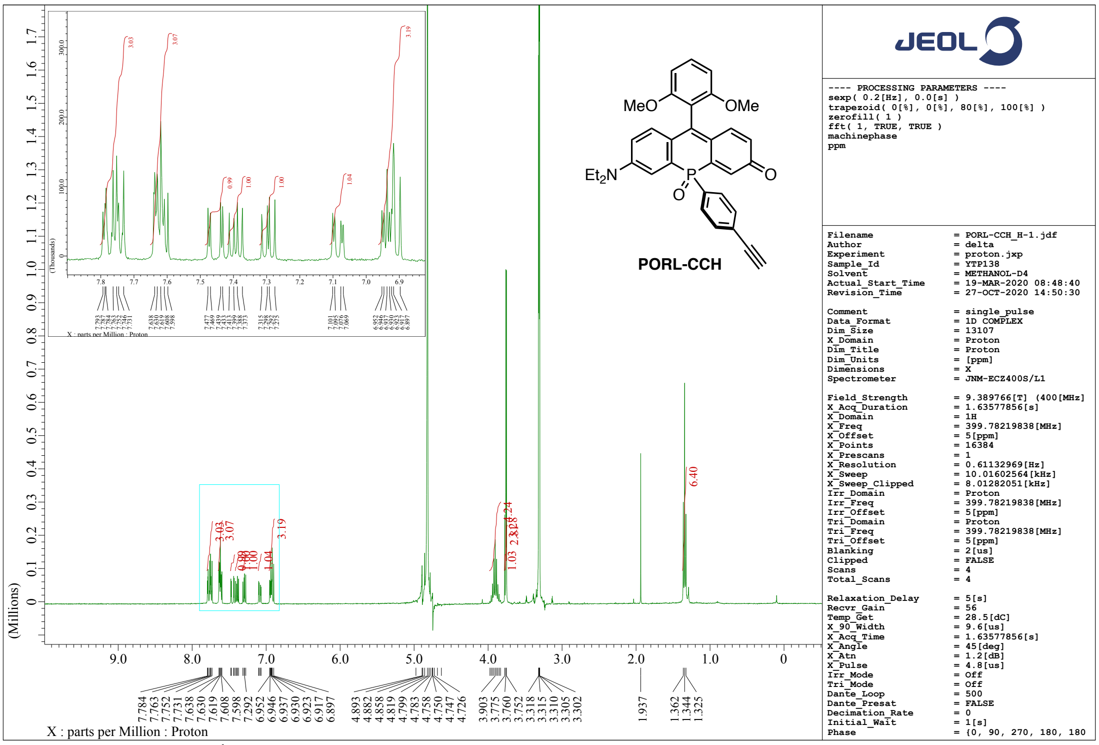

^{1}H NMR spectrum of **PORL-CCH** (400 MHz, CD_3OD)

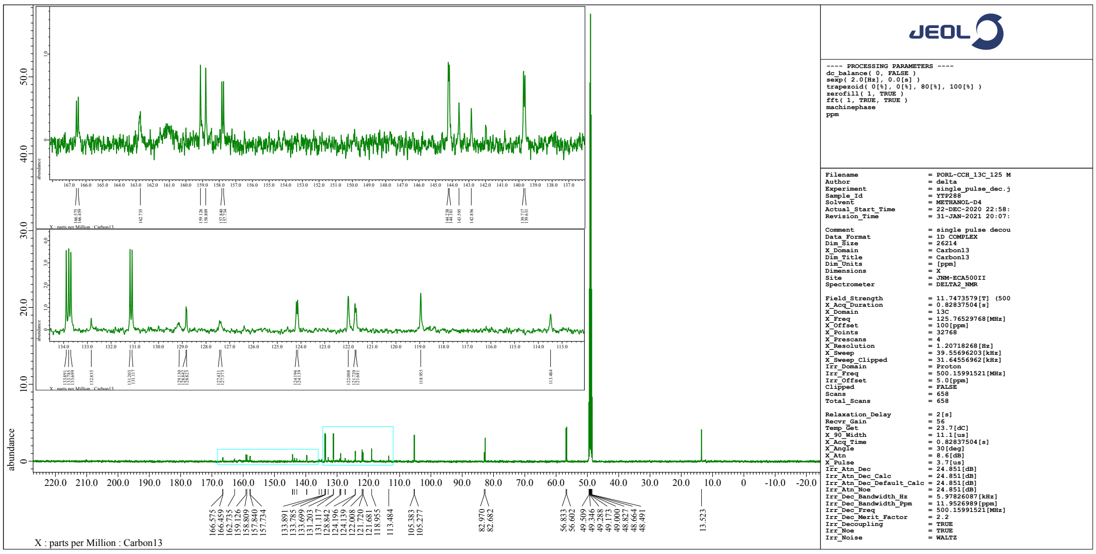

^{13}C NMR spectrum of **PORL-CCH** (125 MHz, CD_3OD)

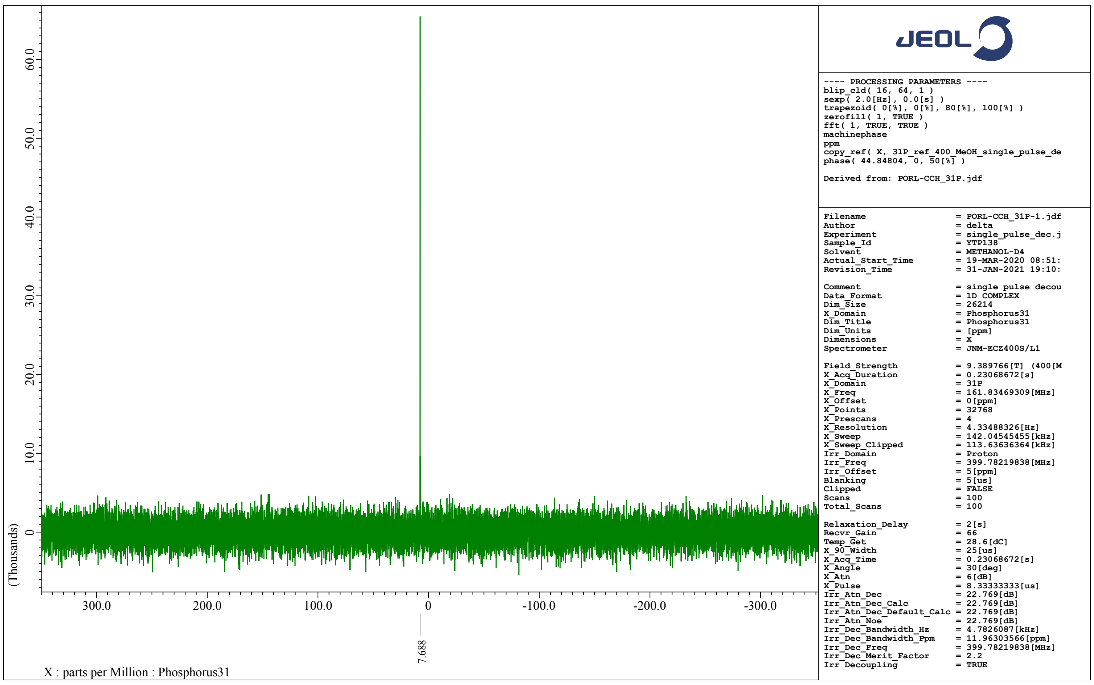

^{31}P NMR spectrum of **PORL-CCH** (162 MHz, CD_3OD)

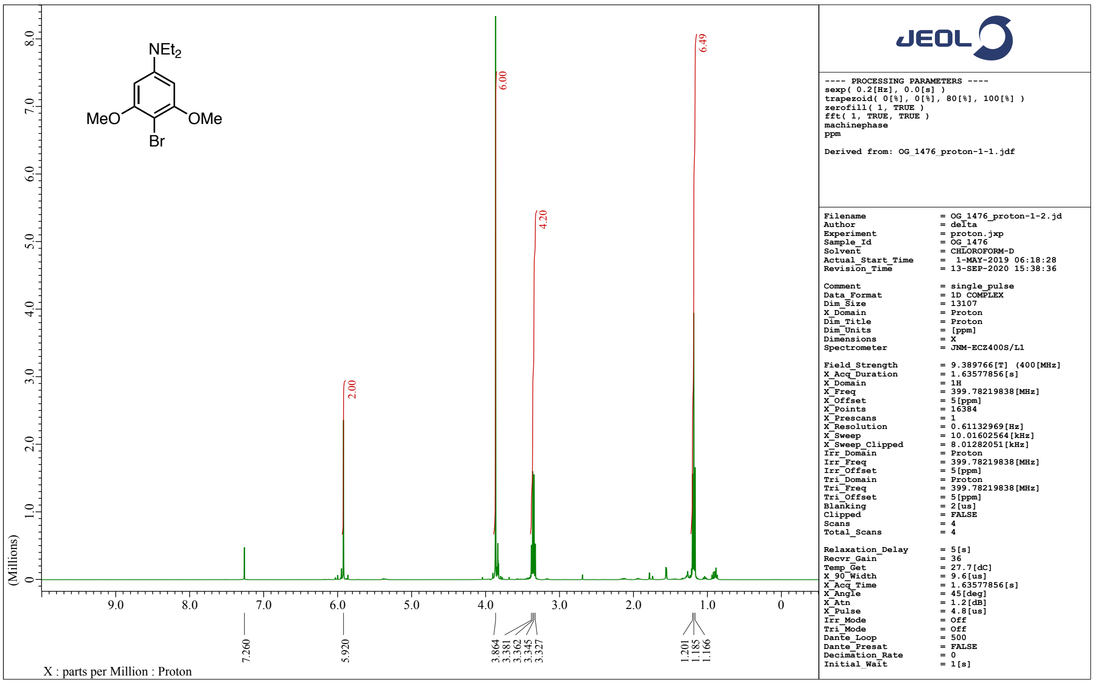

^{1}H NMR spectrum of **7** (400 MHz, $CDCl_3$)

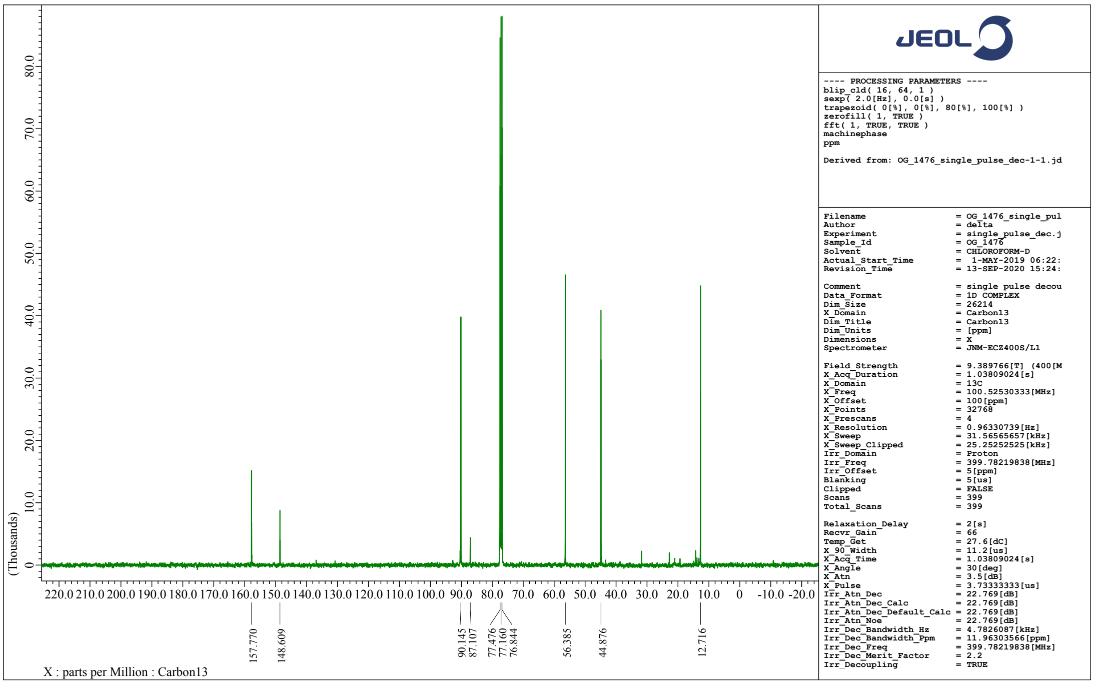

^{13}C NMR spectrum of **7** (100 MHz, $CDCl_3$)

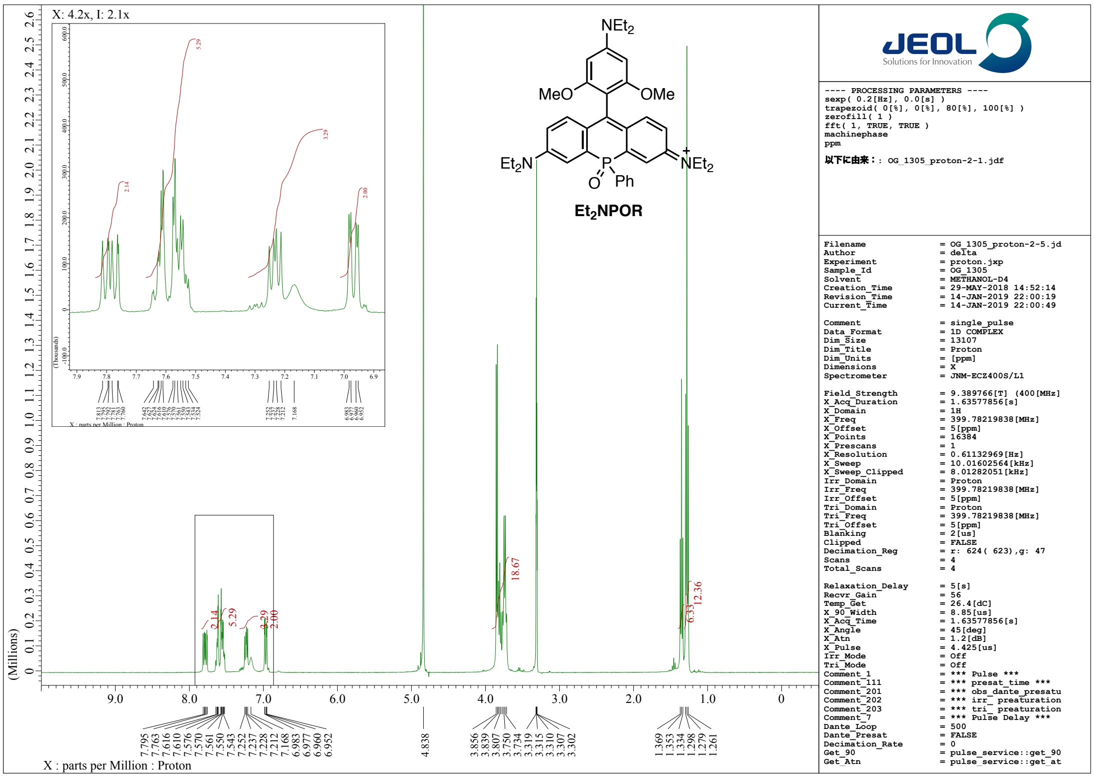

^{1}H NMR spectrum of **Et₂NPOR** (400 MHz, CD₃OD)

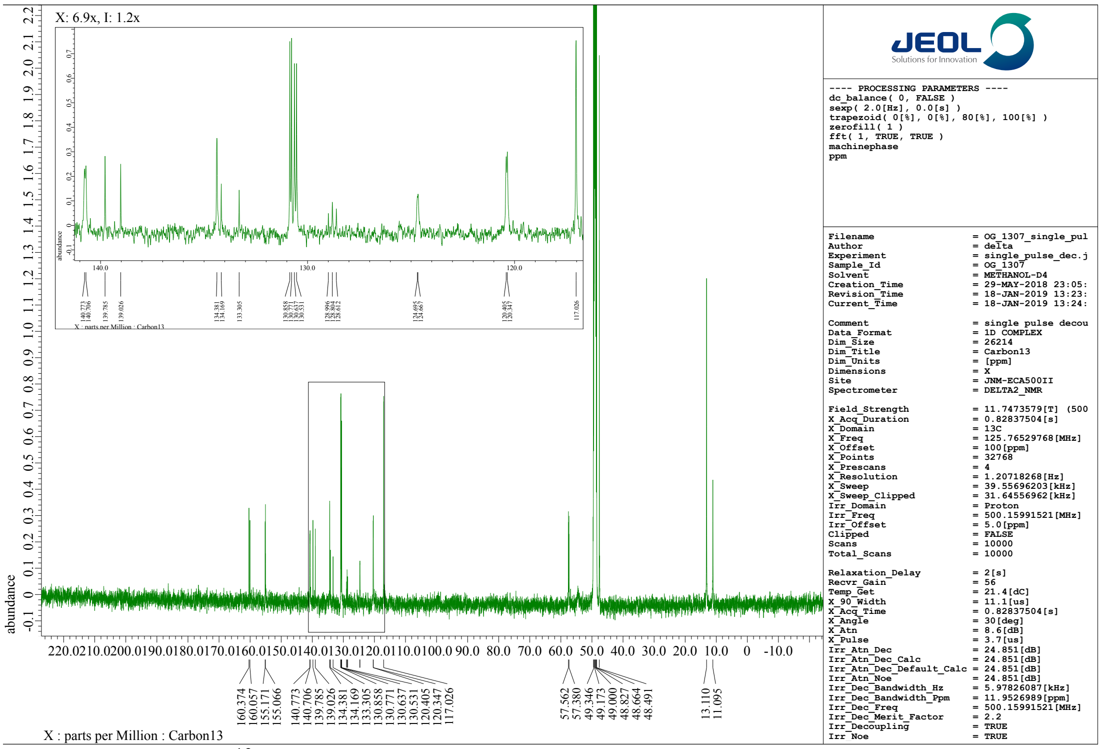

^{13}C NMR spectrum of **Et₂NPOR** (125 MHz, CD₃OD)

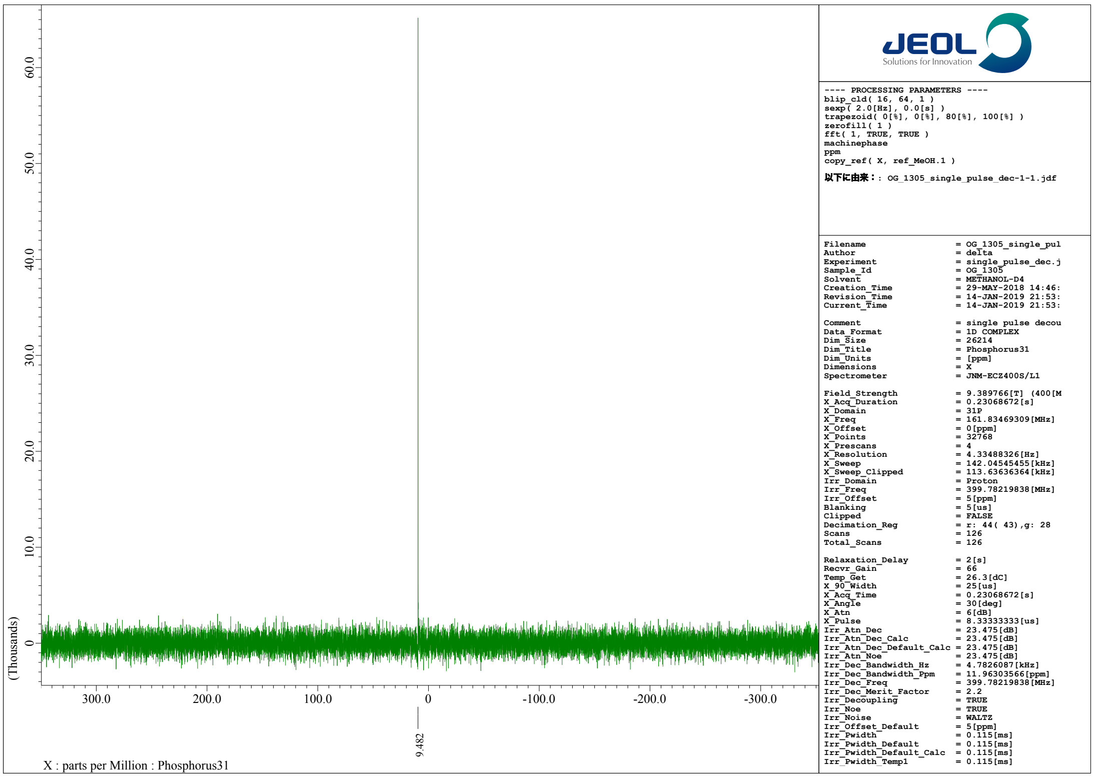

^{31}P NMR spectrum of **Et_2NPOR** (162 MHz, CD_3OD)

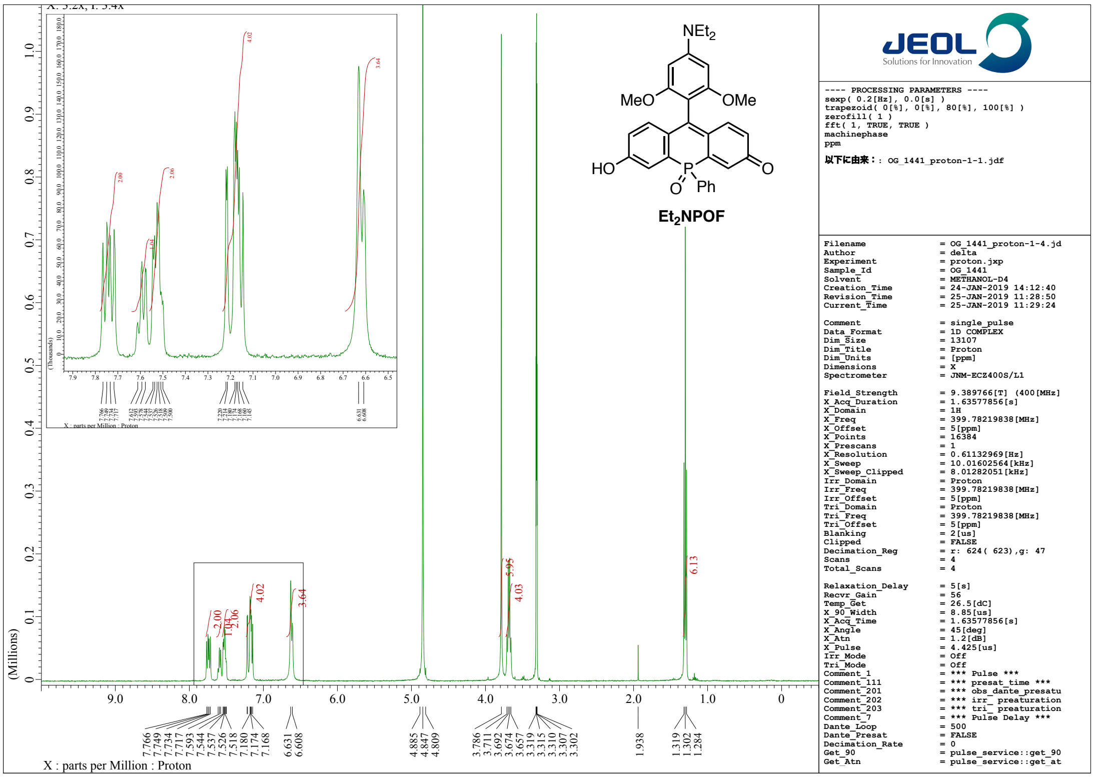

^{1}H NMR spectrum of **Et_2NPOF** (400 MHz, CD_3OD)

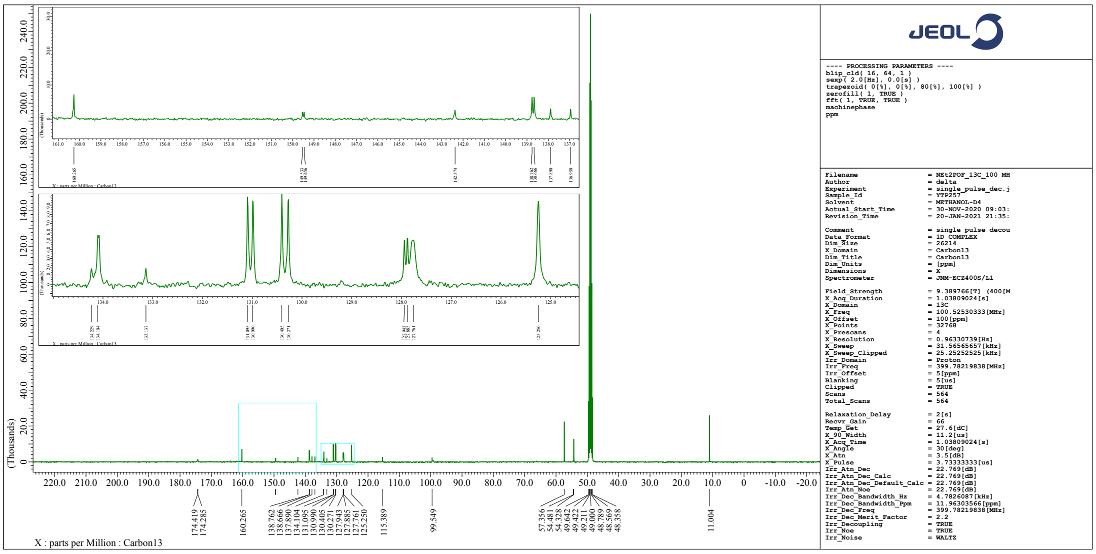

^{13}C NMR spectrum of **Et_2NPOF** (100 MHz, CD_3OD)

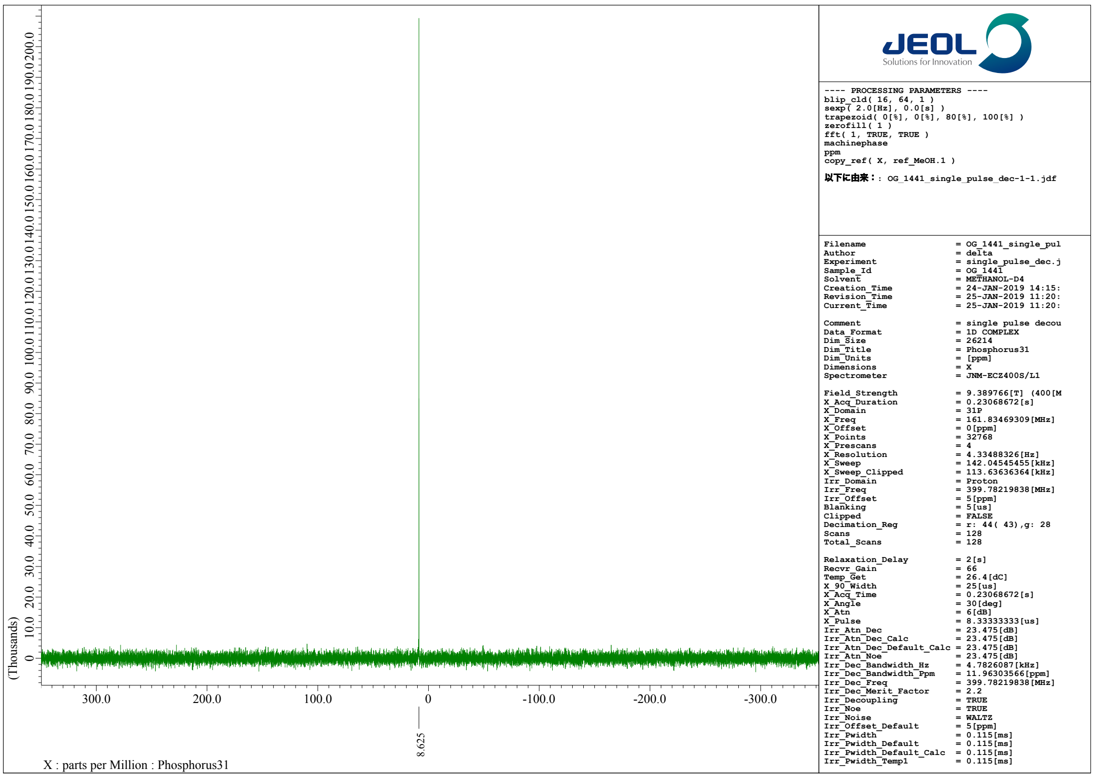

^{31}P NMR spectrum of **Et_2NPOF** (162 MHz, CD_3OD)

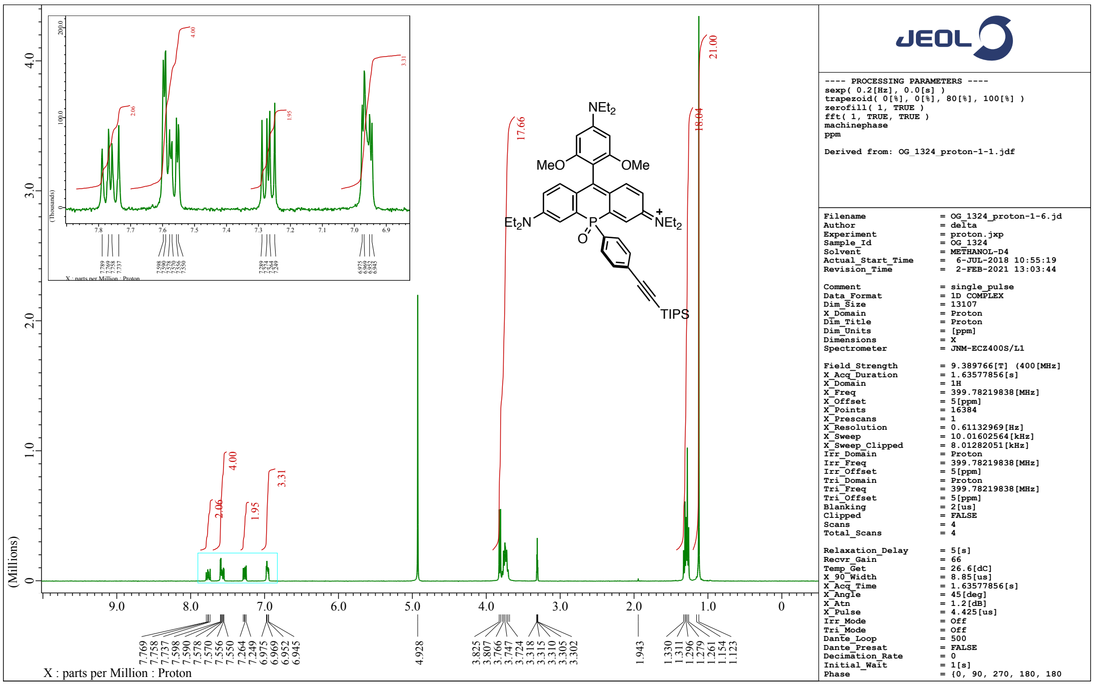

^{1}H NMR spectrum of **8** (400 MHz, CD_3OD)

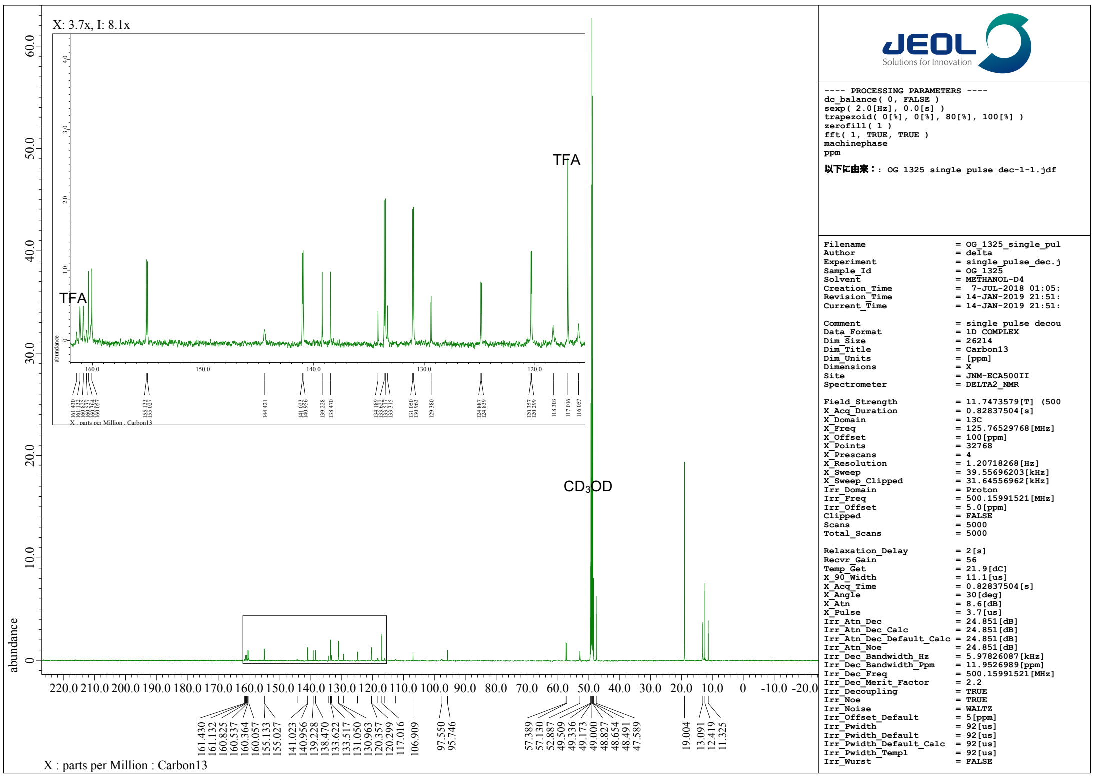

^{13}C NMR spectrum of **8** (125 MHz, CD_3OD)

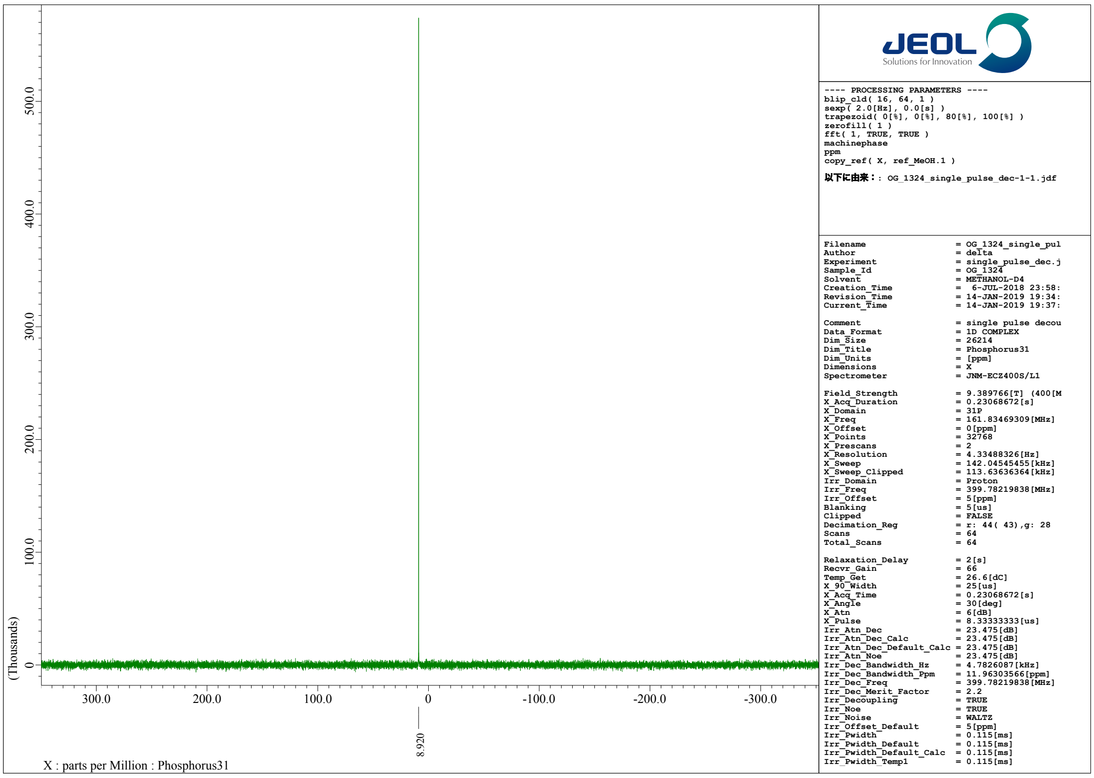

^{31}P NMR spectrum of **8** (162 MHz, CD_3OD)

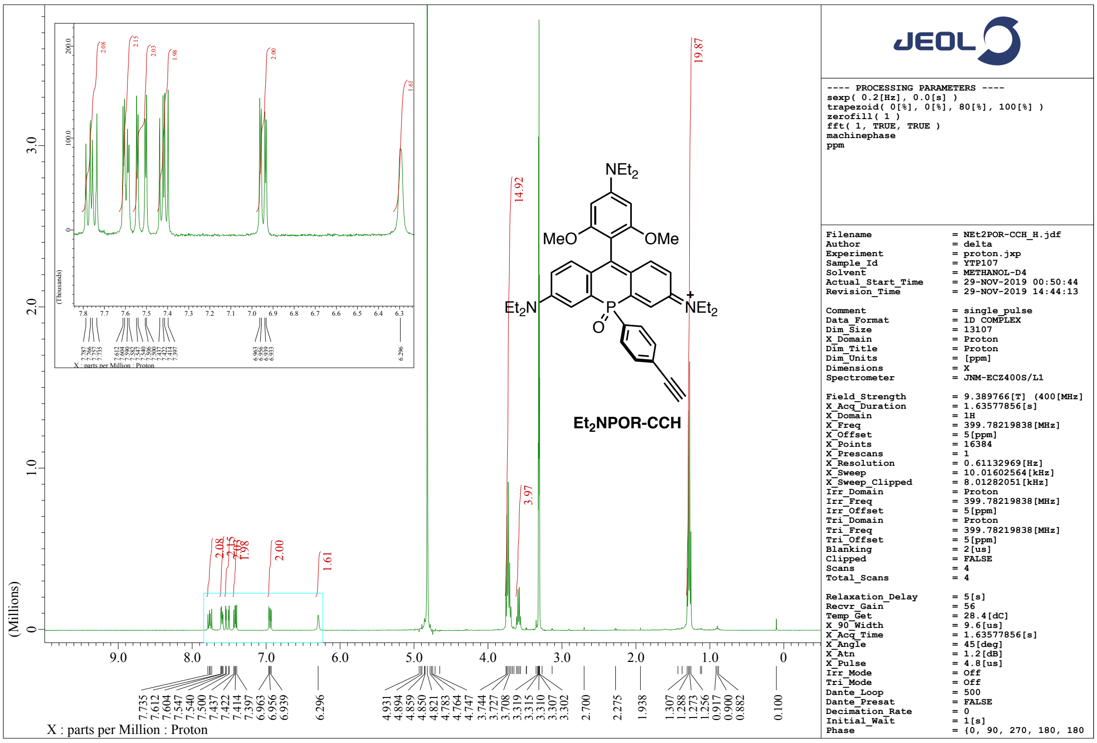

^{1}H NMR spectrum of **Et_2NPOR- CCH** (400 MHz, CD_3OD)

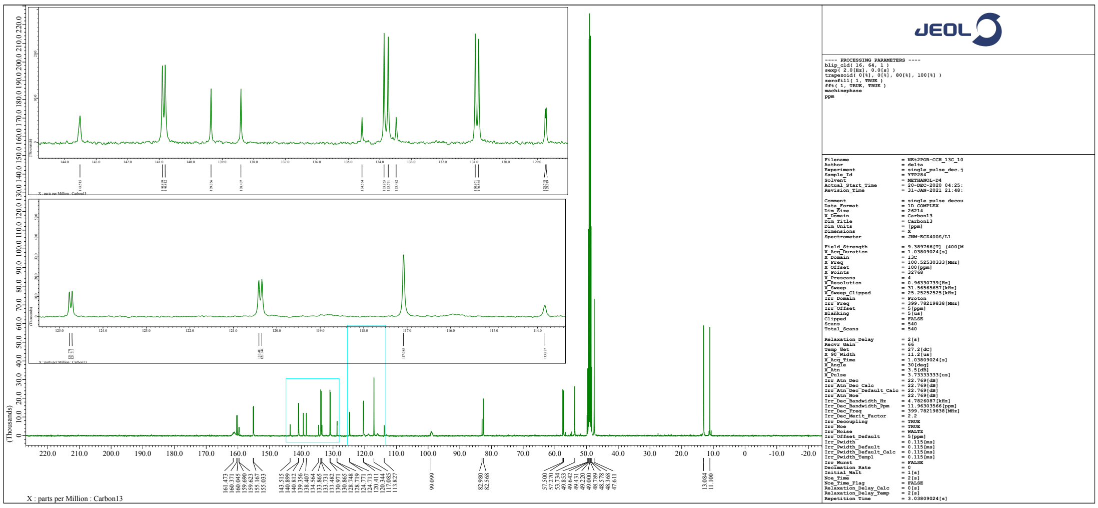

^{13}C NMR spectrum of **Et_2NPOR-CCH** (100 MHz, CD_3OD)

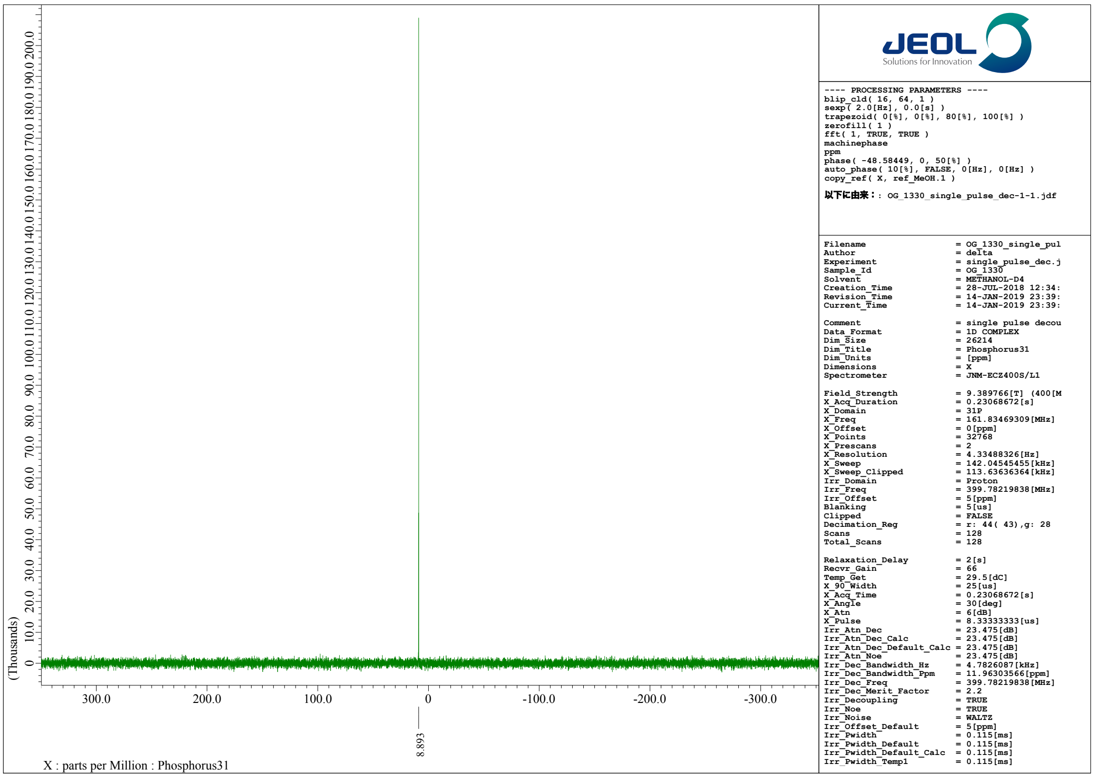

^{31}P NMR spectrum of **Et_2NPOR-CCH** (162 MHz, CD_3OD)

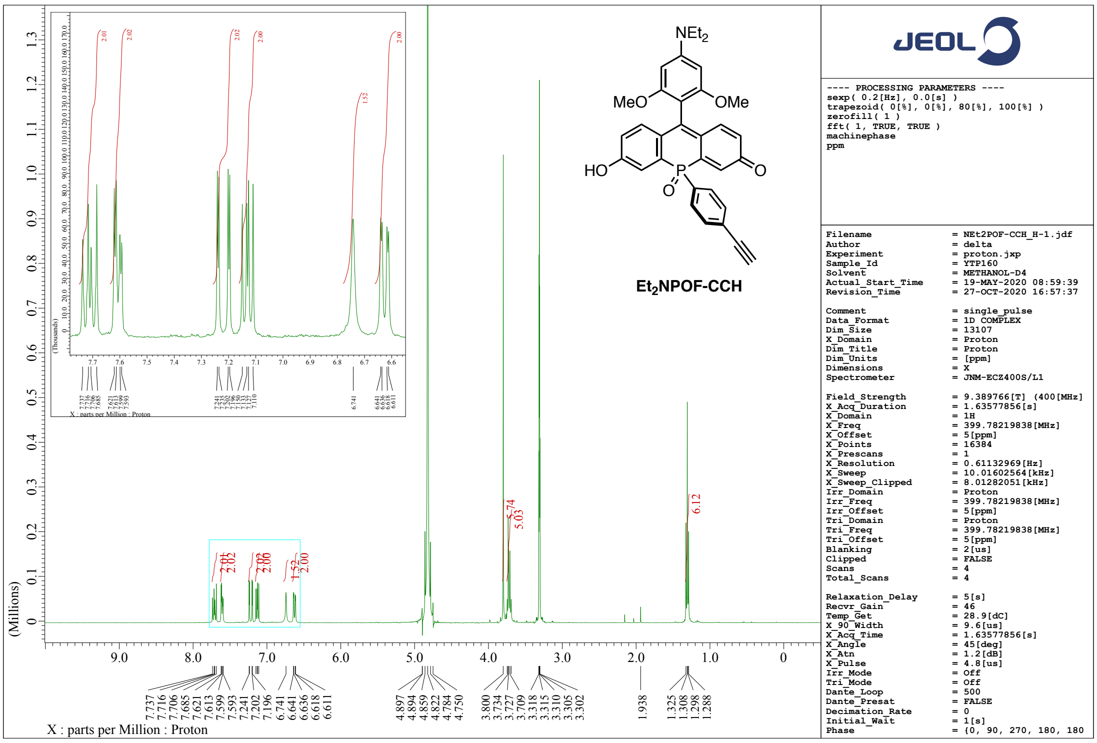

^{1}H NMR spectrum of **Et_2NPOF-CCH** (400 MHz, CD_3OD)

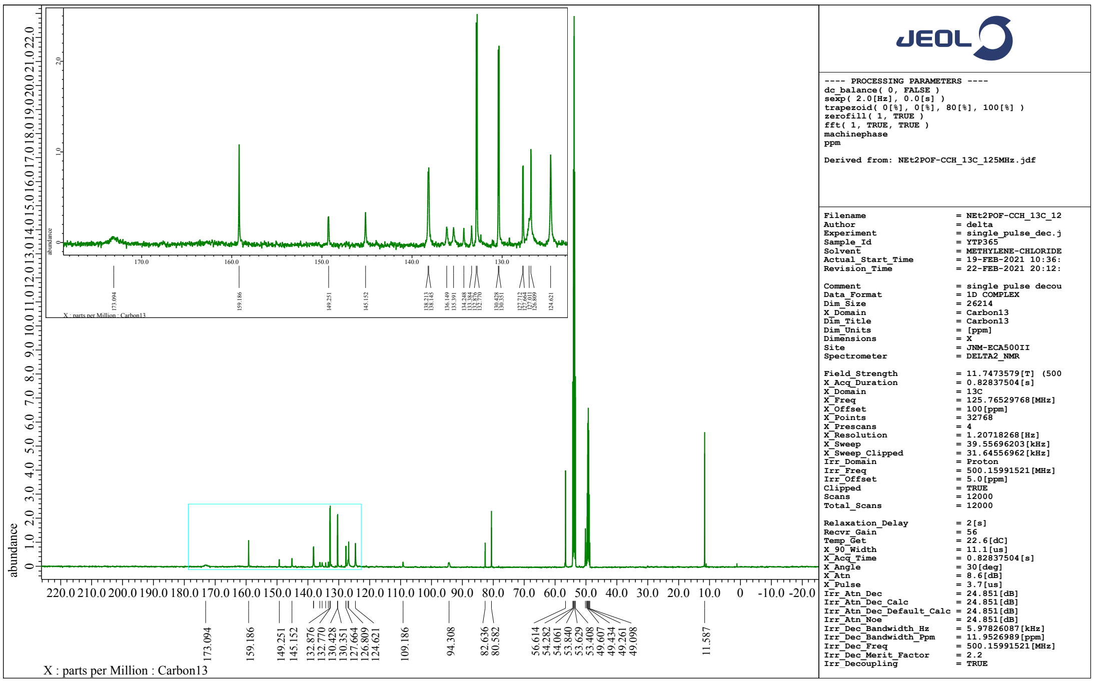

^{13}C NMR spectrum of **Et_2NPOF-CCH** (125 MHz, CD_2Cl_2/CD_3OD = 9/1)

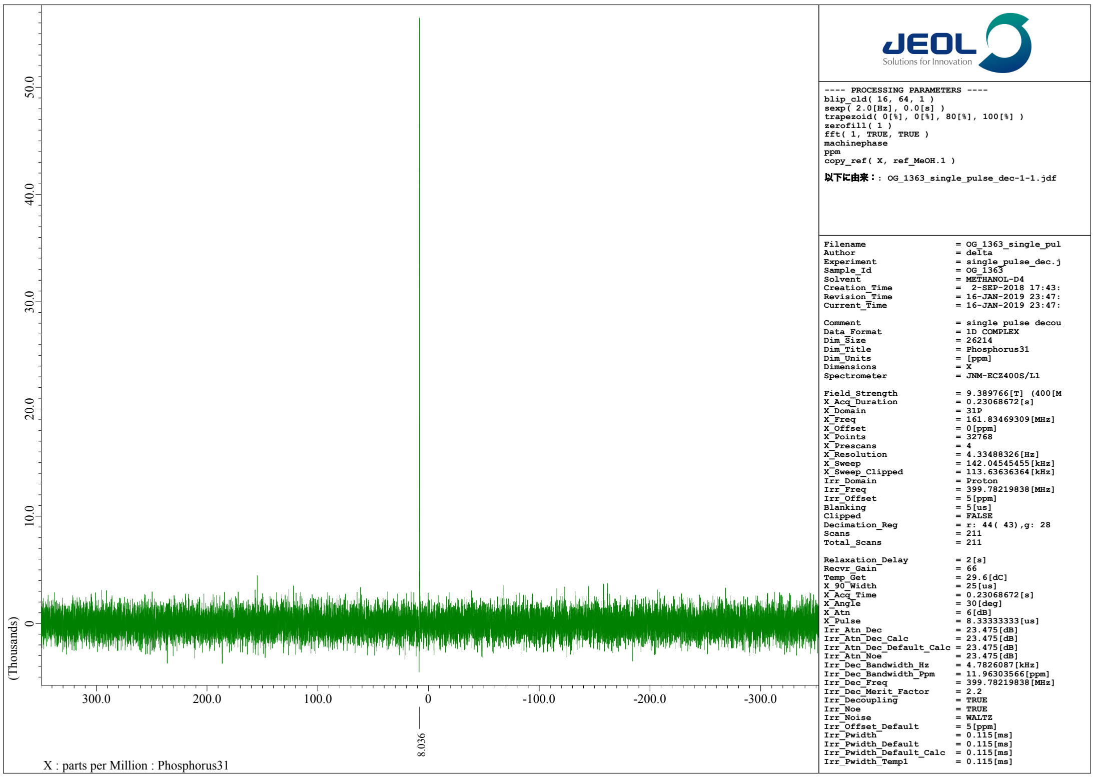

^{31}P NMR spectrum of **Et_2NPOF-CCH** (162 MHz, CD_3OD)

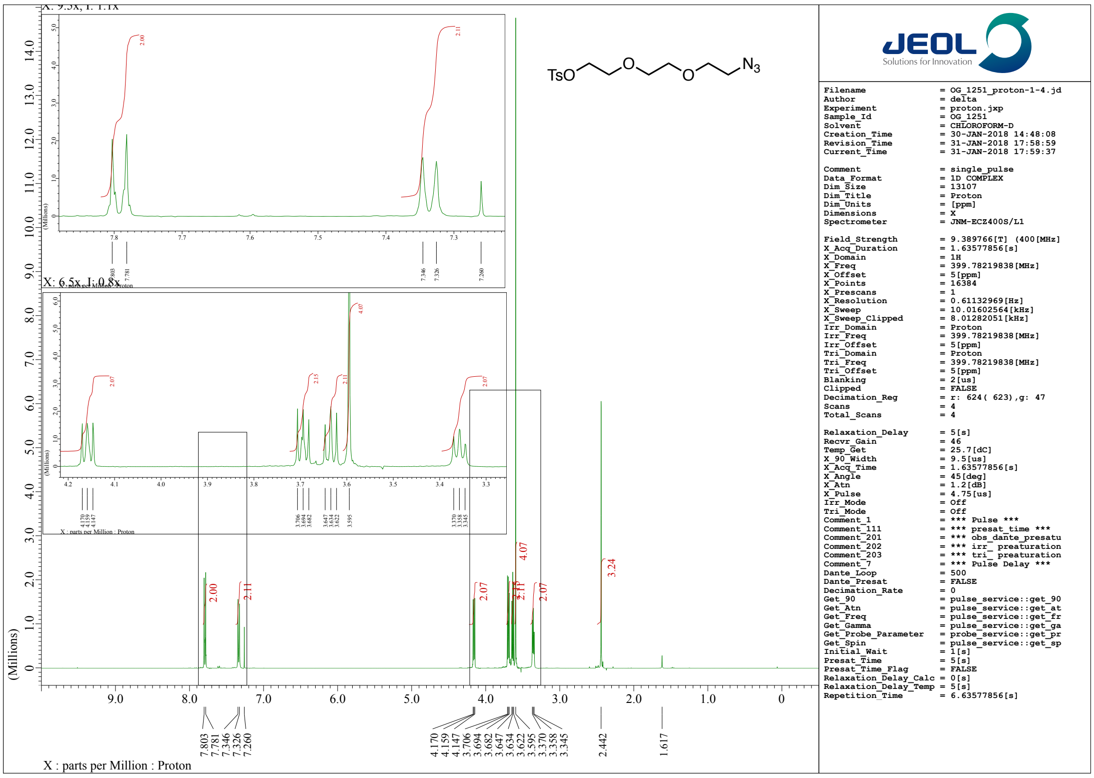

^{1}H NMR spectrum of **9** (400 MHz, $CDCl_3$)

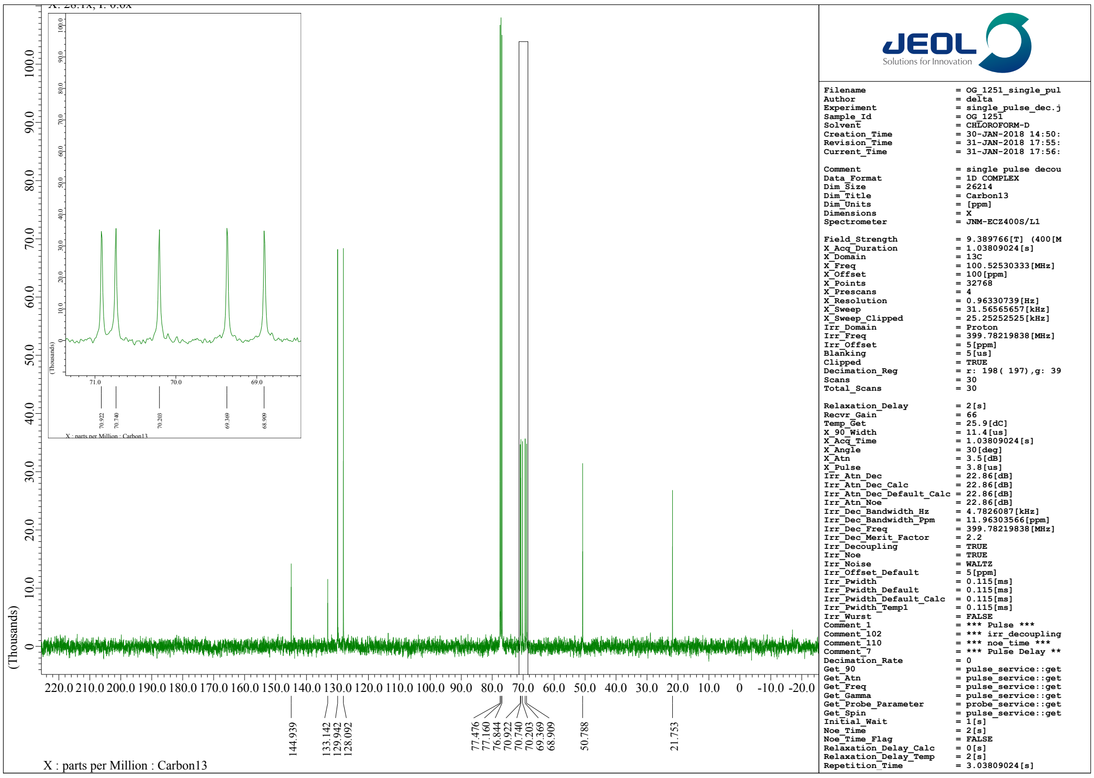

^{13}C NMR spectrum of **9** (100 MHz, $CDCl_3$)

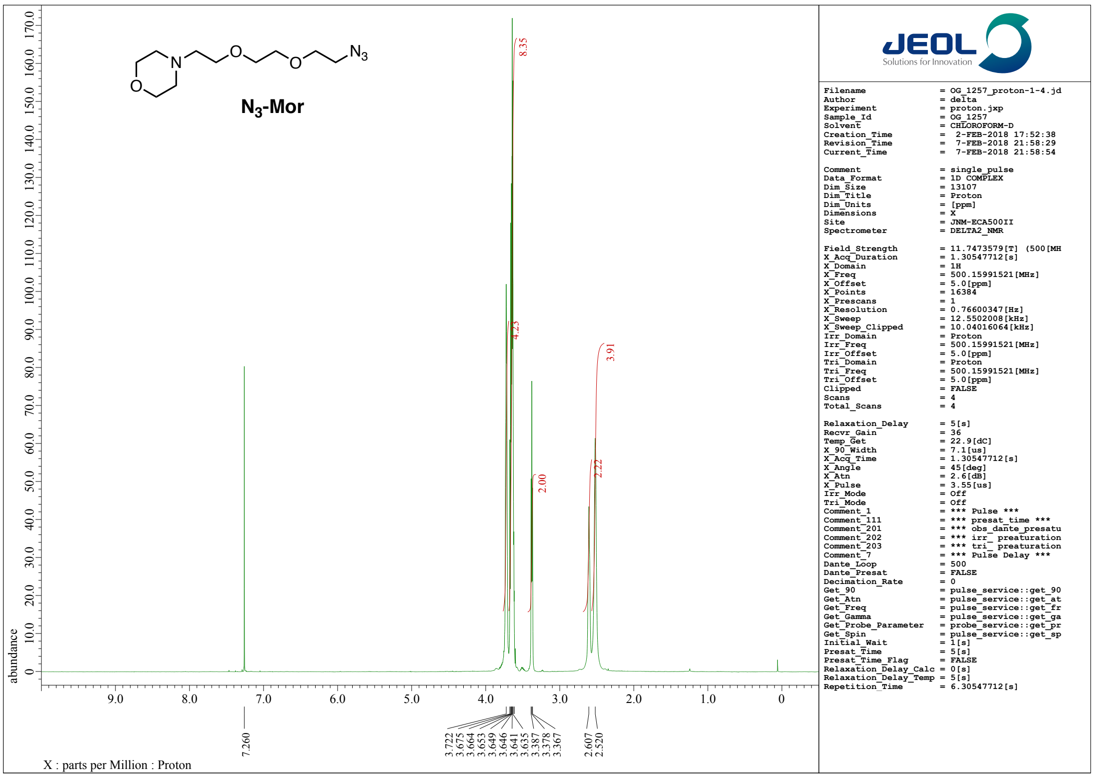

^{1}H NMR spectrum of **N_3-Mor** (500 MHz, $CDCl_3$)

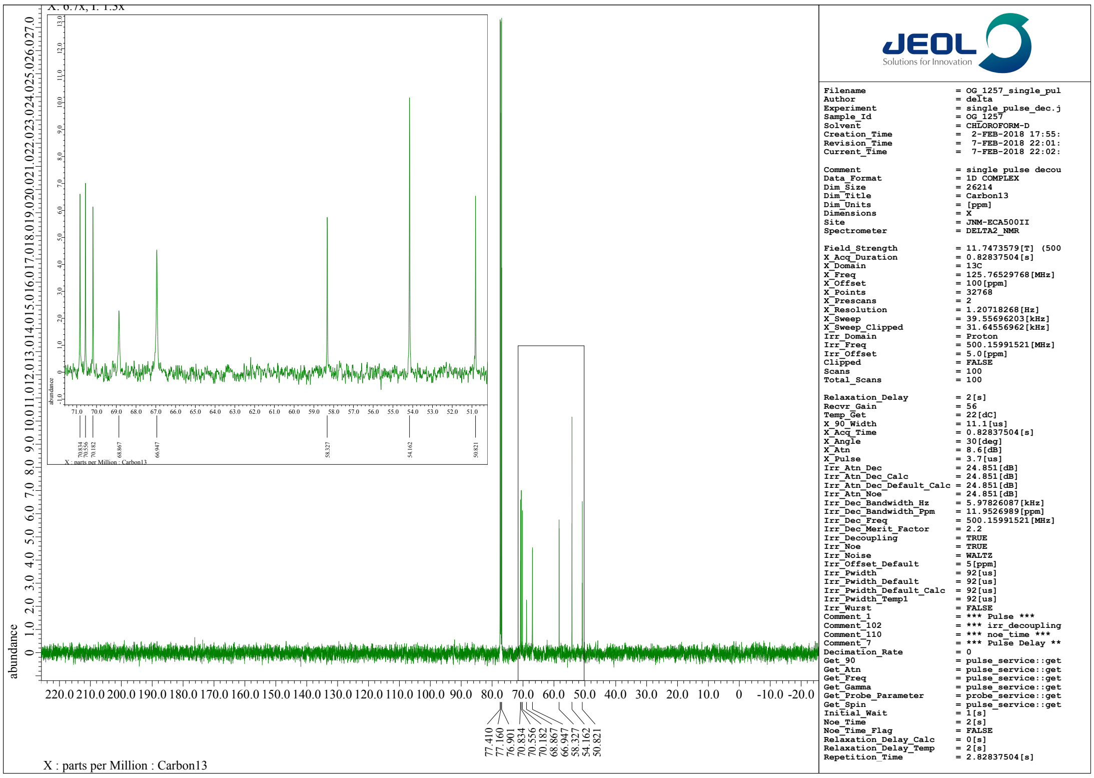

^{13}C NMR spectrum of **N_3-Mor** (125 MHz, $CDCl_3$)

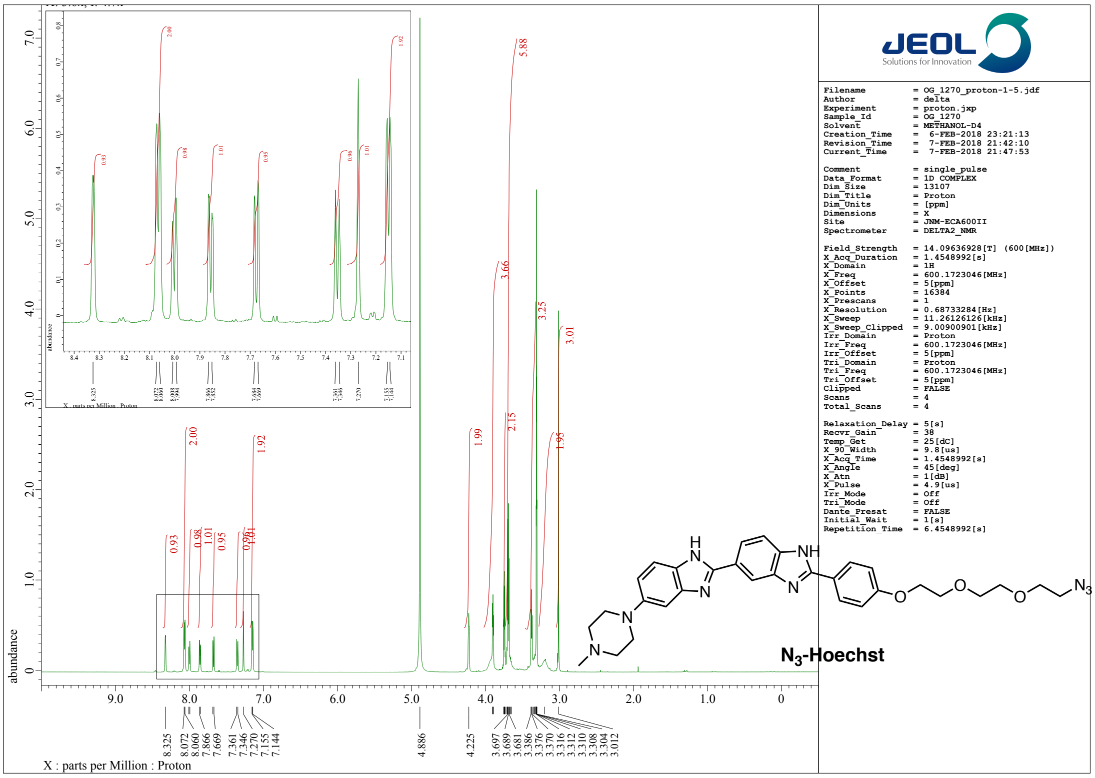

^{1}H NMR spectrum of **N3-Hoechst** (600 MHz, CD_3OD)

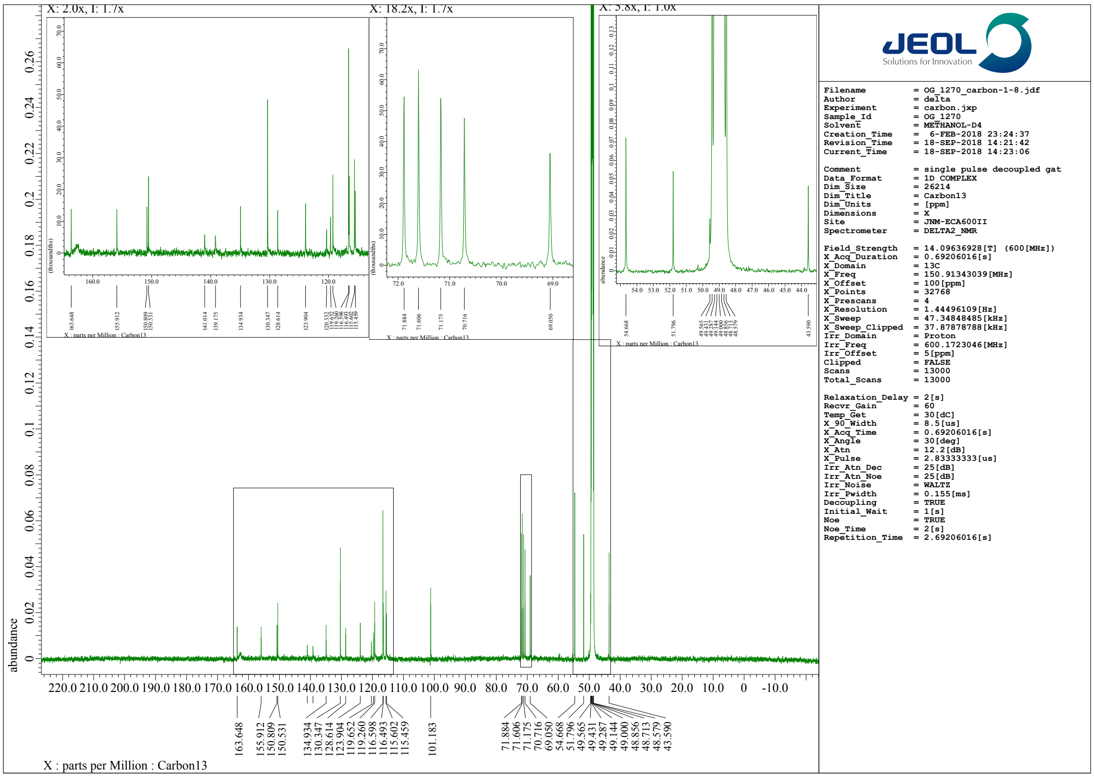

^{13}C NMR spectrum of **N3-Hoechst** (150 MHz, CD_3OD)

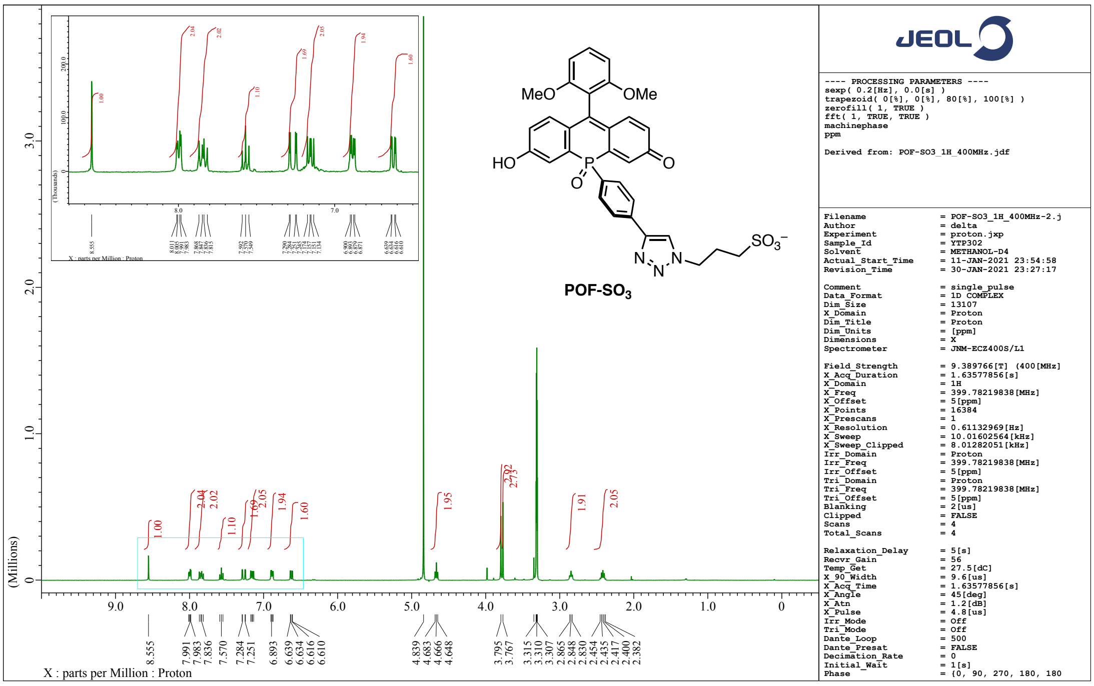

^{1}H NMR spectrum of **POF-SO3** (400 MHz, CD_3OD)

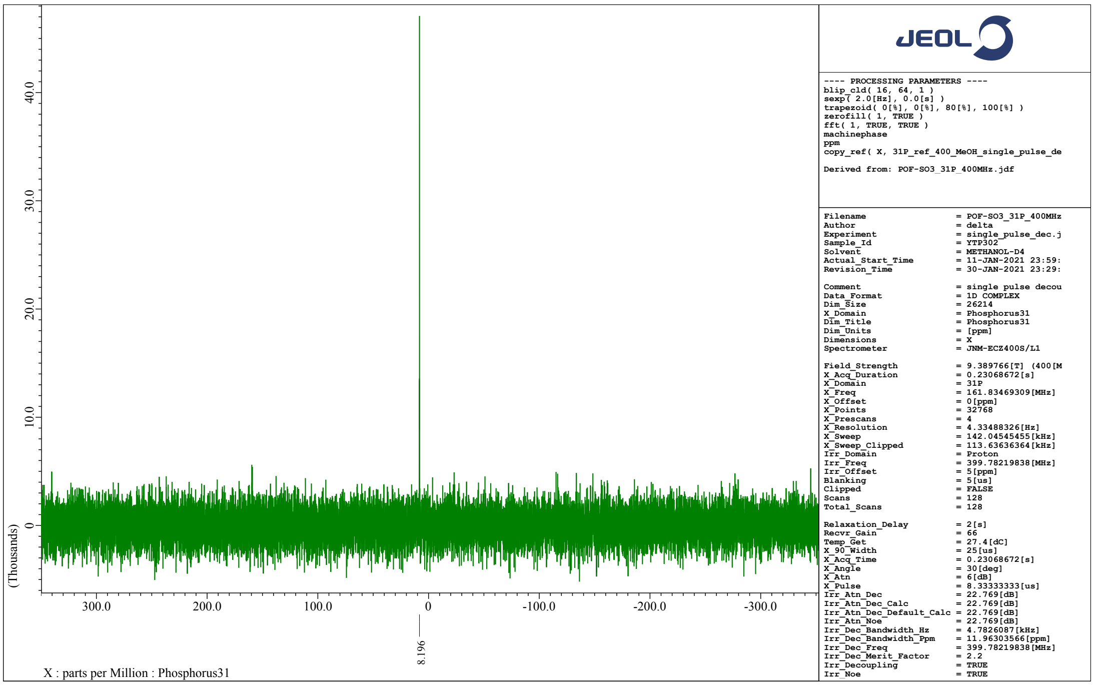

^{31}P NMR spectrum of **POF-SO3** (162 MHz, CD_3OD)

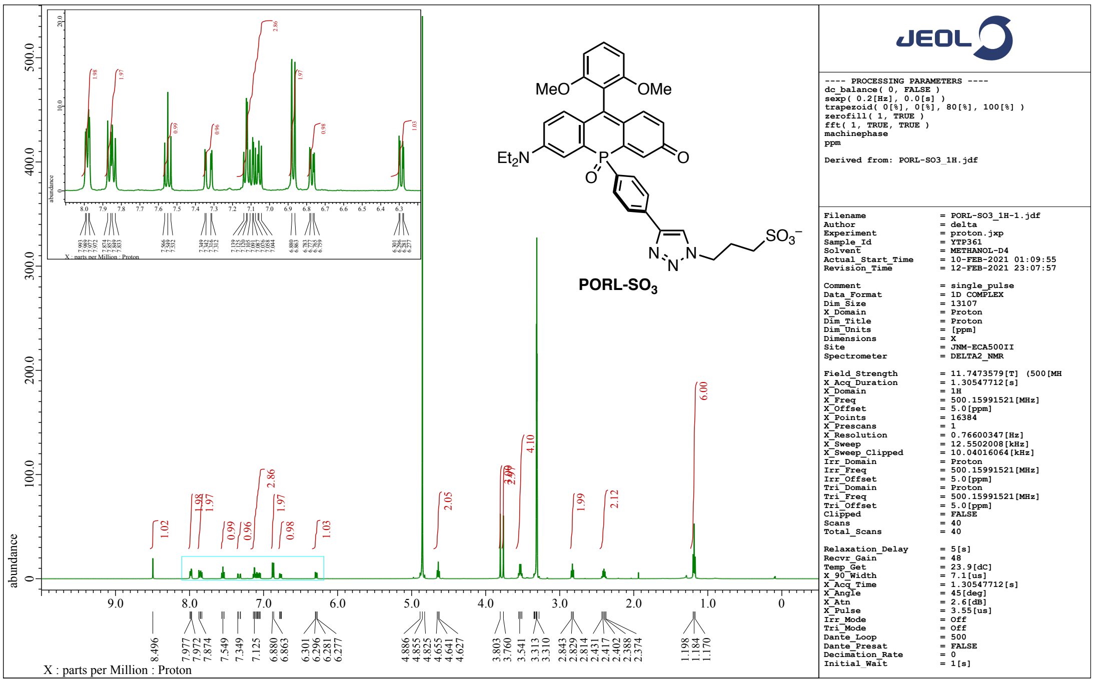

H NMR spectrum of **PORL-SO3** (500 MHz, CD_3OD)

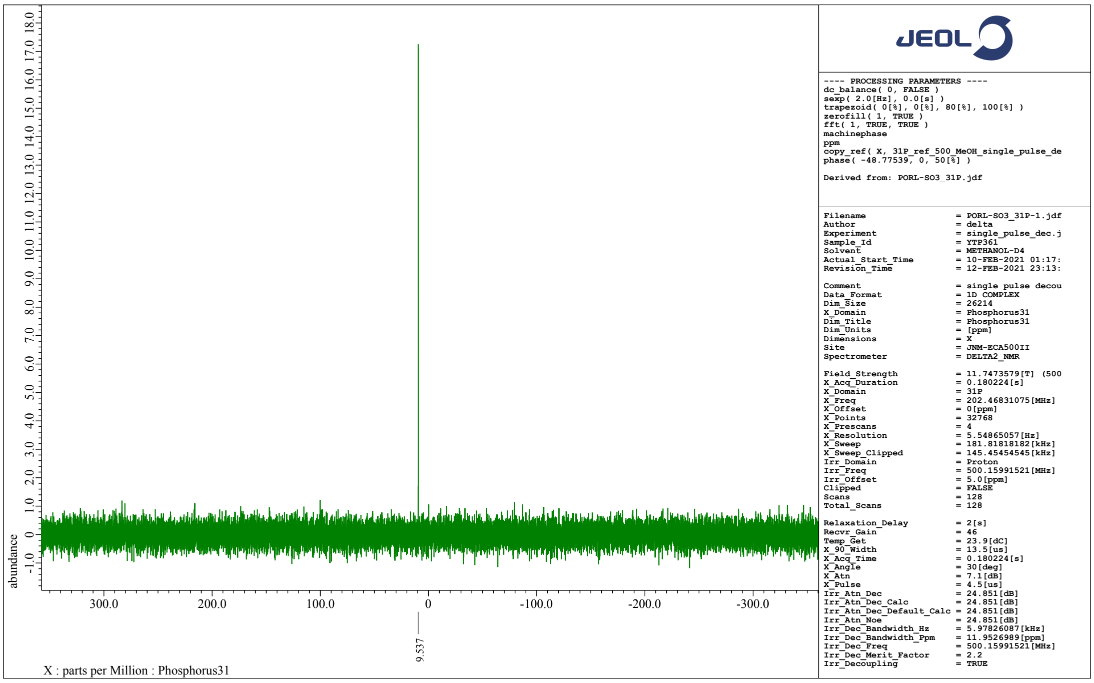

^{31}P NMR spectrum of **PORL-SO3** (202 MHz, CD_3OD)

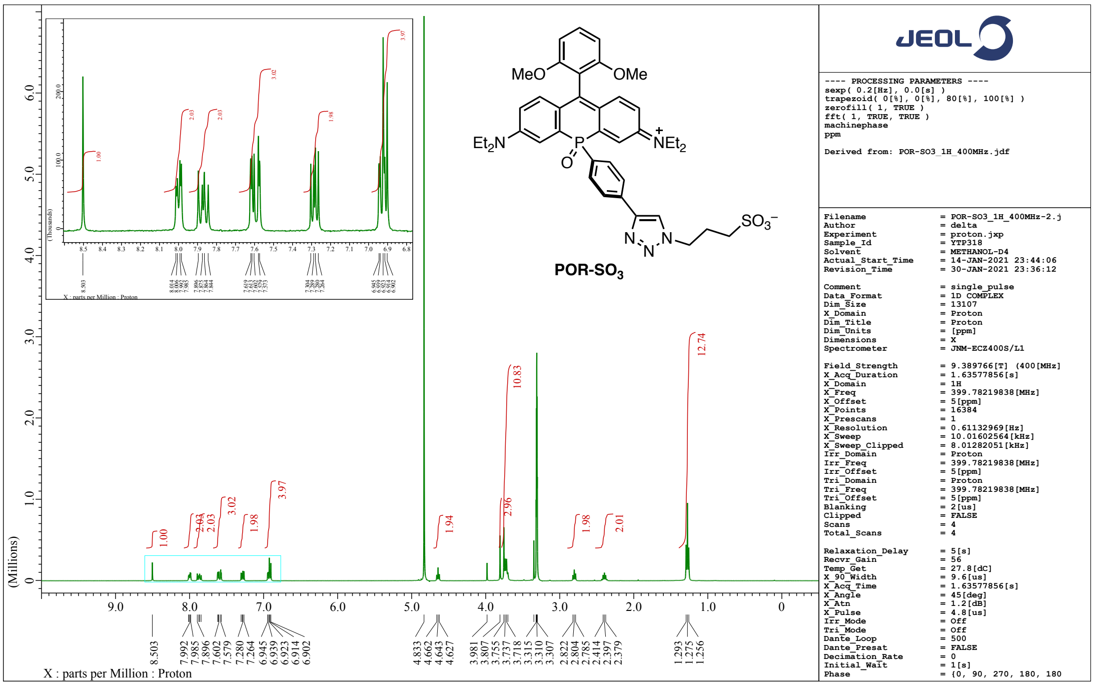

^{1}H NMR spectrum of **POR-SO$_3$** (400 MHz, CD$_3$OD)

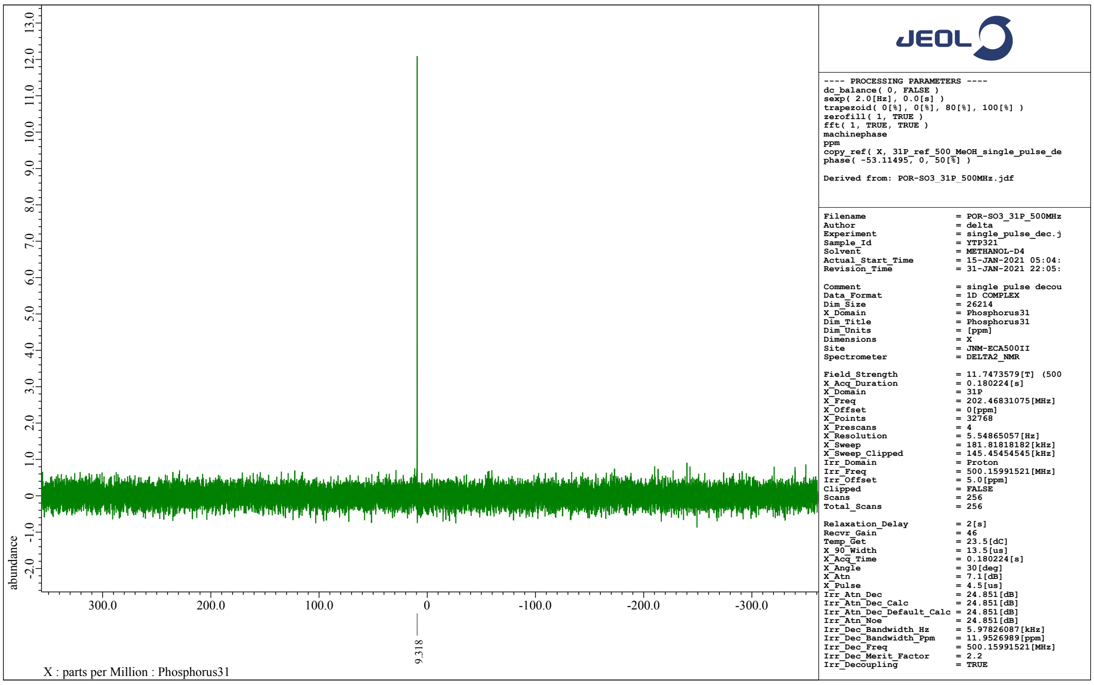

^{31}P NMR spectrum of **POR-SO$_3$** (202 MHz, CD$_3$OD)

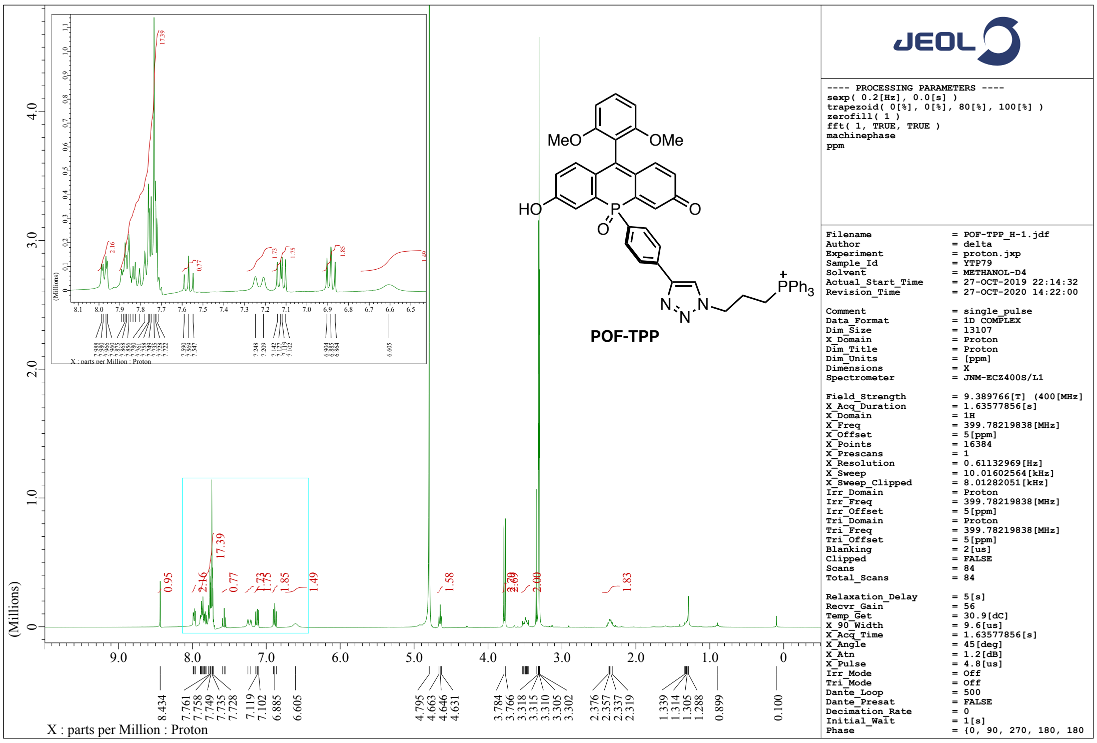

^{1}H NMR spectrum of **POF-TPP** (400 MHz, CD_3OD)

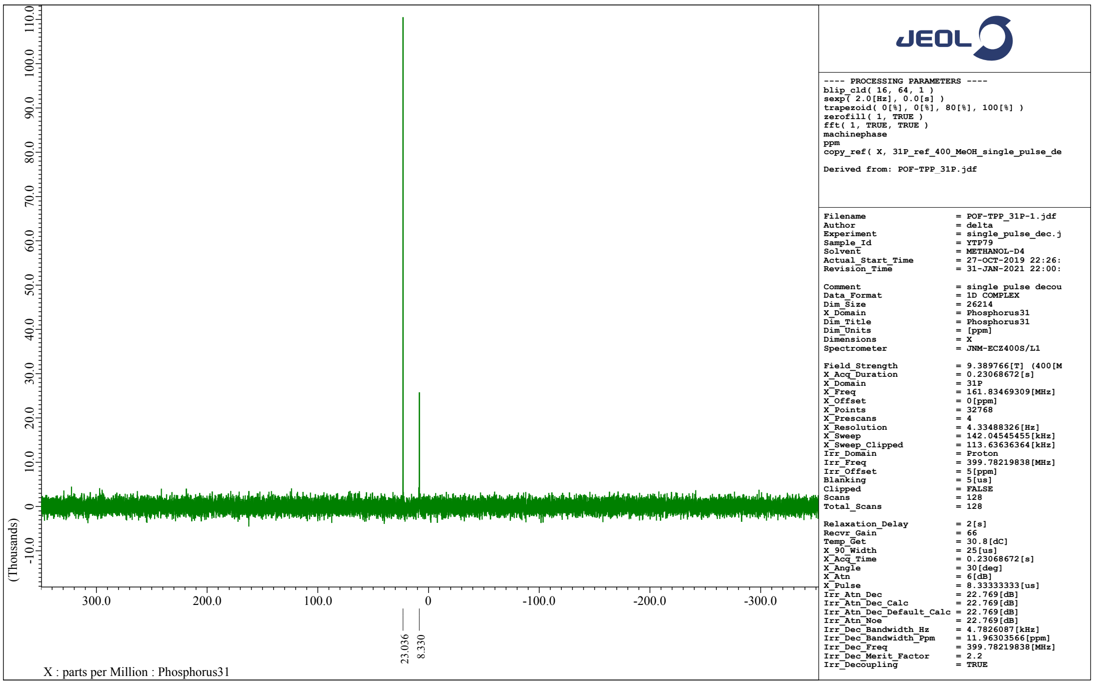

^{31}P NMR spectrum of **POF-TPP** (162 MHz, CD_3OD)

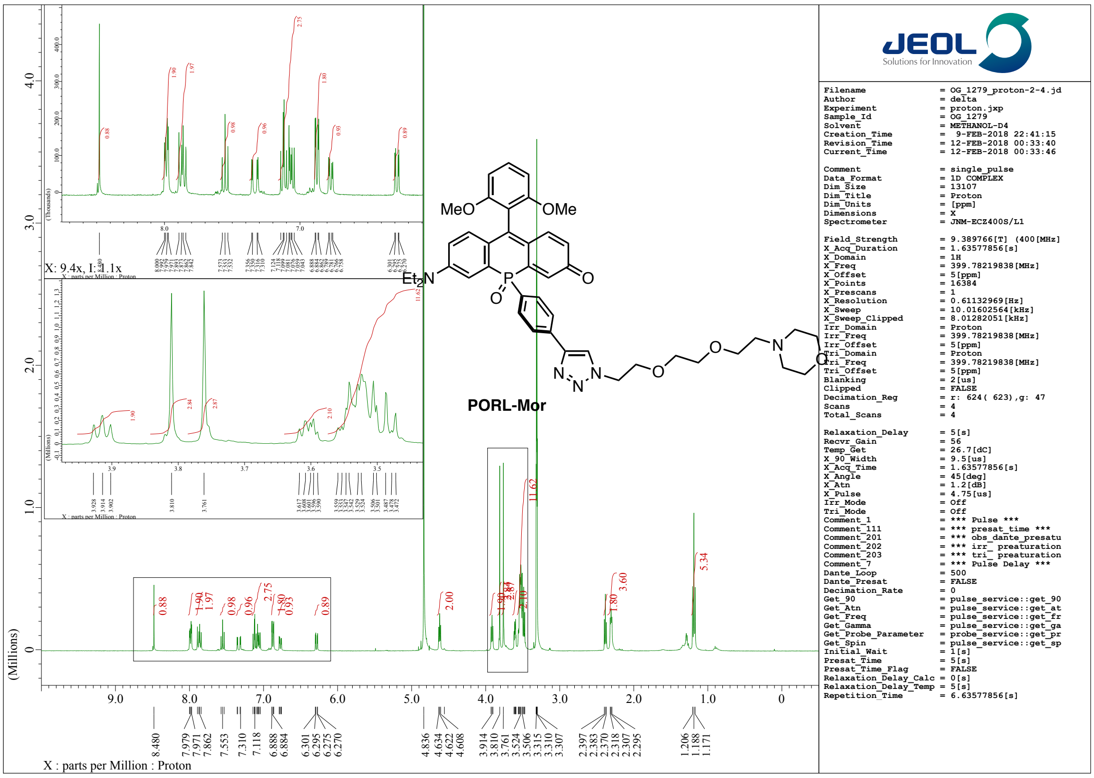

^{1}H NMR spectrum of **PORL-Mor** (400 MHz, CD_3OD)

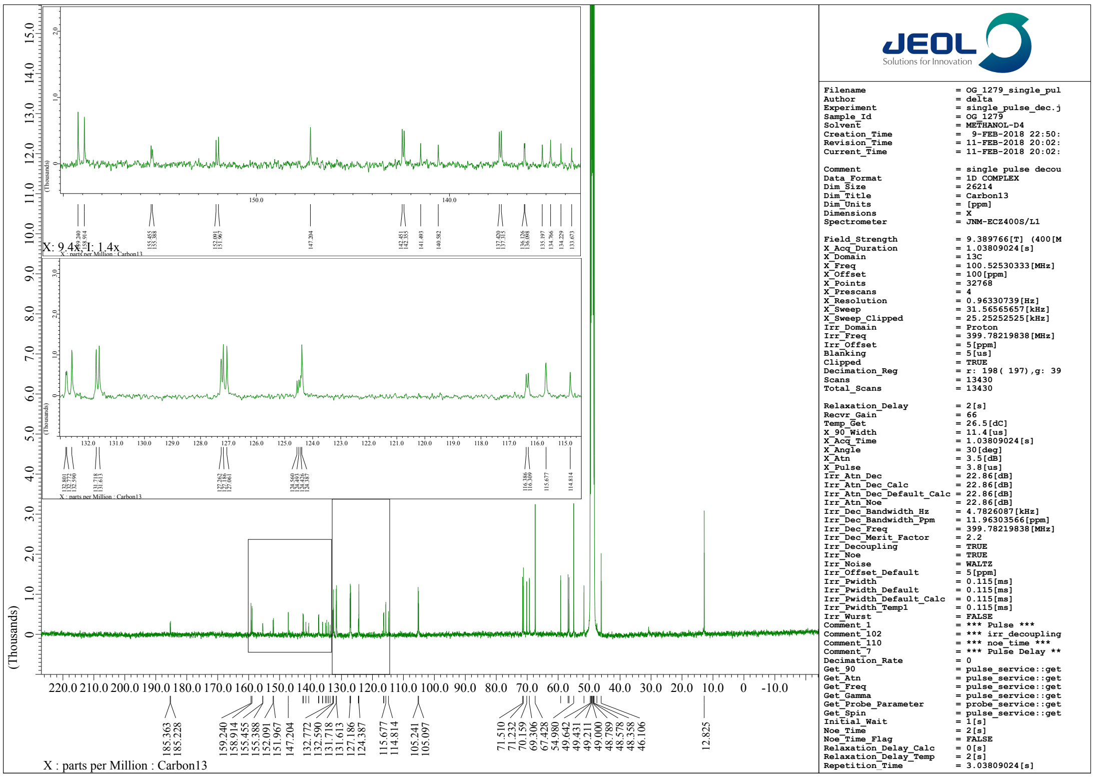

^{13}C NMR spectrum of **PORL-Mor** (100 MHz, CD_3OD)

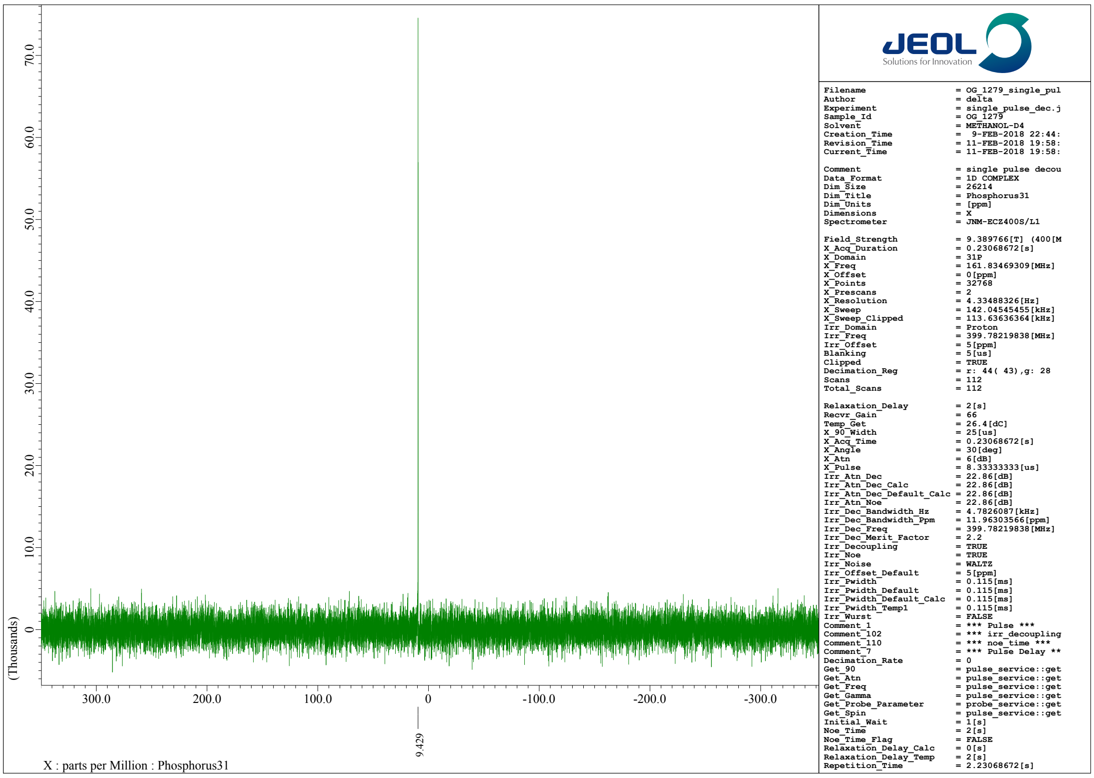

^{31}P NMR spectrum of **PORL-Mor** (162 MHz, CD_3OD)

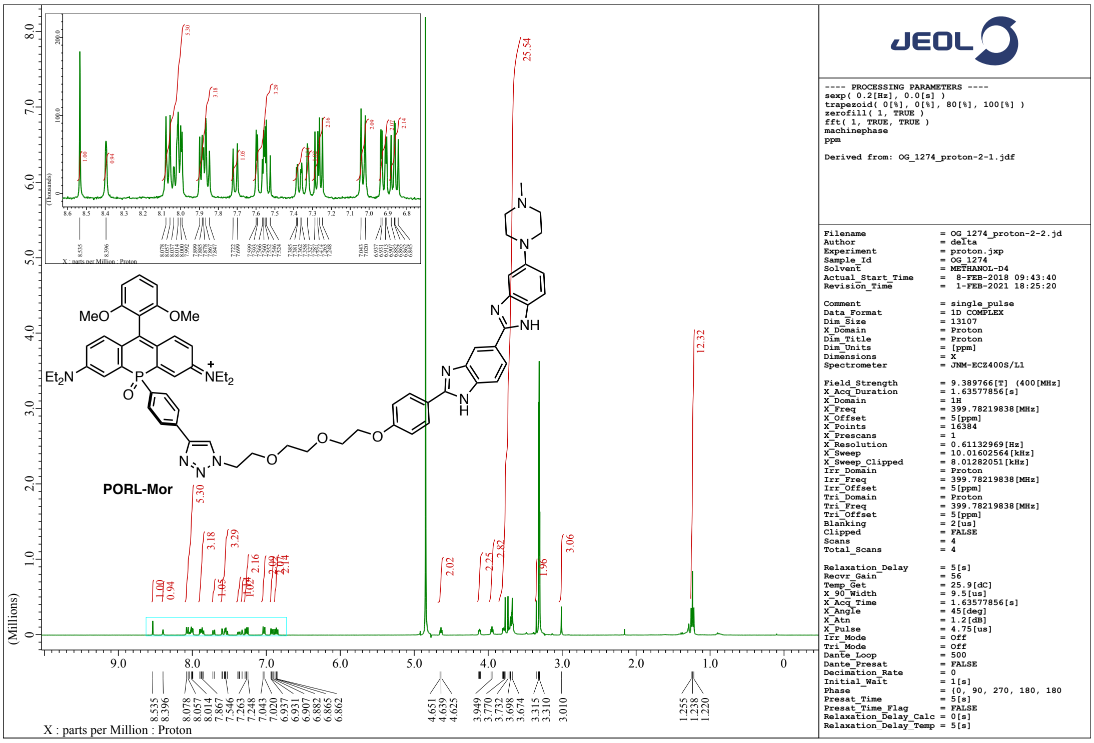

^{1}H NMR spectrum of **POR-Hoechst** (400 MHz, CD_3OD)

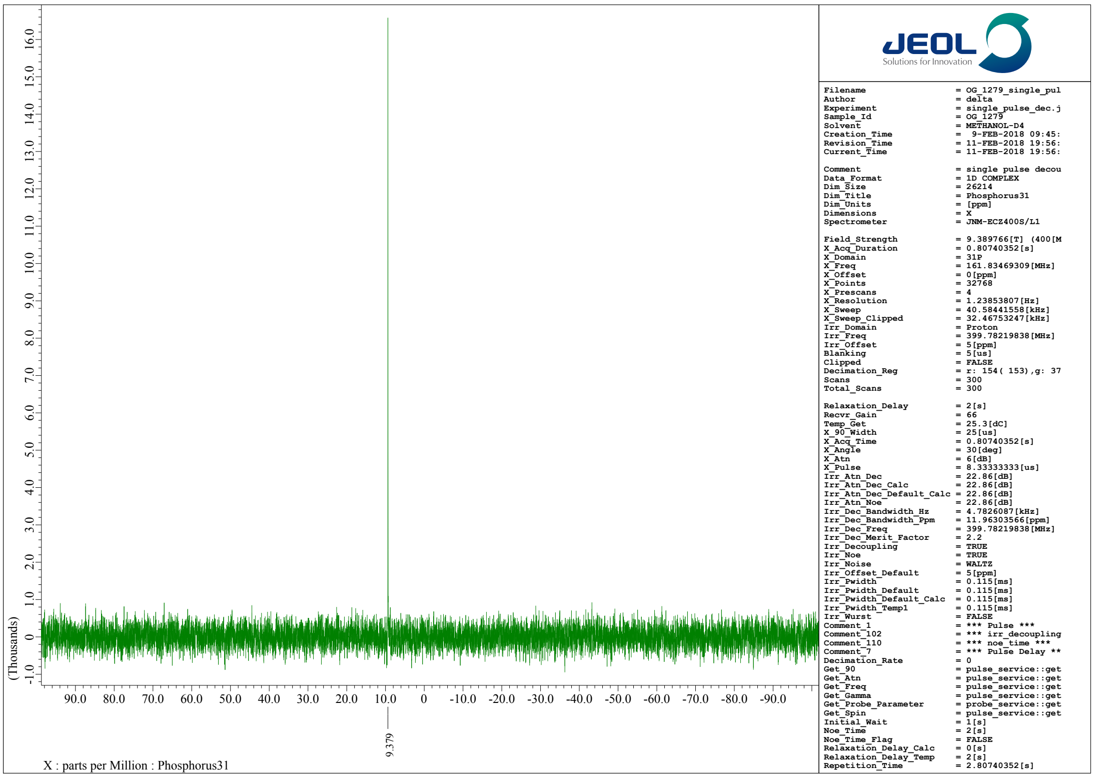

^{31}P NMR spectrum of **POR-Hoechst** (162 MHz, CD_3OD)
